# Low level of anthropization linked to harsh vertebrate biodiversity declines in Amazonia

**DOI:** 10.1038/s41467-022-30842-2

**Published:** 2022-06-07

**Authors:** Isabel Cantera, Opale Coutant, Céline Jézéquel, Jean-Baptiste Decotte, Tony Dejean, Amaia Iribar, Régis Vigouroux, Alice Valentini, Jérôme Murienne, Sébastien Brosse

**Affiliations:** 1 Laboratoire Evolution et Diversité Biologique (UMR 5174), CNRS, IRD, Université Paul Sabatier, 118 route de Narbonne, 31062 Toulouse, France; 2 Department of Environmental Science and Policy, Università degli Studi di Milano. Via Celoria 10, 20133 Milano, Italy; 3 VIGILIFE, 17 rue du Lac Saint-André Savoie Technolac—BP 274, 73375 Le Bourget-du-Lac, France; 4 SPYGEN, 17 rue du Lac Saint-André Savoie Technolac—BP 274, 73375 Le Bourget-du-Lac, France; 5 HYDRECO, Laboratoire Environnement de Petit Saut, B.P 823, F-97388 Kourou Cedex, French Guiana

**Keywords:** Community ecology, Conservation biology, Ecological modelling, Tropical ecology

## Abstract

Assessing the impact of human activity on ecosystems often links local biodiversity to disturbances measured within the same locality. However, remote disturbances may also affect local biodiversity. Here, we used environmental DNA metabarcoding to evaluate the relationships between vertebrate biodiversity (fish and mammals) and disturbance intensity in two Amazonian rivers. Measurements of anthropic disturbance -here forest cover losses- were made from the immediate vicinity of the biodiversity sampling sites to up to 90 km upstream. The findings suggest that anthropization had a spatially extended impact on biodiversity. Forest cover losses of <11% in areas up to 30 km upstream from the biodiversity sampling sites were linked to reductions of >22% in taxonomic and functional richness of both terrestrial and aquatic fauna. This underscores the vulnerability of Amazonian biodiversity even to low anthropization levels. The similar responses of aquatic and terrestrial fauna to remote disturbances indicate the need for cross-ecosystem conservation plans that consider the spatially extended effects of anthropization.

## Abstract (web summary)

It is unclear how far the impact of deforestation can spread. Here the authors analyse freshwater eDNA data along two rivers in the Amazon forest, and find that low levels of deforestation are linked to substantial reductions of fish and mammalian diversity downstream.

## Introduction

The current decline in global biodiversity must be addressed proactively to protect and restore ecosystems^1^. A coalition of environmental organizations proposed the protection of at least 30% of the Earth’s surface by 2030 with a final target of 50% by 2050^2,3^. These targets were based on the conservation of the spatial ranges of 85% of all species. However, they did not consider how human disturbances in surrounding unprotected areas will affect the biodiversity of the protected areas.

Given the hydrologic connectivity of river networks, the fauna inhabiting river catchments may be affected by remote disturbances via the water-mediated downstream transfer of matter, energy, and/or organisms^4^. Catchment-scale variables that affect the local conditions of rivers through hydrologic connectivity have been acknowledged^4–8^, as well as the downstream impact of large dams, cities, and land use alterations on river ecology^9,10^. Theoretical efforts to account for the impact of hydrologic connectivity on freshwater conservation planning have been conducted^11^. However, such plans have been poorly implemented due to a lack of theory and tools to resolve this in a systematic conservation planning framework^12^, in part because the spatial extent of the influence of upstream disturbances on downstream biodiversity has not been addressed.

Moreover, the spatial extent of disturbances must be considered both for aquatic and terrestrial fauna as ecological land/water linkages result in lateral connectivity between rivers and terrestrial habitats. Disturbances mediated by hydrologic connectivity may hamper the movement of terrestrial species, the recolonization of defaunated areas, seed dispersal, and pollination^13,14^. The importance of cross-realm connections between adjacent aquatic and terrestrial systems was recently underscored by Leal et al. in 2020^13^, showing that freshwater-focused conservation programmes also benefit nearby terrestrial ecosystems. Therefore, determining whether aquatic and terrestrial fauna respond comparably to disturbances may foster the design and implementation of spatially explicit conservation plans that integrate cross-ecosystem ecological connections^15^. Similar downstream extents of disturbances between aquatic and terrestrial fauna could promote the design of efficient cross-ecosystems conservation programmes^13^. In contrast, different responses of freshwater and terrestrial organisms to remote upstream disturbances would necessitate independent and separate conservation designs.

**Fig. 1:**
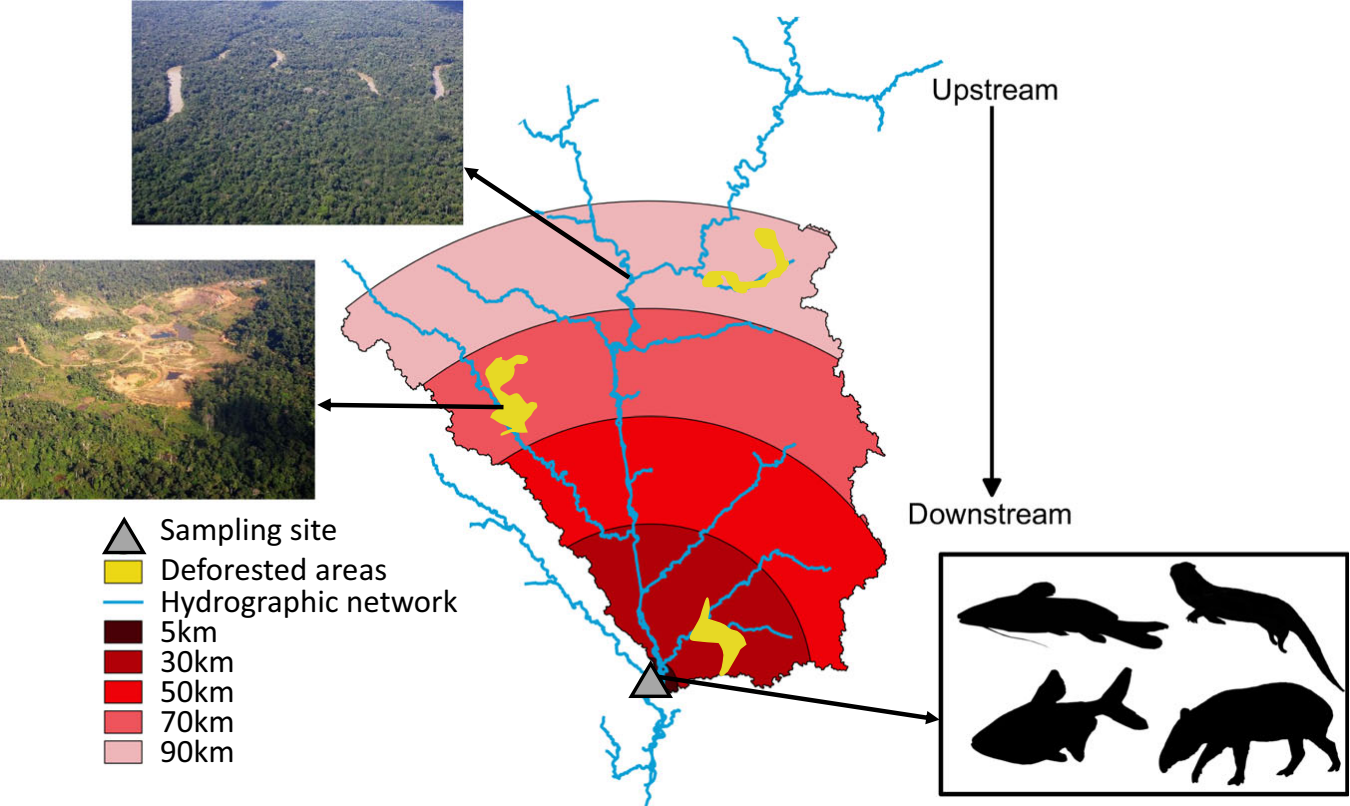
Measurement of the percentage of deforestation upstream from each biodiversity sampling site. For clarity, only five of the 14 spatial extents are illustrated here. Spatial extents are represented by the surface area of the river drainage basin between the biodiversity sampling site and 5, 30, 50, 70, and 90 km upstream from the site. River basin boundaries are indicated by black continuous lines. For each site, the percentage of deforested area for each spatial extent was calculated. For instance, a 30 km extent of disturbance measures the percentage of deforested area within the river basin from the biodiversity sampling site to a maximal distance of 30 km upstream from this site.

To tackle the foregoing issues, we here measured the spatial extent and the strength of anthropization on biodiversity by analysing the relationships between the diversity of local fauna and disturbance intensity measured at multiple scales. We calculated the disturbance intensities at different spatial extents from the immediate vicinity of the biodiversity sampling sites (0.5 km) to a radius of 90 km upstream from the sites (Fig. 1). For each spatial extent, the disturbance intensity was represented by the percentage of deforested surfaces, which served as an integrative measure of anthropization consisting of logging, mining, urban settlements, and conversion of land for agricultural uses^16,17^. On the basis of the hydrologic connectivity of the river systems, we expected that deforestation would have far-reaching effects on the downstream freshwater biodiversity because it modifies water flow, sediment transport, water quality, and aquatic food webs^18–20^. Moreover, the water-mediated transfer of disturbances caused by deforestation could also alter the linkages between aquatic and terrestrial ecosystems and, by extension, affect the terrestrial fauna.

**Fig. 2:**
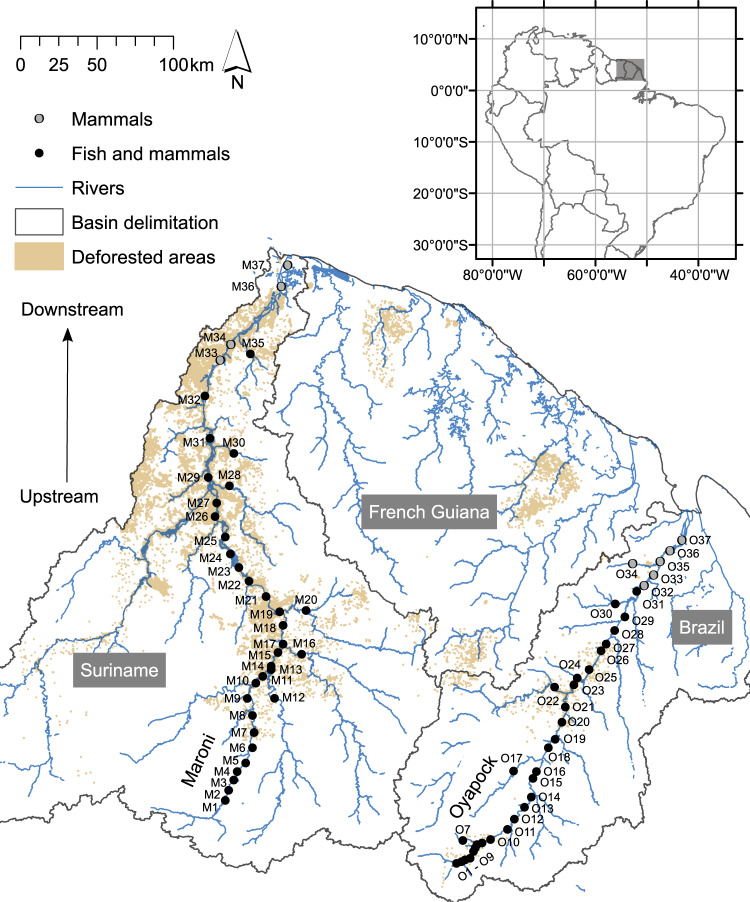
Map of the study area and biodiversity sampling sites. The 64 fish sampling sites are shown in black. Mammals were sampled at all 74 sites. The highlighted grey area in the inset at the upper right indicates the study area in South America.

We applied the foregoing to the Maroni and Oyapock Rivers (Fig. 2) in the Northern Amazonian region (Guiana Shield). Their watersheds have undergone slight anthropization; 0.67% of the basins of both rivers have deforested surfaces. These two rivers are situated in one of the most ecologically intact areas worldwide^17,21^. Nevertheless, they are under unprecedented threat as they are being subjected to increasing deforestation for gold mining. This anthropic activity disperses pollutants and sediments in the rivers and severely disturbs aquatic and terrestrial fauna^22,23^. The recent development of aquatic environmental DNA (eDNA)^24,25^ has enabled us to build simultaneous inventories of aquatic and terrestrial vertebrate fauna along the main river channels and tributaries of the two rivers. We then evaluated the extent to which upstream disturbances are linked to fish and mammal species downstream. As freshwater conservation schemes may also benefit adjacent terrestrial ecosystems^13^, we applied the same framework to both terrestrial and semi-aquatic mammals and determined whether the spatial extent of freshwater disturbances also applies to terrestrial ecosystems.

Here we found that forest cover losses of <11% in areas up to 30 km upstream from the biodiversity sampling sites are linked to reductions of >22% in the taxonomic and functional richness of both terrestrial and aquatic fauna. The absence of some mammal predators and fish detritivores and herbivores downstream from deforested areas suggests that slight deforestation due to mining, logging and agriculture causes remote decreases in biodiversity of downstream fauna, which could impact terrestrial and aquatic ecosystem functioning in the Northern Amazonian region. Moreover, the consistent response of terrestrial and aquatic fauna to upstream deforestation highlights the importance of cross-ecosystem conservation actions accounting for local and distant impacts of disturbances and for cross-ecosystem connectivity.

## Results

**Fig. 3:**
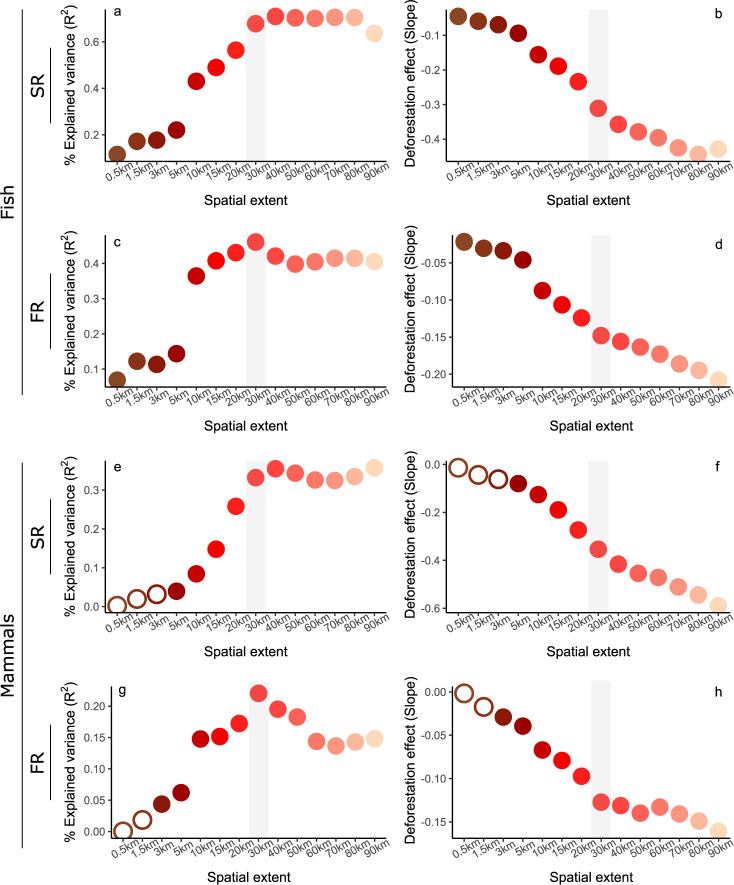
Identification of the relevant spatial extent to measure deforestation impact on biodiversity. Left panels indicate variance explained by mixed models (R^2^) for each spatial extent. Right panels indicate the strength of deforestation effect on biodiversity (slopes). Spatial extents account for deforested areas upstream from eDNA sampling sites. **a**, **b** Fish species richness (SR) models. **c**, **d** Fish functional richness (FR) models. **e**, **f** Mammal species richness models. **g**, **h** Mammal functional richness models. For each spatial extent, a specific generalized linear mixed model accounting for site network position and basin identity as random effects was built for each biodiversity facet. Significant (*p* < 0.05) and non-significant (*p* > 0.05) models assessed with Wald’s tests are indicated by filled and open circles, respectively (Supplementary Table 1). Fish: *n* = 64 sites and mammals: *n* = 74. Colour shades are consistent with spatial extents. Grey vertical bars indicate models with highest R^2^ or R^2^ reaching a plateau, with less than 5% variation between successive spatial extents.

The relationship between biodiversity loss and percentage of deforestation was strongest when upstream deforestation was evaluated over an upstream extent of ≥30 km from the biodiversity sampling sites even under low deforestation intensities (Fig. 3a, c, e, g; see Supplementary Table 1 for *p*-values, slopes and R^2^ values). Indeed, the variance explained by the linear mixed models assessing the links between upstream deforestation and local species and functional richness increased with spatial extent for both fish and mammal species. This was paired with an increase in the negative link between deforestation and both fish and mammals taxonomic (Fig. 3b, f; Supplementary Table 1) and functional diversity (Fig. 3d, h; Supplementary Table 1).

For both taxa, the models were significant for most of the spatial extents considered in our study (Fig. 3). The models considering upstream deforestation within 30 km of the sampling sites were highly significant (*p* < 0.001) and explained >22% of the model variance (Fig. 3, Supplementary Table 1, see Supplementary Fig. 1 for model validity assessment based on residual distributions).

**Fig. 4:**
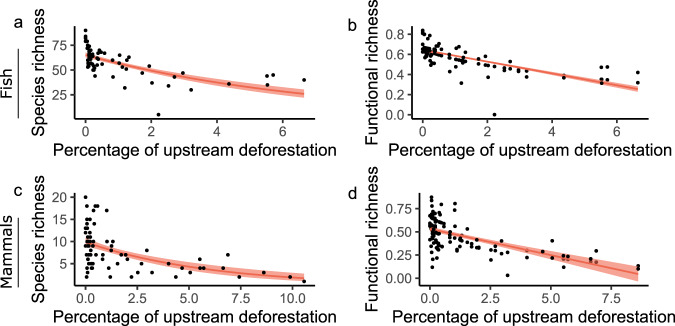
Effects of upstream deforestation intensity on biodiversity. Panels represent species richness (**a**, **c**) and functional richness (**b**, **d**) of fish (**a**, **b**) and mammal (**c**, **d**) communities. Red solid lines indicate the fitted values of mixed models accounting for site network position and basin identity. Light red shades indicate 95% confidence intervals. Deforestation corresponds to the percentage of deforested areas at the most relevant spatial extent (30 km). *n* = 64 sites and *n* = 74 sites for fish and mammal models, respectively.

The models correlating biodiversity with the percentages of deforestation measured within 30 km upstream from the fish and mammal sampling sites provided the best prediction of functional and species richness as the variance they explained stabilized or reached a maximum (Fig. 3a, c, e, g; Supplementary Table 1). At this spatial extent, the deforestation intensities were in the ranges of 0–6.6% and 0–10.6% for the fish and mammal sites, respectively (Supplementary Table 1a, b; Supplementary Fig. 2). Along the deforestation gradients, species and functional richness markedly decreased for fish (Fig. 4a, b) and mammal communities (Fig. 4c, d) despite marked site-specific variability at low deforestation intensity. A comparison of deforested and non-deforested sites (deforestation intensity <0.33%, see methods) revealed significant lower taxonomic and functional diversity at the deforested sites (Kruskal–Wallis test; χ^2^ = 18.4 and *p* < 0.01 for fish species richness; χ^2^ = 11.7 and *p* < 0.01 for mammal species richness; χ^2^ = 13.8 and *p* < 0.01 for fish functional richness; and χ^2^ = 9.1 and *p* < 0.01 for mammal functional richness). On average, there was 34% less species richness (26% and 41% for fish and mammals, respectively) and 28% less functional richness (22% and 33% for fish and mammals, respectively) in deforested sites than in non-deforested ones. This corresponded to average losses of 13 and 4 species for fish and mammal communities, respectively, in deforested sites.

**Fig. 5:**
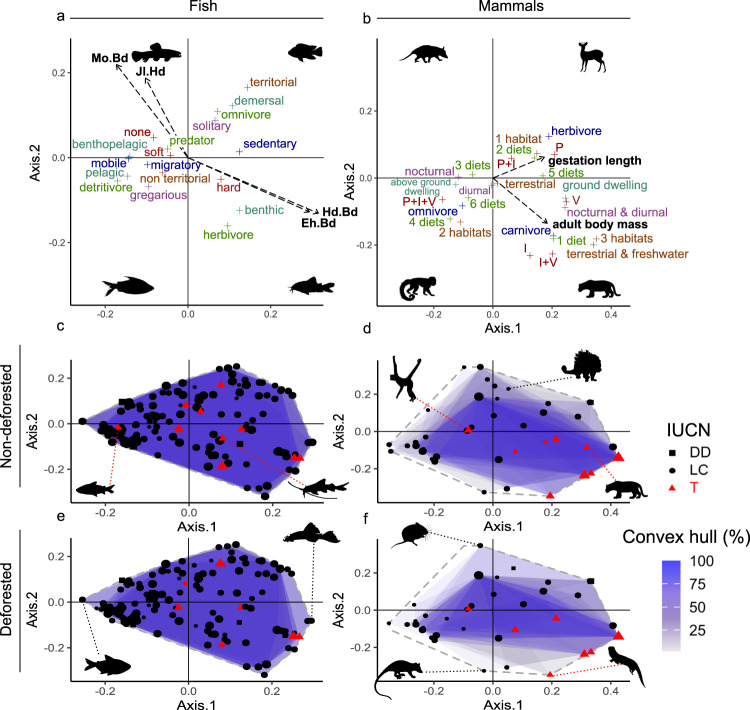
Functional spaces for fish and mammals in non-deforested and deforested sites. Loadings of functional traits on two first axes of principal coordinate analysis for fish (**a**) and mammals (**b**). Only significant and most highly correlated quantitative traits with axes are represented with black dotted lines. Qualitative traits are displayed in colour. For each site, the functional space of each community is represented by a convex hull. Convex hulls of each site were superimposed for each category (fish non-deforested, 34 sites; mammals non-deforested, 35 sites; fish deforested, 30 sites; mammals deforested, 39 sites). Blue shade intensity increases with the percentage of superimposed convex hulls from 0% (white) to 100% (dark blue). The Grey dotted line represents global functional space considering all species in both non-deforested and deforested habitats. Species symbol size is proportional to species occurrence percentages. In IUCN lists, DD is Data-Deficient species; LC is Least Concern species, and T is Threatened species. Sites were considered non-deforested for percentage deforestation <0.33% (see methods) within the identified relevant spatial extent (30 km). Silhouettes illustrate functional characteristics of species in each quadrant (**a**, **b**), emblematic species only found in non-deforested sites (or rarely occurring in deforested sites for spider monkey) (**c**, **d**), and extreme functional strategies (**e**, **f**). See Supplementary Data 1 for species occurrences and coordinates in functional spaces. For **b**, P (Plants in the diet), I (Invertebrates in the diet), V (Vertebrates in the diet), diet (Number of dietary categories), habitat (Number of habitat layers) (Supplementary Table 4).

The fish's functional structure was represented by the two first axes of a principal coordinate analysis (PCoA) of the life history and morphological traits of the species. The first and second axes of the PCoA explained 37% and 24% of the variance and reflected locomotion and swimming/feeding strategies, respectively (Fig. 5a; Supplementary Table 2). In most non-deforested sites, the functional richness of the fish communities (the size of community convex hulls) was high but relatively lower in the deforested sites. The lower functional richness at the deforested sites was the result of the absence of small species with extreme feeding strategies and located on the edge of the functional space (Fig. 5c, e). These species included detritivores and invertivores such as *Moenkhausia* sp. and *Cyphocharax* sp. as well as benthic herbivores of the Loricariidae (Fig. 5e; Supplementary Data 1a). Moreover, certain endangered species such as *Cyphocharax punctatus* and *Pimelodella procera* were never detected in deforested sites (Fig. 5c, e; Supplementary Data 1a).

The mammal functional structure was represented by the two first axes of a PCoA. Axis 1, explaining 38% of the variance, displayed positive gradients of body mass and gestation time, while axis 2, explaining 26% of the variance, accounted for differences in diet and habitat (Fig. 5b; Supplementary Table 2). Marked declines in the most extreme functional strategies were observed for the mammal communities at the deforested sites (Fig. 5d, f) including terrestrial (small rodents and large predators), semi-aquatic (water opossum, *Chironectes minimus*), and aquatic (giant otter, *Pteronura brasiliensis*) species (Supplementary Data 1b). As for fish, the lower mammal functional richness observed in most deforested sites was the result of the absence of certain threatened and emblematic species, such as the spider monkey (*Ateles paniscus*) and the jaguar (*Panthera onca*) (Fig. 5d, f; Supplementary Data 1b).

## Discussion

The present study suggests that slight anthropogenic disturbances can cause remote decreases in the biodiversity of downstream faunas. Forest cover losses of <11% of the total area located up to 30 km upstream from the biodiversity sampling sites were significantly linked to declines of >22% in the taxonomic and functional richness of terrestrial and aquatic fauna, suggesting that anthropization had a cumulative effect on biodiversity over large spatial extents. The link between deforestation and biodiversity reached a maximum when deforestation was measured up to 30 km upstream from the sampling sites, accounting for small deforested surfaces scattered over the upstream areas. The foregoing findings suggest that previous studies linking anthropization to biodiversity without considering the effects mediated by hydrologic connectivity might have overlooked the impact of anthropization on fauna. For instance, studies measuring the local effects of gold mining and forestry-induced deforestation on Guianese fish diversity failed to detect the decline in fish species diversity associated with these activities^26,27^. We obtained similar results when we only considered deforestation over small spatial extents. Therefore, we assume that strong impacts of anthropization would have been detected in the previous studies if larger spatial extents were considered. Moreover, deforestation in the Northern Amazon region and the Guiana Shield is mainly the consequence of small-scale gold mining that reduces water quality through pollutant releases and increases suspended sediment load^18,22^. Gold mining was responsible for about 40% of the deforested surfaces in the area (Supplementary Table 3), while other disturbances such as slash-and-burn agriculture and logging were spatially correlated with gold mining activity (Supplementary Table 3). Thus, mining, agriculture, and logging act together to alter the water quality and physical structure of the rivers in the region^7^, which has negative local effects on fish^26,28^. According to our results, downstream dispersal of these disturbances can also markedly influence fish species over long distances. Those results also hold when considering only terrestrial mammals (Supplementary Fig. 3, Supplementary Note 1). The spatially extended effects of anthropization on terrestrial mammal diversity might be explained by the upstream degradation of riparian vegetation. Through hydrologic connectivity, anthropization affects the structural dynamics of water flow and physico-chemistry, as well as aquatic habitats at the catchment scale^29^. Alterations in freshwater ecosystems may, in turn, affect the downstream structure and composition of the riparian vegetation and the terrestrial biodiversity it supports^29^. Moreover, human settlements established in remote areas in response to the development of small-scale gold mining lead to subsistence hunting along the rivers. This practice also constitutes a non-negligible disturbance to terrestrial fauna^30^.

The biodiversity modifications reported here point out a severe negative impact of anthropization on local biodiversity. When comparing deforested and non-deforested sites, we observed average rates of spatial decline in mammal and fish species richness of 26% and 41%, respectively. This accounts for an average loss of 13 fish and 4 mammal species in anthropized sites, compared to non-anthropized sites. Consequently, the upstream-downstream positive gradient in fish species richness was not verified for the two river basins studied here. This almost universal pattern reflects the roles of fish dispersal and spatial heterogeneity of habitats on fish diversity along river networks^31–33^. The loss of species at the anthropized sites, mainly in the downstream parts of the rivers, reversed the expected distribution pattern of fish diversity, resulting in a higher species richness upstream than downstream. This trend was obvious for the Maroni River (Supplementary Fig. 4), which had undergone more severe anthropization than the Oyapock River. Those alterations in the upstream-downstream species richness gradient were paired with functional alterations linked to the absence of some detritivores and algae browsing fishes in anthropized sites. These species play vital roles in aquatic ecosystems by controlling algal biomass and nutrient cycles^34,35^. Concerning mammals, the absence of the largest and smallest mammal species at most of the anthropized sites parallel the trends reported elsewhere in the world^36,37^. The absence of top predators such as jaguars, giant otters, and bush dogs (*Speothos venaticus*) in anthropized sites could disturb top-down trophic chain control^38^ and thus can deeply impact terrestrial and aquatic ecosystems functioning^39^. Consequently, the ecological roles performed and the ecological services provided by these taxa are jeopardized.

The remote impacts of even slight anthropization on biodiversity suggested by our results require that future conservation plans account for the hydrologic dispersal of human disturbances. This reckoning is of paramount importance even for the slightly affected forest ecosystems that support much of the global terrestrial biodiversity^17^. These wilderness areas continue to shrink and are being fragmented by small and scattered deforestation patches^17,40^. Though these ecosystems apparently remain intact, studies have indicated that they suffer from “silent” human impacts such as hunting of large mammals which reduces animal biodiversity as well as their key roles in seed dispersal and food web control^17,30^. This situation is probably similar for aquatic ecosystems as well because the control that fish exerts on nutrient fluxes and food webs is a strong determinant of ecosystem functioning^35,39^. These “silent” effects could explain the disproportionate biodiversity loss observed in low deforested areas^41^. Here, we suggest that, besides hunting and fishing, distant deforestation also has a “silent” effect on ecosystems by reducing species richness and the functional range of the surviving species. Hydrologically mediated effects must therefore be factored into the design and execution of terrestrial and freshwater conservation plans. For instance, the IUCN could implement the foregoing information to define protected areas and categories that do not consider hydrologically mediated effects^42^. Indeed, the most protected areas (IUCN categories Ia and Ib) are virtually free of human activity but may nonetheless suffer from hydrologically mediated effects caused by alterations remote from the conservation zones. Considering distant disturbances could lead to a re-evaluation of the wilderness in these protected areas and facilitate the determination of the optimal sizes of the buffer zones around them.

Here, both terrestrial and aquatic fauna were related similarly to deforestation over a spatial extent of 30 km upstream from the sampling sites. Thus, cross-ecosystem conservation designs should be beneficial to both terrestrial and aquatic fauna^13^. Nevertheless, spatially explicit, integrated conservation strategies that account for multiple threats and cross-ecosystem connectivity have seldom been developed (but see ref. ^15^ for marine and freshwater ecosystems). Terrestrial and aquatic biodiversity inventories are usually constructed using different methods. Moreover, they are time-consuming and require the participation of specialists in each organism group^43,44^. However, overreaching biodiversity inventory methods such as eDNA metabarcoding are facilitating biodiversity assessments as well as realistic inventories of both aquatic and terrestrial biodiversity within the same water samples^25^. In fact, a single eDNA sampling session can provide fish species inventories that are equivalent and even more complete than those obtained after several years of net samples^45^. Moreover, the eDNA methods generated mammal species distribution patterns consistent with traditional line-transect samplings over the Guianese territory^44^. Nevertheless, the dissemination of eDNA-based biodiversity inventories remains limited because of uncertainty regarding the detection distance of eDNA that is being transported downstream through the river network. Although measuring the exact detection distance is complex and multifactorial^46^, recent studies suggest a short detection distance of eDNA in slow-flowing rivers. In those studies, eDNA samples provided inventories and spatial patterns comparable to those of local samples using capture and observation methods^44,47^, testifying for local detections of species, which do not exceed a few kilometres. These short detection distances might be explained by the accelerated eDNA degradation at warm water temperatures (26–30 °C)^48^ and the gentle topography of Maroni and Oyapock (average slope 0.04% and 0.05%, respectively), which restrict downstream eDNA transport^49^. The situation is more complex in fast-flowing rivers where eDNA may be transported far downstream^49^. However, recently developed spatial eDNA drift modelling can now discriminate local and regional effects^50^. The spread of eDNA technology will therefore constitute an asset to determining the spatial extent and strength of remote human disturbances on biodiversity. Although we here show a consistent response of terrestrial and aquatic faunas, enabling thereby cross-ecosystems conservation actions^13^, our framework deserves to be applied in other regions and to other disturbances. It will contribute to designing efficient conservation plans considering both local and distant impacts of disturbances on the aquatic and terrestrial biodiversity associated with riverine ecosystems.

## Methods

### Study area

The study was conducted on two rivers in north-eastern Amazonia *sensu lato*, including the Guiana Shield and the Amazon River drainage (Fig. 2). The climate of the entire study area is homogeneous and the region is covered by dense, uniform lowland primary rainforest^51^. The altitude is in the range of 0–860 m a.s.l. The regional climate is equatorial, and the annual rainfall ranges from 3600 mm in the northeast to 2000 mm in the southwest. The Maroni River is 612 km long from its source to its estuary, and its watershed covers a surface of >68,000 km^2^ in Suriname and French Guiana. The Oyapock River (length, 404 km; area, 26,800 km^2^) is located in the state of Amapa in Brazil and in French Guiana.

The foregoing river basins host nearly 400 freshwater fish species and more than 180 mammal species^52,53^. Most of the mammal species have a large distribution range, covering the entire study area^53^. The fish species have a less homogeneous distribution and a distinct upstream-downstream composition gradient^54,55^. Here, only large rivers were considered and most fish species were widespread over the whole study area. As habitat availability increases with river size, species richness is expected to increase upstream to dowsntream^31,32^. The Oyapock and Maroni river basins are among the last remaining wilderness areas on Earth^17^. Nevertheless, ecological disturbances are increasing there because of a growing human population and the development of small-scale gold mining activity. These disturbances have caused limited but diffuse deforestation^23,56^. The deforested areas currently comprise 0.67% of all Maroni and Oyapock catchments.

### Sampling

Environmental DNA (eDNA) was collected from water samples at 74 locations (hereafter, sites) along the main channel and the large tributaries of the Maroni and Oyapock rivers (Fig. 2). Thirty-seven sites were sampled at each river basin. The minimum and maximum distances between adjacent sites were 1.07 and 50.20 km, respectively. The mean and median distances between adjacent sites were 10.18 and 9.14 km, respectively, and the standard deviation (SD) was 7.79 km. The sites were located from sea level to 157 m a.s.l. At all sites, the river was wider than 20 m and deeper than 1 m (Strahler orders 4–8; Supplementary Fig. 5). The physicochemical properties of the water slightly varied among sites. The temperature, pH, and conductivity were in the ranges of 28.4–33.2 °C, 6.5–7.6, and 16.9–54.6 µS/cm, respectively, at all sites except two estuarine locations where the conductivity was relatively high because of seawater incursion (Supplementary Data 2).

The eDNA samples were collected during the dry seasons (October–November) of 2017 and 2018 for Maroni and Oyapock, respectively. At both rivers, the sites were sequentially sampled from downstream to upstream at a rate of 1–4 sites per day depending on the distance and travel time between sites. Following the protocol of ref. ^45^, we collected the eDNA by filtering two replicates of 34 L of water per site. A peristaltic pump (Vampire Sampler; Buerkle GmbH, Bad Bellingen, Germany) and single-use tubing were used to pump the water into a single-use filtration capsule (VigiDNA, pore size 0.45 μm; filtration surface 500 cm^2^, SPYGEN, Bourget-du-Lac, France). The tubing input was placed a few centimetres below the water surface in zones with high water flow as recommended by Cilleros et al.^43^. Sampling was performed in turbulent areas with rapid hydromorphologic units to ensure optimal eDNA homogeneity throughout the water column. To avoid eDNA cross-contamination among sites, the operator remained on emerging rocks downstream from the filtration area. At the end of filtration, the capsule was voided, filled with 80 mL CL1 preservation buffer (SPYGEN), and stored in the dark up to one month before the DNA extraction. No permits were required for the eDNA sampling and the access to all sites was legally permitted. The study complies with access and benefit permits ABSCH-IRCC-FR-246820-1 and ABSCH-IRCC-FR-245902-1, authorizing collection, transport and analysis of all environmental DNA samples used in this study.

### Laboratory procedures and bioinformatic analyses

For the DNA extraction, each filtration capsule was agitated on an S50 shaker (Ingenieurbüro CAT M. Zipperer GmbH, Ballrechten-Dottingen, Germany) at 800 rpm for 15 min, decanted into a 50 mL tube, and centrifuged at 15,000 × *g* and 6 °C for 15 min. The supernatant was removed with a sterile pipette, leaving 15 mL of liquid at the bottom of the tube. Subsequently, 33 mL of ethanol and 1.5 mL of 3 M sodium acetate were added to each 50 mL tube, and the mixtures were stored at −20 °C for at least one night. The tubes were then centrifuged at 15,000 × *g* and 6 °C for 15 min, and the supernatants were discarded. Then, 720 µL of ATL buffer from a DNeasy Blood & Tissue Extraction Kit (Qiagen, Hilden, Germany) was added. The tubes were vortexed, and the supernatants were transferred to 2 mL tubes containing 20 µL proteinase K. The tubes were then incubated at 56 °C for 2 h. DNA extraction was performed using a NucleoSpin Soil kit (Macherey-Nagel GmbH, Düren, Germany) starting from step six of the manufacturer’s instructions. Elution was performed by adding 100 µL of SE buffer twice. After the DNA extraction, the samples were tested for inhibition by qPCR following the protocol in ref. ^57^. Briefly, quantitative PCR was performed in duplicate for each sample. If at least one of the replicates showed a different Ct (Cycle threshold) than expected (at least 2 Cts), the sample was considered inhibited and diluted 5-fold before the amplification.

For the fish, the “teleo” primers^58^ (forward: 3ʹ-ACACCGCCCGTCACTCT-5ʹ; reverse: 3ʹ-CTTCCGGTACACTTACCATG-5ʹ) were used as they efficiently discriminated local fish species^43,45^. For the mammals, the 12S-V5 vertebrate marker^59^ (forward: 3ʹ-TAGAACAGGCTCCTCTAG-5ʹ; reverse: 3ʹ-TTAGATACCCCACTATGC-5ʹ) was used as it also effectively distinguishes local mammal species^44,60^. The DNA amplifications were performed in a final volume of 25 μL containing 1 U AmpliTaq Gold DNA Polymerase (Applied Biosystems, Foster City, CA, USA), 0.2 μM of each primer, 10 mM Tris-HCl, 50 mM KCl, 2.5 mM MgCl2, 0.2 mM of each dNTP, and 3 μL DNA template. Human blocking primer was added to the mixture for the “teleo”^58^ (5′-ACCCTCCTCAAGTATACTTCAAAGGAC-C3-3′) and the “12S-V5” primers^61^ (5′-CTATGCTTAGCCCTAAACCTCAACAGTTAAATCAACAAAACTGCT-C3-3′) at final concentrations of 4 μM and 0.2 μg/μL bovine serum albumin (BSA; Roche Diagnostics, Basel, Switzerland). Twelve PCR replicates were performed per field sample. The forward and reverse primer tags were identical within each PCR replicate. The PCR mixture was denatured at 95 °C for 10 min, followed by 50 cycles of 30 s at 95 °C, 30 s at 55 °C for the “teleo” primers and 50 °C for the 12S-V5 primers, 1 min at 72 °C, and a final elongation step at 72 °C for 7 min. This step was conducted in a dedicated room for DNA amplification that is kept under negative air pressure and is physically separated from the DNA extraction rooms maintained under positive air pressure. The purified PCR products were pooled in equal volumes to achieve an expected sequencing depth of 500,000 reads per sample before DNA library preparation.

For the fish analyses, 10 libraries were prepared using a PCR-free library protocol (https://www.fasteris.com/metafast) at Fasteris, Geneva, Switzerland. Four libraries were sequenced on an Illumina HiSeq 2500 (2 × 125 bp) (Illumina, San Diego, CA, USA) with a HiSeq SBS Kit v4 (Illumina), three were sequenced on a MiSeq (2 × 125 bp) (Illumina) with a MiSeq Flow Cell Kit Version3 (Illumina), and three libraries were sequenced on a NextSeq (2 × 150 bp + 8) (Illumina) with a NextSeq Mid kit (Illumina). The libraries run on the NextSeq were equally distributed in four lanes. Sequencing was performed according to the manufacturer’s instructions at Fasteris. For the mammal analyses, eight libraries were prepared using a PCR-free library protocol (https://www.fasteris.com/metafast) at Fasteris. Two libraries were sequenced on an Illumina HiSeq 2500 (2 × 125 bp) (Illumina) using a HiSeq Rapid Flow Cell v2 and a HiSeq Rapid SBS Kit v2 (Illumina), three libraries were prepared on a MiSeq (2 × 125 bp) (Illumina) with a MiSeq Flow Cell Kit Version3 (Illumina), and three libraries were prepared using a NextSeq (2 × 150 bp + 8) (Illumina) and a NextSeq Mid kit (Illumina). The libraries run on the NextSeq were equally distributed in four lanes. As different sequencing platforms were used (MiSeq and NextSeq for the Maroni and HiSeq 2500 and MiSeq for the Oyapock; Supplementary Fig. 6 and Supplementary Data 3), the possible influences of the platforms on the sequencing results were verified. To this end, we compared the differences in species numbers between the sample replicates assigned to the same platform (accounting for replicate effect only) against those of the sample replicates assigned to different platforms (accounting for replicate and platform effects). As there were more sites with their two replicates sequenced with the same platform than sites with their replicates sequenced with different platforms (see Supplementary Fig. 6), sites with replicates on the same platform were randomly selected for the comparisons. We repeated this procedure 50 times. The number of species between replicates sequenced on the same platform and those sequenced on different platforms did not differ for >98.5% of all fish and mammal samples (Supplementary Fig. 7 and Supplementary Note 2). Similar to these results, a previous study on 16 S rRNA amplicon has shown that the samples were not influenced by the Illumina sequencing platform used^62^.

To monitor for contaminants, 13 negative extraction controls were performed for each of the primers (“teleo” and “12S-V5”); one control was amplified twice. All of them were amplified and sequenced by the same methods as the samples and in parallel to them. Therefore, for the negative extraction controls, 168 amplifications were prepared with the “teleo” primers (13 negative controls; one amplified and sequenced twice) and 156 amplifications with the “12S-V5” primers (13 negative controls). Fourteen negative PCR controls (ultrapure water; 12 replicates) were amplified and sequenced in parallel to the samples. Eight were amplified with the “teleo” primers and six were amplified with the “12S-V05” primers. Thus, for the PCR negative controls, there were 96 amplifications with the “teleo” primers and 72 amplifications with the Vert01 primers. Sequencing information for the controls is shown in Supplementary Data 3c.

An updated version of the reference database from ref. ^43^ was used. There were 265 Guianese species for the fish analyses (ref. ^47^). The GenBank nucleotide database was consulted, but it contained little information on the Guianese fish species. Most of the sequences were derived from ref. ^43^. For the mammal analyses, the vertebrate database was built using ecoPCR software^63^ from the releases 134 and 138 of the European Nucleotide Archive (ENA), for the Maroni and Oyapock river samples, respectively. The two releases were compared, and it was established that the new mammal species added to each version did not originate from French Guiana. Hence, the results were not influenced by the EMBL release number. The relevant metabarcoding fragment was extracted from this database with ecoPCR^63^ and OBITools^64^. Therefore, the reference database comprised the local database of French Guianese mammals^60^, which references 576 specimens from 164 species as well as all available vertebrate species in EMBL.

The sequence reads were analyzed with the OBITools package according to the protocol described by Valentini et al.^58^. Briefly, the forward and reverse reads were assembled with the illuminapairedend programme. The ngsfilter programme was then used to assign the sequences to each sample. A separate dataset was created for each sample by splitting the original dataset into several files with obisplit. Sequences shorter than 20 bp or occurring less than 10 times per sample were discarded. The obiclean program was used to identify amplicon sequence variants (ASVs) that have likely arisen due to PCR or sequencing errors. It uses the information of sequence counts and sequence similarities to classify whether a sequence is a variant (“internal”) of a more abundant (“head”) ASV^64^. After this step, we matched the ASV with the reference database to obtain the taxonomic assignation for each ASV. Sequences labelled by the obiclean programme as ‘internal’’ and probably corresponding to PCR errors were discarded. The ecotag programme was then used for taxonomic assignment of molecular operational taxonomic units (MOTUs). The taxonomic assignments from ecotag were corrected to avoid overconfidence in assignments. Species-level assignments were validated only for ≥98% sequence identity with the reference database. Sequences below this threshold were discarded.

### Measuring disturbance intensity using GIS data

In riverine systems, the disturbances may accumulate because of hydrologic connectivity, which is the downstream transfer of matter and pollutants^4^. Hence, the upstream sub-basin drainage network was considered to determine the size of the upstream sub-basin affecting local biodiversity (Fig. 1). The sub-basins were delineated by applying a flow accumulation algorithm to the SRTM global 30 m digital elevation model^65^. Deforestation was measured over 14 upstream spatial extents with radii of 0.5, 1.5, 3, 5, 10, 15, 20, 30, 40, 50, 60, 70, 80, and 90 km for each sampling site. Then, these 14 upstream spatial extents were intersected with the sub-basin drainage network. In addition, mammals and fish can also be affected by disturbances other than those mediated by hydrologic connectivity. Thus, deforestation was also measured upstream and downstream from the eDNA sampling sites using the same foregoing 14 radii.

At each sampling site, deforestation intensity was quantified for each of the 14 spatial extents. We summed upstream (only accounting for disturbances mediated by river hydrologic connectivity) or upstream and downstream (not only considering disturbances mediated by hydrologic connectivity) deforested surfaces from Landsat satellite image datasets. Forest loss surfaces were obtained from the Global Forest Change dataset^66^. The Global Forest Change dataset identifies areas deforested between 2001 and 2017 on a 30 m spatial scale. To incorporate deforested areas prior to 2000, tree canopy cover data for that year were also used. Except for river courses, all pixels with <25% canopy closure were regarded as deforested. Finally, surfaces deforested by gold mining activity in French Guiana, Suriname, and Northern Brazil were also included^56,67^.

Forest loss and gold-mined surfaces were significantly positively correlated for each spatial extent (Supplementary Table 3). We thus merged those datasets to create an integrative disturbance variable that quantifies the deforestation around the sampling sites, for each spatial extent. Here, deforestation intensity around each eDNA sampling site was considered an integrative measure of human-mediated environmental disturbances called here anthropization, which includes gold mining, logging, agriculture, and human settlements (Supplementary Table 3).

The absolute deforested surfaces are dependent on the surface area measured at each spatial extent, making the absolute value of deforestation dependent on the spatial extent considered. Similarly, within each spatial extent, the area of the considered upstream river basin varies with the shape of the river, making again the absolute deforestation surface dependent on the area considered. For this reason, deforestation was calculated as a percentage of the absolute deforested surface area divided by the surface area considered instead of an absolute deforested surface. We nevertheless ran separated models (see the Species and functional richness models section for details on model structure) using the percentage, the absolute measures of deforestation and the scaled absolute measures of deforestation. Absolute deforestation did not provide informative results (Supplementary Fig. 8) because it depends on the surface considered. Using the scaled absolute measures of deforestation (Supplementary Fig. 9) increased the proportion of variance explained by the models but it remained lower than the explained variance obtained with percentages of deforested areas. Additionally, The assessment of biodiversity responses to deforestation percentages measured upstream and downstream from the eDNA sampling disclosed only weak or non-significant relationships between deforestation and biodiversity (Supplementary Fig. 10, see the *Species and functional richness models* section for details on model structure). For instance, the models that yielded significant (*p* < 0.05) results with upstream and downstream deforestation as an explicative variable explained only <6% of the variance for both fish and mammals (Supplementary Fig. 10). Hence, we used the percentage of upstream deforestation as a measure of anthropization for the main analyses.

Based on the biodiversity sampling sites, the upstream deforestation intensity was, on average, <5% for all spatial extents considered (Supplementary Fig. 2; Supplementary Table 1). At reduced spatial extents (0.5–10 km), deforestation was in the range of 0–39.21% (median, 0.54%). To larger extents, however, the deforestation intensity was in the range of 0–16.38% (median, 0.33%) (Supplementary Fig. 2; Supplementary Table 1). The intensity and the variability of upstream deforestation thus decreased with increasing spatial extents. All the spatial analyses were performed on ArcGIS 10.8.

### Biodiversity measures

The collected fish and mammal eDNA was amplified to build species inventories (see Supplementary Data 4 for the numbers of reads and detected species per sample). For the freshwater fish communities, 64 strictly freshwater sites were regarded (Fig. 2). Estuarine areas were not considered for fish because the molecular reference database did not support the detection of marine or estuarine fish species. Detecting more than 70% of the site's expected fish fauna in another study, the sampling protocol used here was shown to provide similar or more complete inventories to those derived from gill-netting in other large rivers within the study region^45^. Moreover, recent work on the same rivers showed that eDNA describes local fish communities and generates a spatial signal comparable to that of capture-based methods describing fish species over a few hundred metres^47^. Mammal communities were considered for all 74 sites (Fig. 2).

The collected DNA supported the detection of 158 fish species with 5–90 (mean 58 ± 1.9 SE) species per site and 46 mammal species with 1–20 (mean 8 ± 0.54 SE) species per site. Twenty-two species (9 mammals and 13 fish) are classified as Threatened according to the IUCN^68^. Mammal species with limited or poorly known distributions such as the West Indian manatee and Chiroptera were excluded. All mammals detected in the present study including five semi-aquatic, 15 terrestrial, and 26 arboreal species were reliably inventoried within the same study area by the sampling method used here^44^.

The biodiversity of each taxon at each site was measured via species and functional richness. Species richness was the number of species detected from two eDNA samples collected at each site. This sampling effort has been shown to provide relevant local fish and mammal inventories^44,45^. Functional diversity was measured using morphological and ecological traits available from the literature. Supplementary Fig. 4 shows the spatial patterns of the fish and mammal species and functional richness along the upstream-downstream gradients of the two rivers studied here.

Both the morphological and ecological traits of the fish were used as they complement functional diversity measurements for freshwater fish^69^. For the morphological traits, 12 measurements were made using side-view pictures collected over the past decade to compute 10 unitless ratios (hereafter, traits) reflecting food acquisition and locomotion^69,70^ (Supplementary Table 4a). The morphological traits were measured for as many individuals as possible (1–20 depending on the species) and the averages of all measurements per species were used. Intraspecific variability in morphological traits was not considered because a recent study using the same dataset demonstrated that it was negligible^70^. The maximum body length of each species obtained from FishBase (www.fishbase.org) represented the maximum body size for the species and was regarded as a synthetic functional trait^69^. Therefore, 11 continuous traits were used to characterize fish morphological diversity. For the ecological traits, six qualitative traits related to trophy, behaviour, and habitat preference were selected (Supplementary Table 4a) and collected from FishBase (www.fishbase.org) and the literature^54,55^.

For the mammals, the morphological traits were compiled from different databases to maximize the number of traits and minimize the missing values (Supplementary Table 4b). Longevity, gestation length, litter or clutch size, and adult body mass were selected from the Amniote database^71^. Activity cycle, habitat and diet breadth, trophic level, and terrestriality were taken from the Pantheria database^72^. Type of habitat (re-categorized from terrestrial, marine, freshwater, and aerial binary variables) and diet (re-categorized from proportions of vertebrates, invertebrates, and plants in the diet) was derived from the Phylacine database^73^ (Supplementary Table 4b).

The morphological traits presented a correlation coefficient <0.7 (see Supplementary Fig. 11 for the trait correlograms), and the categorical ecological traits were combined to build functional spaces and assess functional diversity. Gower’s functional distances between species were calculated for each taxon. This parameter considers categorical and continuous traits, standardizes them, and handles missing data. The distance matrices (one per taxon) were ordinated into multidimensional spaces by a principal coordinate analysis (PCoA), which generates coordinates for all species within a global functional space per taxon. To calculate functional richness^74^, the first five PCoA axes for fish and the first two PCoA axes for mammals were retained. This configuration maximized functional space quality^75^ and minimized data loss, as sites must have more species than the number of axes selected to compute functional richness. The resulting measure is the convex hull volume occupied by co-occurring species at each site in the functional space and is in the range of 0–1. Higher values reflect high volume occupation and, therefore, high functional diversity.

### Species and functional richness models

For each spatial extent, a specific model was constructed to analyze the effects of deforestation on species and functional richness. Generalized linear mixed models (GLMM) with Poisson’s distributions were used for species richness as species richness is a count variable. Linear mixed models (LMM) were used for functional richness as this variable is continuous and ranges between 0 and 1. As few sites had high upstream deforestation values and several sites had deforestation values close to 0, upstream deforestation was square-root transformed to down-weight the few high deforestation values. Those models were implemented for each taxon and for each diversity facet. This resulted in 56 models [two taxa × two diversity facets × 14 spatial extents] for the main analyses. River basin identity and site position in the upstream-downstream river network (Strahler order; Supplementary Fig. 5) were included as random effects in the models because site position determines the river size, and therefore, the hosting capacity of aquatic species^31,32^. Basin identity accounts for biogeographical processes shaping diversity. The models were built using the *lmer* function in the lme4 package of R.

The significance and the variance explained per model were calculated using a coefficient of determination (R^2^). The aim was to establish which spatial extent best predicts the relationship between local biodiversity and deforestation intensity. R^2^ was calculated using the *r.squaredGLMM* function in the MuMIn package of R. Marginal R^2^ values, which account only for the variance explained by fixed variables, were used to identify the pure effects of deforestation. The spatial extent associated with the highest R^2^ or stabilization of R^2^ with <5% change in R^2^ between successive spatial extents was taken to be the most relevant spatial extent in the assessment of the effects of deforestation on biodiversity. The slope of the model at the optimal spatial extent was used to evaluate the strength of deforestation. Model validity was assessed by checking the absence of residual patterns and by testing the normal distribution of the residuals with Shapiro tests (Supplementary Fig. 1).

Sampling sites were located along two upstream-downstream gradients and were, therefore, not independent of each other. Spatial autocorrelation was evaluated using Moran's *I* test on the GLMM and LMM residuals for the fish and mammal species and functional richness across all sites to test for unforeseen associations between nearby sites. After accounting for basin identity and position in the watercourse, we determined that species and functional richness of both taxa were not influenced by spatial autocorrelation (Moran’s *I* test; fish species richness, observed = 0.04, *p* = 0.15; fish functional richness, observed = 0, *p* = 0.69; mammal species richness, observed = 0.04, *p* = 0.17; mammal functional richness, observed = 0, *p* = 0.84). Furthermore, the robustness of the findings was tested by performing a sub-sampling analysis on subsets of sites with increasing minimal distance (range: 2–50 km) between sites. For each minimal distance between sites, 50 site subsets were randomly built with the same GLMM and LMM model analyses as those applied for the entire dataset. This site subset analysis yielded results similar to those obtained using the entire dataset (Supplementary Data 5). Hence, the results were robust and were not influenced by the distances between adjacent sites. R code to compute generalized linear mixed models is provided as Supplementary software 1.

### Functional structure analysis

Within the optimal spatial extent, sites were classified by deforestation level into deforested sites (deforested area exceeding 0.33%) and non-deforested sites (deforested area <0.33% explained by natural forest turnover or tree fall). This threshold was determined by measuring natural deforestation at 100 randomly selected sites in areas without human settlement, human activity, or anthropogenic deforestation. Half-circle spatial extents with a radius of 30 km were generated for each site, representing surfaces similar to those of the spatial extents delimited for the sampling sites, and deforestation percentages were then calculated. The highest deforestation percentage was regarded as a threshold of natural deforestation (i.e. natural forest turnover, hereafter called non-deforested sites) and anthropic-mediated deforestation (i.e. deforested sites). Applying this threshold to the sampling sites and considering deforestation over a 30 km upstream spatial extent from the sampling sites, yielded 34 and 35 non-deforested sites and 30 and 39 non-deforested sites for fish and mammals, respectively.

The *envifit* function in the vegan package of R was used to fit the variables (traits) onto the PCoA ordination and identify any correlations between the traits and the ordination axes (Supplementary Table 2). The determination coefficients R^2^ were calculated to assess the strengths of the correlations between the axes and the traits. Traits with high R^2^ were strong ordination predictors. *P*-values were computed by comparing the observed and simulated R^2^ based on 999 random data permutations. To quantify the trait contributions, the continuous variables were transformed onto vectors directed according to their correlation with the axes. Their lengths were proportional to the strengths of the correlations between the ordinations and the traits (R^2^) (Supplementary Table 2b). For the categorical variables, the average ordination scores were computed for the scores of all species belonging to each factor level to locate categories in the functional spaces (Supplementary Table 2c). Convex hulls of each community at each site were represented within the global functional spaces for fish and mammals and for both habitat types. Species were included in the functional spaces and rated Threatened if they were classified as Critically Endangered (CR), Endangered (EN), Vulnerable (VU), or Near Threatened (NT) according to the IUCN Red List^68^. The percentages of species occurrences (number of occurrences of each species divided by the sum of all occurrences of all species, Supplementary Data 1a, b) were calculated and displayed in the functional spaces. R code to compute functional spaces is provided as Supplementary software 1.

### Reporting summary

Further information on research design is available in the Nature Research Reporting Summary linked to this article.

## Supplementary information

Supplementary Information File

Peer Review File

Description of Additional Supplementary Files

Supplementary Data 1

Supplementary Data 2

Supplementary Data 3

Supplementary Data 4

Supplementary Data 5

Supplementary Software 1

Reporting Summary

## Data Availability

The data generated in this study are provided in the Supplementary Information files. It includes Supplementary Figs. 1 to 11, Supplementary Tables 1 to 4 and Supplementary Notes 1 and 2. The Illumina raw sequence data used in this study are available under accession code 10.5061/dryad.pvmcvdnmr for fish samples and 10.6084/m9.figshare.13739086.v6 for mammal samples. The runs used for this study can be extracted using the sequencing information in Supplementary Data 3. The fish maximum body length of each species was extracted from FishBase (www.fishbase.org). Mammal functional traits were obtained from three databases: Phylacine 1.2 (10.1002/ecy.2443), Amniote (10.1890/15-0846R.1) and PanTHERIA (10.1890/08-1494.1). The reference database used for fish was an updated version of ref. ^43^ (10.1111/1755-0998.12900). The reference database used for mammals comprised the local database of French Guianese mammals (10.1111/2041-210X.12729), as well as all available vertebrate species in EMBL (https://www.embl.org). Forest loss surfaces were obtained from the Global Forest Change dataset (10.1126/science.1244693). Surfaces deforested by gold mining activity in French Guiana, Suriname and Northern Brazil were extracted from ref. ^57^.

## Code availability

Code for reproducing the results in this study is available as Supplementary software and also from corresponding authors on reasonable request.
