# Supplementary material for: Low level of anthropization linked to harsh vertebrate biodiversity declines in Amazonia
Source: Nat Commun. 2022 Jun 7;13:3290. doi: 10.1038/s41467-022-30842-2 (PMC9174194; doi:10.1038/s41467-022-30842-2)
Supplement: Supplementary file 1 — Supplementary Information File [file 41467_2022_30842_MOESM1_ESM.pdf]

Supplementary materials

Low level of anthropization linked to harsh vertebrate biodiversity declines in Amazonia

Isabel Cantera[1,2*], Opale Coutant[1*], Céline Jézéquel[1], Jean-Baptiste Decotte[3], Tony Dejean[3,4], Amaia Iribar[1], Régis Vigouroux[5], Alice Valentini[3,4], Jérôme Murienne[1] and Sébastien Brosse[1].

[1]Laboratoire Evolution et Diversité Biologique (UMR 5174), CNRS, IRD, Université Paul Sabatier, 118 route de Narbonne, 31062 Toulouse, France. celine.jezequel@ird.fr, amaya.pelozuelo@univ-tlse3.fr, sebastien.brosse@univ-tlse3.fr

[2]Department of Environmental Science and Policy, Università degli Studi di Milano. Via Celoria 10, 20133 Milano Italy. isabel.cantera@unimi.it

[3]VIGILIFE, 17 rue du Lac Saint-André Savoie Technolac - BP 274, Le Bourget-du-Lac 73375, France. jb.decotte@vigilife.org

[4]SPYGEN, 17 rue du Lac Saint-André Savoie Technolac - BP 274, Le Bourget-du-Lac 73375, France. tony.dejean@spygen.com, alice.valentini@spygen.com

[5]HYDRECO, Laboratoire Environnement de Petit Saut, B.P 823, F-97388, Kourou Cedex, French Guiana. regis.vigouroux@hydrecolab.com

*These authors contributed equally

Corresponding author: opale.coutant@univ-tlse3.fr.

Supplementary material includes:

Supplementary Notes 1 and 2

Supplementary Tables 1 to 4

Supplementary Figures 1 to 11

Supplementary Note 1: Contribution of water-dependent mammals to the variance explained by the models.

We analysed the influence of water-dependent mammals on the explained variance retrieved from the mixed models to understand if water-dependent species drive the observed response to deforestation. We compared model results excluding the five water-dependent mammals (Giant otter (*Pteronura brasiliensis*), Neotropical otter (*Lontra longicaudis*), Water opossum (*Chironectes minimus*), Capybara (*Hydrochoerus hydrochaeris*) and Lowland tapir (*Tapirus terrestris*)) to 999 sets of species combinations in which five non-aquatic mammals were randomly removed. Species and functional richness were calculated for each set and implemented in the 14 mixed models considering deforestation at different spatial extents. Then we calculated the Standardized Effect Size (SES) as $R^2_{\text{withtout water-dependent species}}$ – mean ($R^2_{\text{random})}$ /sd (R^2_{random}), (sd = standard deviation). SES < -2 indicates that water-dependent species contribute significantly more to the explained variance than nonaquatic species. SES > -2 indicates that the contribution of water-dependant species to the explained variance is not significantly different to that of nonaquatic species

For species richness, as shown in Supplementary Figure 3a, c, models remain significant despite a slight decrease of R^2 (0.33 to 0.23 at 30 km) when water-dependent species are removed. Supplementary Figure 3e indicates that for most extents (except 0.5 to 5km), water-dependent species contributed more to the explained variance of the models than the other species. Supplementary Figure 3g demonstrates that regardless of the species removed, models in which upstream deforestation was measured above 5km upstream from the biodiversity sampling sites always remained significant. It thus indicates that even if the five water-dependent species are more affected by upstream deforestation than the other species, the entire mammal community remains affected by upstream deforestation.

For functional richness, explained variance drastically decreased when excluding water-dependent mammals and most of the models were not significant (Supplementary Figure 3b, d). However, Supplementary Figure 3f demonstrates that for most extents (except 10 to 20km), water-dependent species did not have a higher contribution to the explained variance than the other species. This suggests that the decrease of explained variance is rather due to the removal of five species than the removal of the five water-dependent species. This is also supported by Supplementary Figure 3h showing that a substantial part of the p.values is above the significance threshold. Altogether, these panels indicate that there is a set of species with extreme functional strategies (and not restricted to water-dependent species) driving the community response to upstream deforestation.

In conclusion, both water-dependent and terrestrial mammals significantly contribute to the models relating taxonomic and functional biodiversity to deforestation.

Supplementary Note 2: Effect of the sequencing platform on species richness.

To distinguish the replicate effect (variability in the two replicates from the same site due to sampling and site effects) from the sequencing platform effect, we compared the difference in species number between site replicates assigned to the same platform (accounting for the replicate effect only) to the difference in species number between site replicates assigned to different platforms (accounting for replicate and platform effects). A difference in species number of the site replicates assigned to different platforms higher than that of the site replicates assigned to the same platform, would indicate a platform effect.

Sites with replicates on the same platform were randomly selected to be compared to the same number of sites with replicates on different platforms. Then, for each site, we calculated the difference in species number between the two replicates and conducted a Mann-Whitney U test to compare the mean difference in species number between site replicates assigned to the same platform to that of the site replicates assigned to different platforms. We repeated this procedure 50 times and displayed the distribution of p.values in Supplementary Figure 7. Analyses were conducted separately for the Maroni and Oyapock rivers and for the two taxa because different platforms were used.

For the Maroni river, none of the comparisons were significant (p-value > 0.05), for both fish and mammals. For the Oyapock river, none of the comparisons were significant for fish, while 6% of the comparisons were significant for mammals.

We thus conclude that the sequencing platform has a negligible effect on species richness.

TABLES

Supplementary Table 1 Mean, maximum and minimum values of global percentage of upstream deforested surfaces for each spatial extent. Results of the mixed models relating biodiversity variables (species and functional richness) and deforestation for the 14 spatial extents considering upstream deforestation are presented. For each spatial extent, a specific generalized linear mixed model was built. River basin identity and site position in the upstream-downstream river continuum (Strahler order) were included as random effects. The statistical significance of the models was assessed with Wald's test. Significant p-values and most relevant R^2 values (highest value or R^2 value reaching a plateau, with less than 5% variation between successive spatial extents) are indicated in bold. (a) the 64 fish sampling sites and (b) the 74 mammal sampling sites.

a

Spatial extent	***Mean deforestation percentage***	***Minimum deforestation percentage***	***Maximum deforestation percentage***	***Species richness***			***Functional richness***		
				Slope	p-value	R^2	Slope	p-value	R^2
0.5km	4.6	0.0	33.3	-0.05	**<0.01**	0.12	-0.02	**0.02**	0.07
1.5km	4.7	0.0	39.2	-0.06	**<0.01**	0.17	-0.03	**<0.01**	0.12
3km	3.7	0.0	34.3	-0.07	**<0.01**	0.18	-0.03	**<0.01**	0.11
5km	2.6	0.0	26.7	-0.09	**<0.01**	0.22	-0.05	**<0.01**	0.14
10km	2.0	0.0	13.9	-0.16	**<0.01**	0.43	-0.09	**<0.01**	0.36
15km	1.6	0.0	13.3	-0.19	**<0.01**	0.49	-0.11	**<0.01**	0.41
20km	1.2	0.0	9.9	-0.23	**<0.01**	0.56	-0.12	**<0.01**	0.43
30km	1.0	0.0	6.6	-0.31	**<0.01**	**0.68**	-0.15	**<0.01**	**0.46**
40km	0.9	0.0	4.7	-0.36	**<0.01**	0.71	-0.16	**<0.01**	0.42
50km	0.8	0.0	4.3	-0.38	**<0.01**	0.70	-0.16	**<0.01**	0.4
60km	0.8	0.0	4.0	-0.4	**<0.01**	0.70	-0.17	**<0.01**	0.4
70km	0.7	0.0	4.4	-0.43	**<0.01**	0.71	-0.19	**<0.01**	0.41
80km	0.7	0.0	4.4	-0.44	**<0.01**	0.70	-0.19	**<0.01**	0.41
90km	0.6	0.0	4.0	-0.43	**<0.01**	0.64	-0.21	**<0.01**	0.41

b

Spatial extent	***Mean deforestation percentage***	***Minimum deforestation percentage***	***Maximum deforestation percentage***	***Species richness***			***Functional richness***		
				Slope	p-value	R^2	*Slope*	*p-value*	R^2
0.5km	4.3	0.0	33.3	-0.01	0.61	0	0.00	0.88	0.00
1.5km	4.8	0.0	39.2	-0.04	0.12	0.02	-0.02	0.15	0.02
3km	4.1	0.0	34.3	-0.06	0.05	0.03	-0.03	**0.03**	0.04
5km	3.2	0.0	26.7	-0.08	**0.04**	0.04	-0.04	**<0.01**	0.06
10km	2.7	0.0	16.4	-0.13	**0.01**	0.08	-0.07	**<0.01**	0.15
15km	2.2	0.0	14.3	-0.19	**<0.01**	0.15	-0.08	**<0.01**	0.15
20km	2.0	0.0	13.6	-0.27	**<0.01**	0.26	-0.10	**<0.01**	0.17
30km	1.7	0.0	10.6	-0.35	**<0.01**	**0.33**	-0.13	**<0.01**	**0.22**
40km	1.4	0.0	8.8	-0.42	**<0.01**	0.35	-0.13	**<0.01**	0.20
50km	1.2	0.0	7.1	-0.45	**<0.01**	0.34	-0.14	**<0.01**	0.18
60km	1.1	0.0	5.9	-0.47	**<0.01**	0.33	-0.13	**<0.01**	0.14
70km	0.9	0.0	4.9	-0.51	**<0.01**	0.32	-0.14	**<0.01**	0.14
80km	0.9	0.0	4.8	-0.54	**<0.01**	0.33	-0.15	**<0.01**	0.14
90km	0.8	0.0	4.9	-0.59	**<0.01**	0.36	-0.16	**<0.01**	0.15

Supplementary Table 2 Results of the Principal Components Analysis led on the 15 functional traits measured on 158 fish species and on the 11 functional traits measured on 46 mammal species. (a) Relative eigenvalues and percentages of variance explained by each axis without correction for negative eigenvalues. (b) Contribution of each continuous trait to the axes of the functional spaces. The table shows the direction cosines of the continuous variables on the PCoA axes, the determination coefficient (R^2) of the correlation between each trait and the ordination, as well as p-values indicating the significance of R^2 by comparing the observed R^2 with R^2 values obtained from 999 random permutations of the data. (c) Mean position of categorical traits on the two first axes of the PCoA, p-values are based on permutation tests of the data indicating if observed R^2 are higher than R^2 with randomly permuted data. *p < 0.05, **p < 0.01, ***p < 0.001.

a			
		Axis 1	***Axis 2***
Fish	Relative eigenvalues	0.37	0.24
	Percentage of variance	37.01	23.60
	Cumulative percentage of variance	37.01	60.61
Mammals	Relative eigenvalues	0.38	0.26
	Percentage of variance	38.47	25.78
	Cumulative percentage of variance	38.47	64.25

b					
	Functional traits	***Axis 1***	***Axis 2***	***R²***	***p-value***
Fish	Relative Eye size	-1.0	-0.07	0.28	0.001***
	Oral gape position	-0.58	0.82	0.49	0.001***
	Relative maxillary length	-0.42	0.91	0.30	0.001***
	Eye vertical position	0.94	-0.35	0.75	0.001***
	Body elongation	0.84	-0.54	0.21	0.001***
	Body lateral shape	0.93	-0.36	0.66	0.001***
	Pectoral fin vertical position	0.26	0.97	0.54	0.001***
	Pectoral fin size	1.0	-0.04	0.07	0.024*
	Caudal peduncle throttling	-0.51	-0.86	0.30	0.001***
	Maximum body length	0.22	0.97	0.03	0.051
Mammals	Litter or clutch size (n)	-0.58	-0.81	0.14	0.101
	Adult body mass (g)	0.84	-0.54	0.33	0.001***
	Gestation (d)	0.93	0.37	0.22	0.022*
	Longevity (y)	-0.56	-0.83	0.01	0.822

	c					
	Functional traits	***Categories***	***Axis 1***	***Axis 2***	***R^2***	***p-value***
Fish	Preferred substrate	hard	0.08	-0.05	0.19	0.001***
		none	-0.08	0.05		
		soft	-0.04	0		
	Motility	mobile	-0.10	-0.02	0.48	0.001***
		sedentary	0.12	0.01		
		migratory	-0.10	-0.02		
	Gregariousness	gregarious	-0.09	-0.07	0.36	0.001***
		solitary	0.07	0.09		
	Water column position	benthic	0.12	-0.12	0.68	0.001***
		benthopelagic	-0.14	0		
		demersal	0.11	0.12		
		pelagic	-0.15	-0.04		
	Territoriality	yes	0.14	0.17	0.37	0.001***
		no	-0.06	-0.04		
	Trophic level	omnivore	0.07	0.11	0.38	0.001***
		detritivore	0.17	-0.05		
		herbivore	0.10	-0.16		
		predator	-0.05	0.02		
Mammals	Diet	P	0.21	0.07	0.58	0.001***
		V	0.25	-0.07		
		I	0.13	-0.23		
		I+V	0.20	-0.23		
		P+I	0.06	0.06		
		P+I+V	-0.17	-0.06		
	Trophic level	herbivore	0.19	0.12	0.50	0.001***
		omnivore	-0.10	-0.08		
		carnivore	0.20	-0.17		
	Type of habitat	terrestrial	0.01	-0.02	0.22	0.001***
		terrestrial & freshwater	0.34	-0.20		
	Activity cycle	nocturnal	-0.12	0	0.35	0.001***
		diurnal	-0.01	-0.03		
		nocturnal & diurnal	0.24	-0.08		
	Terrestriality	ground dwelling	0.24	-0.06	0.51	0.001***
		above ground dwelling	-0.13	-0.02		
	Habitat breadth	1 habitat	0.14	-0.07	0.50	0.001***
		2 habitats	-0.11	-0.13		
		3 habitats	0.35	-0.18		
	Diet Breadth	1 diet	0.20	-0.18	0.37	0.007**
		2 diets	0.15	0.06		
		3 diets	-0.07	0.01		
		4 diets	-0.14	-0.12		
		5 diets	0.17	0.01		
		6 diets	-0.08	-0.05		

Supplementary Table 3 Mean percentage of upstream and upstream-downstream deforested surfaces due to gold mining and due to other human activities (agriculture, logging and/or urban areas). The Pearson correlations and associated p-values between the percentage of gold mined areas and deforested areas upstream and upstream-downstream of the sampling sites for each spatial extent are also indicated. These values were obtained with two-sided Pearson correlation tests. (a) Fish sampling sites (n = 64) and (b) mammal sampling sites (n = 74).

a

	Upstream				*Upstream - downstream*			
Spatial extent	***Gold mining deforestation (%)***	***Others human activity deforestation (%)***	***Correlation coefficient***	***p-value***	***Gold mining deforestation (%)***	***Others human activity deforestation (%)***	***Correlation coefficient***	***p-value***
0.5km	0.0	4.56	NA	NA	0	3.86	NA	NA
1.5km	0.01	4.69	0.07	0.60	0.13	3.92	0.23	0.07
3km	0.04	3.66	0	1	0.23	2.94	0.47	**<0.001**
5km	0.15	2.46	0.38	**<0.01**	0.28	2.23	0.54	**<0.001**
10km	0.50	1.49	0.80	**<0.01**	0.43	1.48	0.86	**<0.001**
15km	0.44	1.11	0.86	**<0.01**	0.48	1.19	0.87	**<0.001**
20km	0.40	0.84	0.85	**<0.01**	0.47	1.00	0.84	**<0.001**
30km	0.37	0.64	0.86	**<0.01**	0.44	0.86	0.84	**<0.001**
40km	0.34	0.55	0.85	**<0.01**	0.41	0.75	0.83	**<0.001**
50km	0.32	0.48	0.85	**<0.01**	0.39	0.66	0.82	**<0.001**
60km	0.32	0.44	0.83	**<0.01**	0.40	0.61	0.84	**<0.001**
70km	0.29	0.40	0.84	**<0.01**	0.40	0.60	0.86	**<0.001**
80km	0.28	0.37	0.84	**<0.01**	0.39	0.55	0.89	**<0.001**
90km	0.25	0.34	0.82	**<0.01**	0.38	0.52	0.90	**<0.001**

b

	Upstream				*Upstream - downstream*			
Spatial extent	***Gold mining deforestation (%)***	***Others human activity deforestation (%)***	***Correlation coefficient***	***p-value***	***Gold mining deforestation (%)***	***Others human activity deforestation (%)***	***Correlation coefficient***	***p-value***
0.5km	0	4.34	NA	NA	0	3.62	NA	NA
1.5km	0.01	4.76	0.06	0.60	0.11	3.96	0.22	0.06
3km	0.03	4.11	-0.01	0.92	0.20	3.08	0.41	**<0.001**
5km	0.13	3.06	0.27	0.02	0.25	2.46	0.49	**<0.001**
10km	0.47	2.27	0.36	**<0.01**	0.40	1.82	0.69	**<0.001**
15km	0.44	1.75	0.40	**<0.01**	0.44	1.48	0.64	**<0.001**
20km	0.46	1.51	0.37	**<0.01**	0.45	1.29	0.61	**<0.001**
30km	0.45	1.21	0.36	**<0.01**	0.43	1.11	0.58	**<0.001**
40km	0.41	0.98	0.42	**<0.01**	0.39	0.94	0.61	**<0.001**
50km	0.38	0.81	0.50	**<0.01**	0.37	0.82	0.61	**<0.001**
60km	0.36	0.71	0.55	**<0.01**	0.38	0.76	0.66	**<0.001**
70km	0.33	0.62	0.57	**<0.01**	0.37	0.70	0.71	**<0.001**
80km	0.32	0.57	0.68	**<0.01**	0.37	0.65	0.76	**<0.001**
90km	0.32	0.52	0.78	**<0.01**	0.37	0.61	0.81	**<0.001**

Supplementary Table 4 Morphological and ecological traits and their corresponding functions used to quantify the functional richness of (a) fish and (b) mammal communities.

a			
Functional trait	***Measure (ratios or categories)***	***Function***	***Type***
Maximum body length	Fish base (cm)	Synthetic: metabolism, trophic impacts, locomotion nutrient cycling	
Body elongation	Bl/Bd	Locomotion	Morphological
Eye vertical position	Eh/Bd		
Body lateral shape	Hd/Bd		
Pectoral fin vertical position	PFi/Bd		
Pectoral fin size	PFl/Bl		
Caudal peduncle throttling	CFd/CPd		
Relative eye size	Ed/Hd	Food acquisition	
Oral gape position	Mo/Bd		
Relative maxillary length	Jl/Hd		
Relative barbell length	Bbl/Bl		
Territoriality	Yes, no	Behavior	Ecological
Motility	Mobile, sedentary, migratory		
Gregariousness	Gregarious, solitary		
Position in the water column	Benthic, benthopelagic, pelagic, demersal	Habitat preference	
Preferred substrate	Hard, soft, none		
Trophic level	Herbivore, predator, omnivore, detritivore	Trophic level	

b		
Functional trait	***Measure or category***	***Description***
Litter or clutch size	N	Number of offspring per litter or number of eggs per clutch.
Adult body mass	G	Body mass using data from males, females, and/or unspecified adults.
Gestation length	Day	Length of time from conception or fertilization to birth.
Longevity	Age	Age at death.
Terrestriality	Ground dwelling, above ground dwelling	Degree of terrestriality of each species (among ground dwelling or above ground dwelling).
Trophic level	Herbivore, omnivore, carnivore	Trophic level of each species (among herbivore, omnivore, or carnivore).
Habitat breadth	. 1 – 3 habitats	Number of habitat layers (among ground dwelling, aquatic, and ground dwelling) used by each species. 1 – 3 habitat layers.
Diet breadth	1 – 6 diets	Number of dietary categories (among vertebrate, invertebrate, fruit, flowers/nectar/pollen, leaves/branches/bark, seeds) eaten by each species. 1 – 6 diet categories.
Activity cycle	Nocturnal, diurnal or nocturnal & diurnal	Activity cycle of each species (among nocturnal, diurnal or nocturnal & diurnal).
Type of habitat	Terrestrial, terrestrial & freshwater	Whether the species spends a significant amount of time in freshwater, and/or on land.
Diet	P,V,I, I+V, P+I, P+I+V	Diet consisting in vertebrate prey (V), invertebrate prey (I), plants and/or fungi (P).

FIGURES

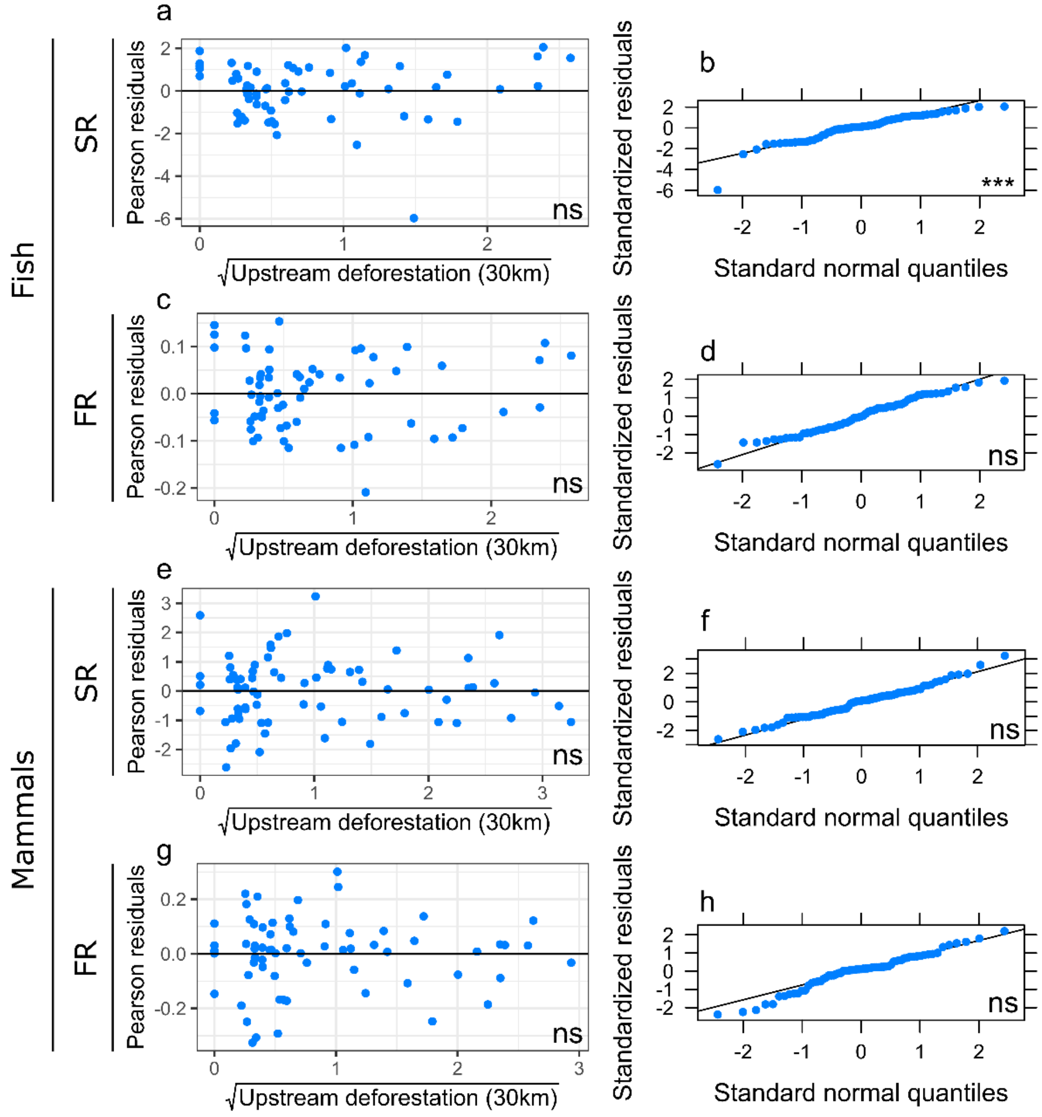

Supplementary Figure 1 Relationship between the residuals of the generalized mixed models and the upstream deforestation measured up to 30km upstream from biodiversity sampling sites (see Fig. 4). Homoscedasticity, equal variance of the residuals along the regression line (a, c, e, g). A generalized additive model was fitted onto the residuals to test for significant residual patterns. Results that are not significant indicate the absence of a residual pattern. ns = not significant. Quantile-quantile plot of the residuals (b, d, f, h). Normality of the residuals was assessed with Shapiro-Wilk tests. Results that are not significant indicate that residuals are normally distributed. ns = not significant, $p < 0.001$***. Fish: n=64 sites and mammals: n=74. The residuals of the models were normally distributed and showed no residual pattern with the exception of the fish species richness model (a, b). For this model, deviation from normality was due to a single outlier (corresponding to site M30, see Fig.1). Excluding this outlier from the analysis allows retrieving a normal distribution of the residuals.

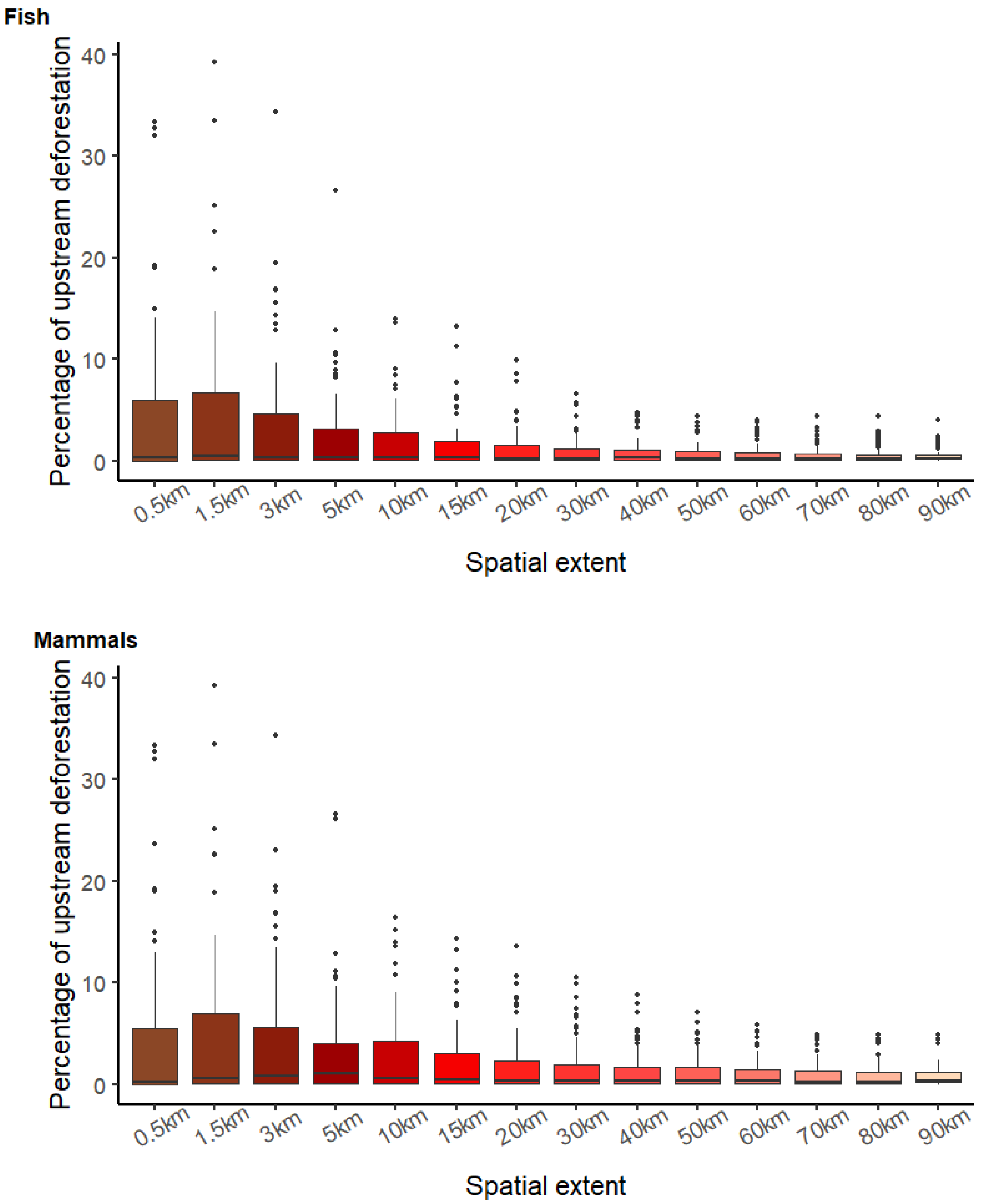

Supplementary Figure 2 Percentage of deforestation upstream from the sampling sites for each spatial extent for fish (top) and mammals (bottom) sampling sites. Color shades are consistent with the spatial extent as indicated in Fig. 3 (main text). Fish: n=64 sites and mammals: n=74. Boxplots indicate median (middle line), 25th, 75th percentiles (box), and maximum and minimum values (whiskers) as well as outliers (dots).

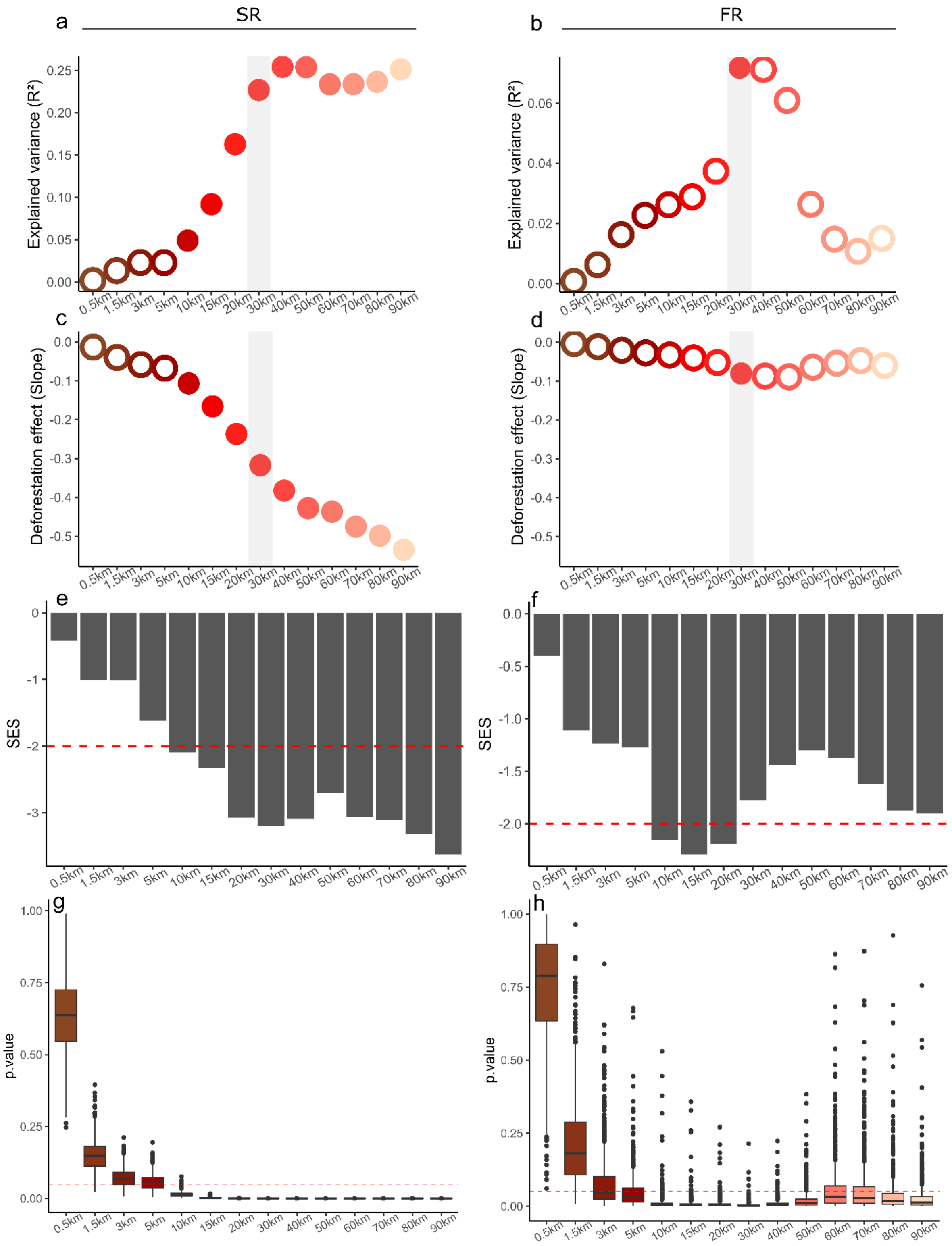

Supplementary Figure 3 Contribution of water-dependent mammals to the variance explained by the models. R^2 and the slopes of the (a, c) species richness (SR) and (b, d) functional richness (FR) models at each spatial extent excluding the five water-dependent species. For each spatial extent, a specific generalized linear mixed model accounting for site network position and basin identity as random effects was built (n=74 sites). Significant ($p < 0.05$) and non-significant ($p > 0.05$) models assessed with Wald's tests are indicated by filled and open circles, respectively. Middle panels show the standardized effect size (SES) of the explained variance between mixed models including and excluding

water-dependent species for species richness (e) and functional richness (d) (See Supplementary Note 1 for details). Red dashed lines refer to the 95% confidence intervals. Bottom panels display the distribution of p.values of the 999 generalized linear mixed models in which five random non-aquatic species were removed for species richness (g) and functional richness (h). Statistical significance was tested with Wald's tests. Red dashed lines correspond to the significance threshold of 0.05%. Boxplots of panels g and h indicate median (middle line), 25th, 75th percentiles (box), and maximum and minimum values (whiskers) as well as outliers (dots).

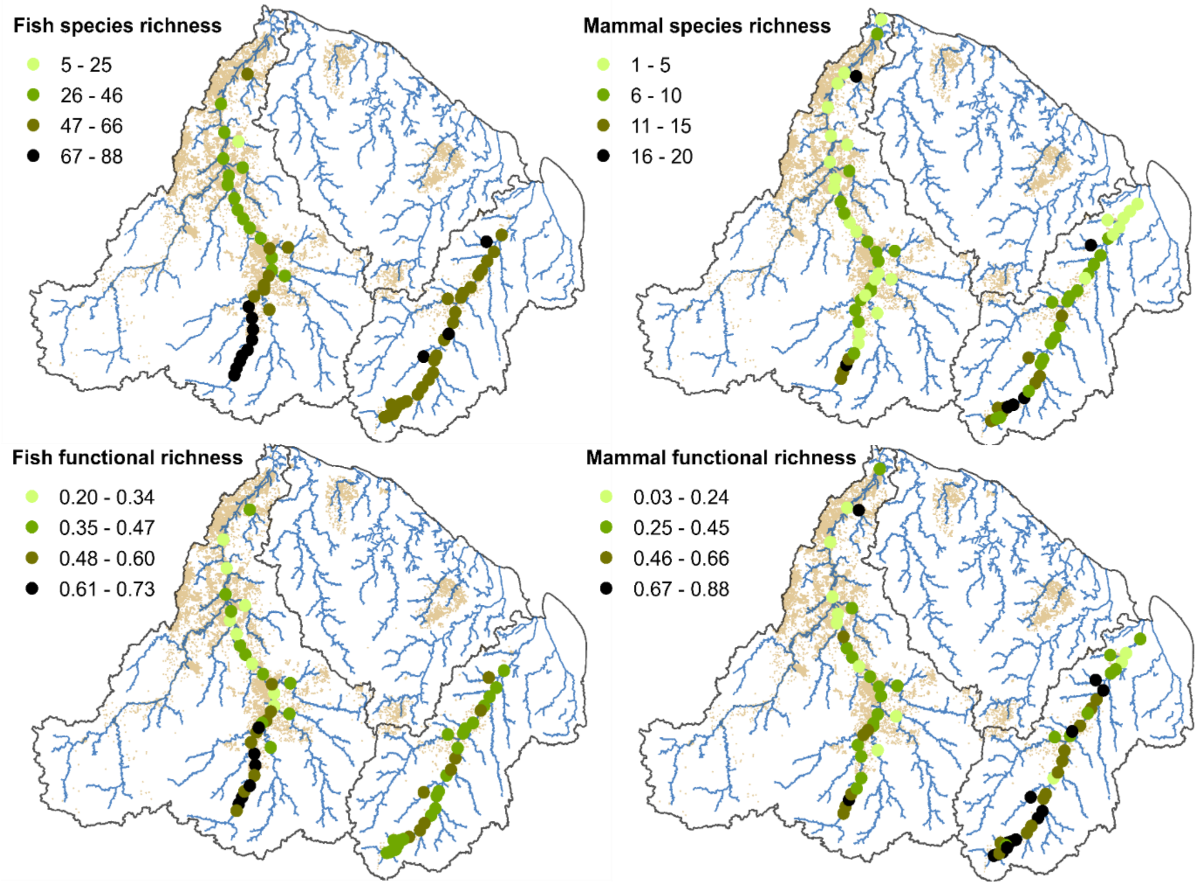

Supplementary Figure 4 Fish and mammal species and functional richness for each sampling site along the Maroni (left) and Oyapock (right) rivers. Fish: n=64 sites and mammals: n=74. Deforestation is represented by brown areas.

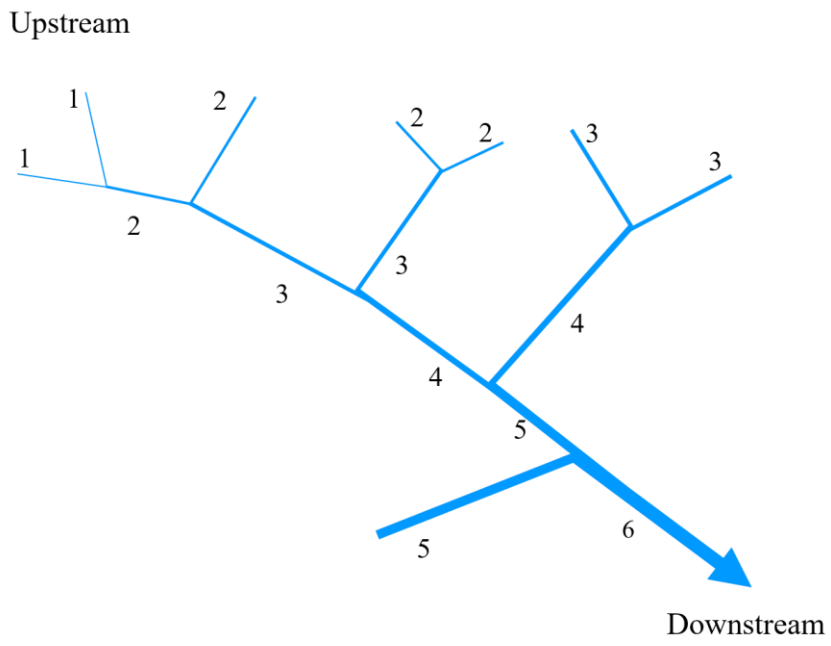

Supplementary Figure 5 Diagram illustrating Strahler river classification. The headwaters without any confluence are first-order streams. A river reaches a second-order at the confluence of two first-order streams. At a confluence of two streams with the same order, the downstream segment order increases by one. At a confluence, if the two streams are not of the same order then the highest numbered order is maintained on the downstream segment. Orders 1 and 2 account for streams and small rivers, orders 3 and 4 for medium-sized rivers and orders 5 and 6 for large rivers. Orders over 6 account for very large rivers.

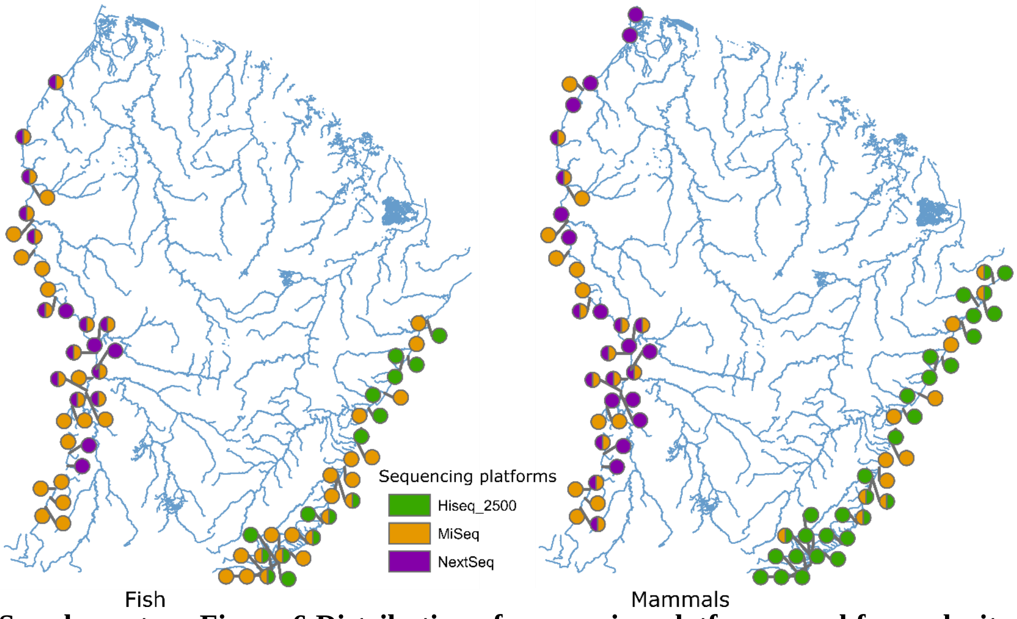

Supplementary Figure 6 Distribution of sequencing platforms used for each site for fish and mammals. For each site (Fish: n=64 sites and mammals: n=74), two independent site replicates were performed. Sites represented by one color have their two replicates sequenced with the same platform, while sites with two colors have their replicates sequenced with different platforms.

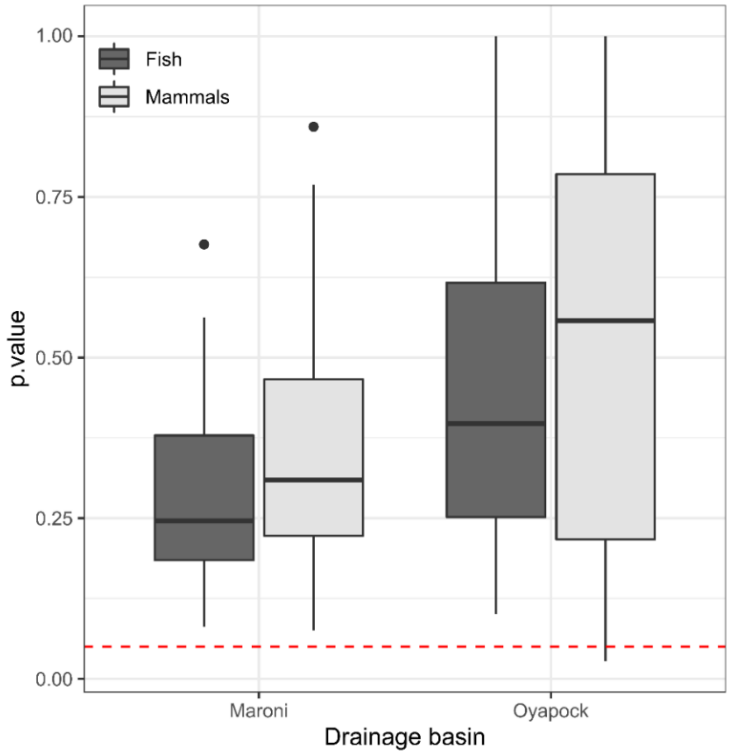

Supplementary Figure 7 Effect of the sequencing platform on species richness. The p-values (two-sided Mann-Whitney U tests) indicate the significance of the comparison between species number difference in site replicates assigned to the same sequencing platforms and species number difference in site replicates assigned to different sequencing platforms. Analyses were conducted separately for the Maroni and the Oyapock rivers, and for fish and mammals. The Red dashed line corresponds to the significance threshold of 0.05%. n=50 for each of the four categories (fish Maroni, mammals Maroni, fish Oyapock, mammals Oyapock). Boxplots indicate median (middle line), 25th, 75th percentiles (box), and maximum and minimum values (whiskers) as well as outliers (dots).

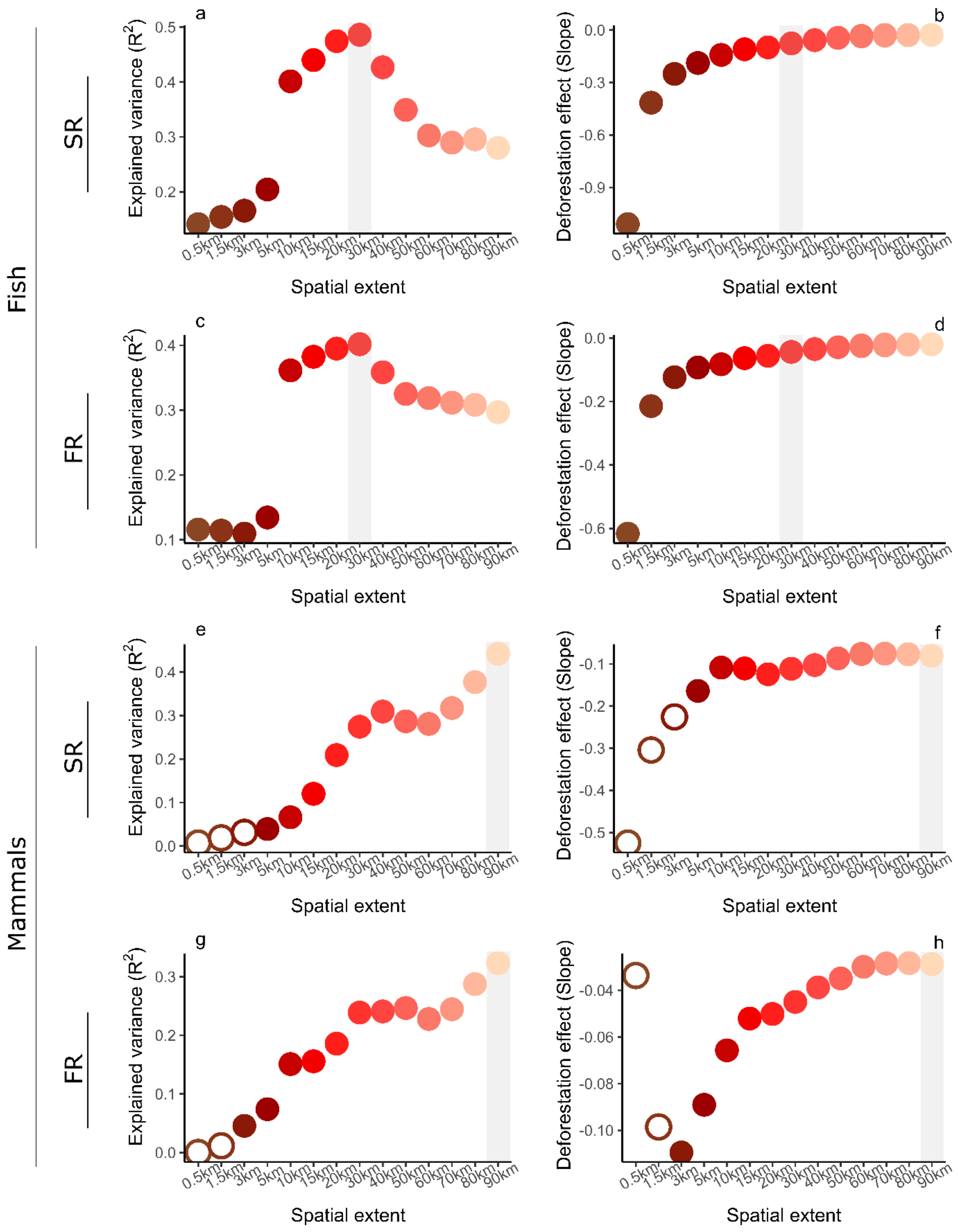

Supplementary Figure 8 Results of the mixed models relating diversity measures and absolutes values of deforestation areas upstream from the biodiversity sampling sites for each spatial extent. Left panels represent the explained variance by the mixed models (R^2), for each spatial extent. Right panels indicate the strength of the effect of deforestation on biodiversity (slope) for each spatial extent. (a, b) Fish species richness (SR) models; (c, d) fish functional richness (FR) models; (e, f) mammal species richness models; (g, h) mammal functional richness models. For each spatial extent, a specific generalized linear mixed model accounting for site network position and basin identity as random effects was built. Significant ($p < 0.05$) and non-significant ($p > 0.05$) models assessed with Wald's tests are indicated by filled and open circles, respectively. Fish: n=64 sites and mammals: n=74. Color shades are consistent with the spatial extent.

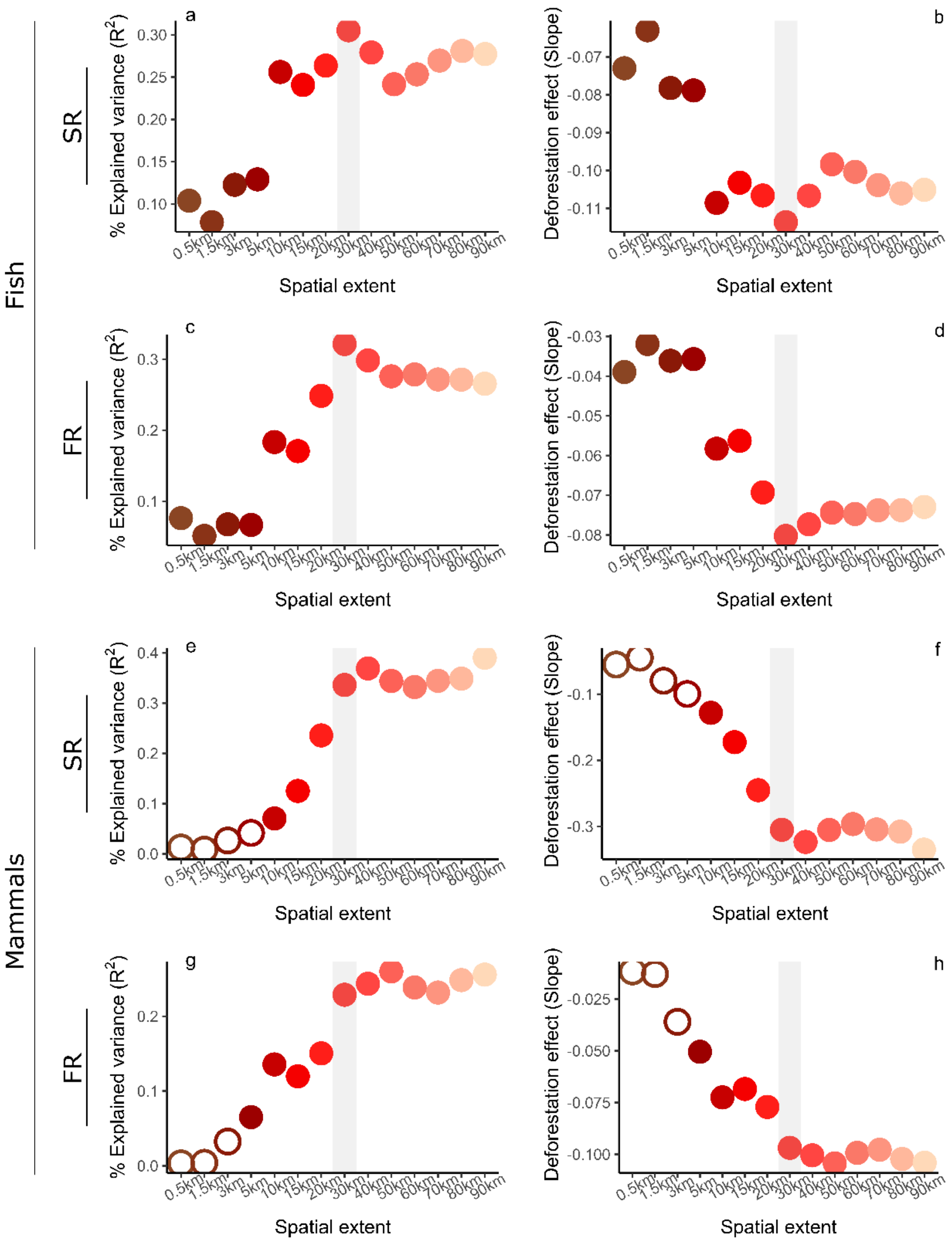

Supplementary Figure 9 Results of the mixed models relating diversity measures and scaled absolute deforested areas upstream from the biodiversity sampling sites for each spatial extent. Left panels indicate variance explained by mixed models (R^2) for each spatial extent. Right panels indicate the strength of the effect of deforestation on biodiversity (slopes). Spatial extents account for deforested areas upstream from eDNA sampling sites. (a, b) Fish species richness (SR) models. (c, d) Fish functional richness (FR) models. (e, f) Mammal species richness models. (g, h) Mammal functional richness models. For each spatial extent, a specific generalized linear mixed model accounting for site network position and basin identity as random effects was built for each biodiversity facet. Significant ($p < 0.05$) and non-significant ($p > 0.05$) models assessed with Wald's tests are indicated by filled and open circles, respectively. Fish: n=64 sites and mammals: n=74. Colour shades are consistent with spatial extents. Grey vertical bars indicate models with highest R^2 or R^2 reaching a plateau, with less than 5% variation between successive spatial extents.

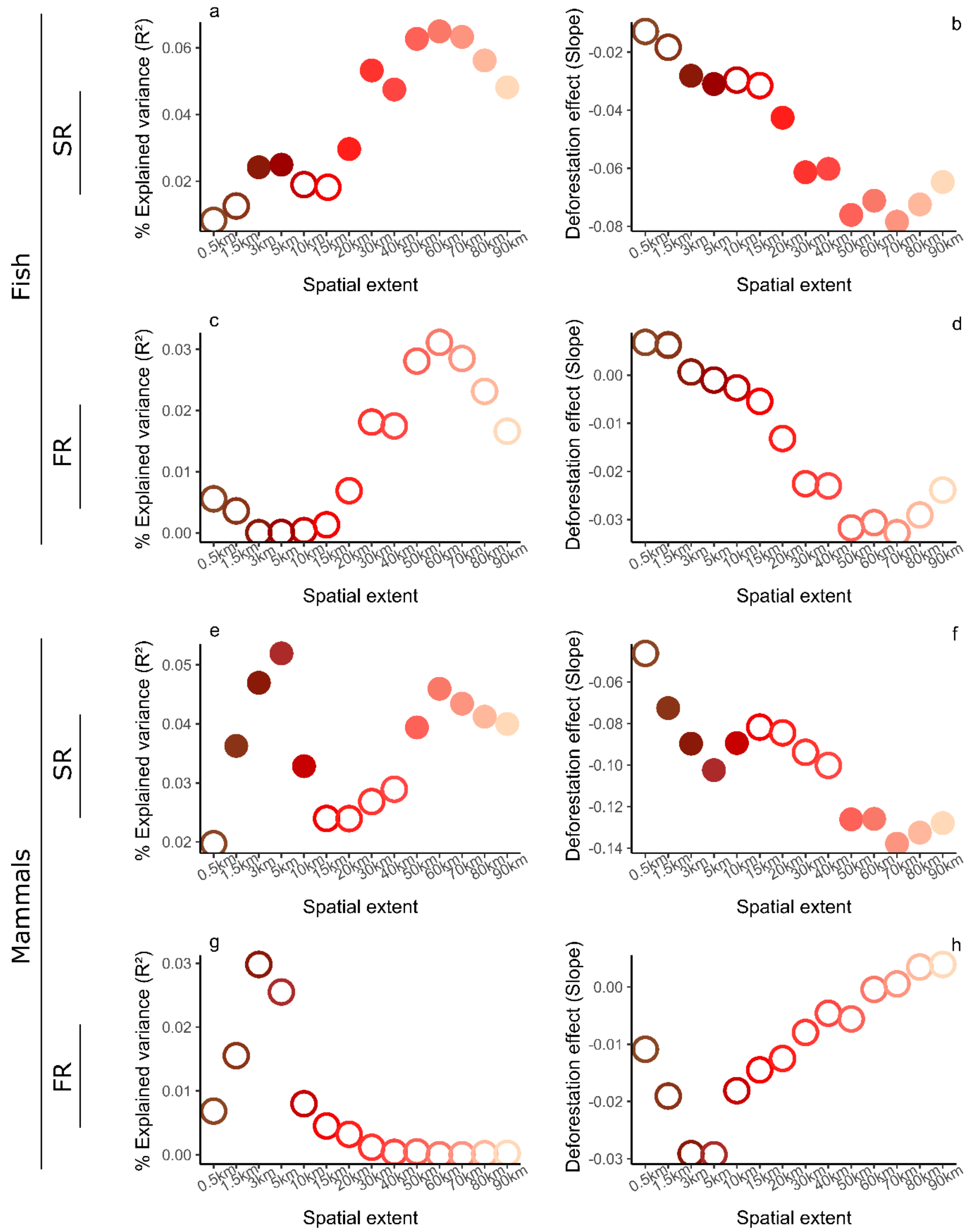

Supplementary Figure 10 Results of the mixed models relating diversity measures and the percentage of deforestation upstream and downstream sampling sites for each spatial extent. Spatial extents account for deforested areas upstream and downstream from the biodiversity sampling sites (i.e. not only accounting for hydrological connectivity). Left panels represent the explained variance (R^2) for each spatial extent. Right panels indicate the strength of the effect of deforestation on biodiversity (slope) for each spatial extent (see Fig. 3 for deforestation considering only areas upstream from the biodiversity sampling sites). (a, b) Fish species richness (SR) models; (c, d) fish functional richness (FR) models; (e, f) mammal species richness models; (g, h) mammal functional richness models. For each spatial extent, a specific generalized linear mixed model accounting for site network position and basin identity as random effects was built. Significant ($p < 0.05$) and non-significant ($p > 0.05$) models assessed with Wald's tests are indicated by filled and open circles, respectively. Fish: n=64 sites and mammals: n=74. Color shades are consistent with the spatial extent.

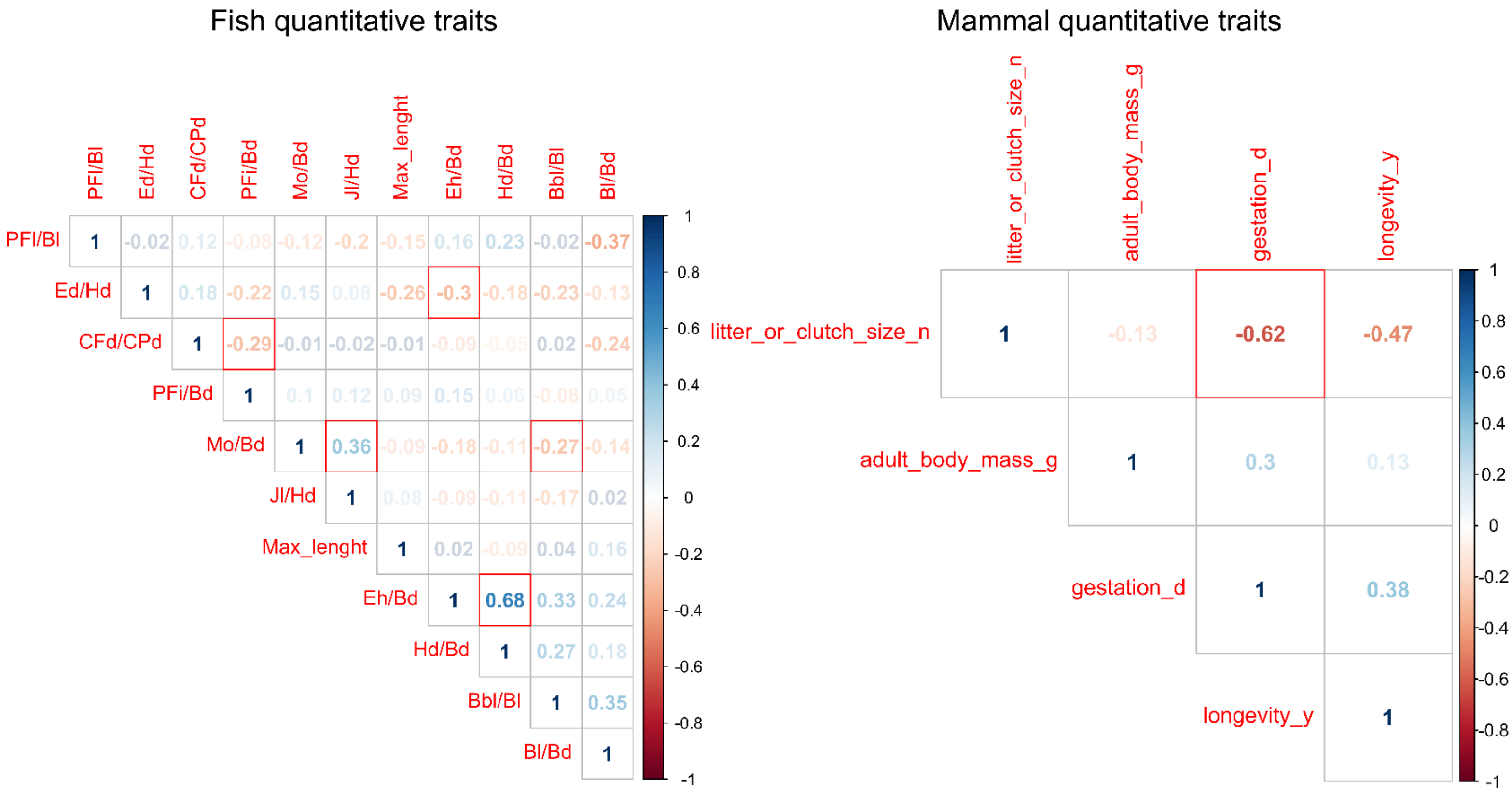

Supplementary Figure 11 Correlograms of the quantitative traits used to build the functional spaces and calculate functional richness for fish and mammals. Correlation coefficients (R) of pairwise combinations of traits were obtained with Kendall non-parametric statistical tests. R circled in red are statistically significant ($p<0.05$). Fish: n=158 species and mammals: n =46 species.
