# Supplementary material for: Low level of anthropization linked to harsh vertebrate biodiversity declines in Amazonia
Source: Nat Commun. 2022 Jun 7;13:3290. doi: 10.1038/s41467-022-30842-2 (PMC9174194; doi:10.1038/s41467-022-30842-2)
Supplement: Supplementary file 3 — Description of Additional Supplementary Files [file 41467_2022_30842_MOESM3_ESM.pdf]

File Name: Supplementary Data 1

Description: (a) Fish and (b) mammal coordinates on the PCoA and percentage of occurrence of each species in non-deforested and deforested sites. Occurrence percentages were calculated as the occurrence number of a species divided by the sum of occurrences of all species.

File Name: Supplementary Data 2

Description: Site metadata including latitude, longitude, hydrological and physicochemical characteristics, biodiversity results, upstream deforestation percentages, distance to the sea (in km) and upstream catchment area (in km^2). Species and functional richness are indicated for mammals and fish in each site.

File Name: Supplementary Data 3

Description: Sequencing information per sample for (a) fish, (b) mammals and (c) controls. The 12 PCR amplifications per sample were combined.

File Name: Supplementary Data 4

Description: Detected species by site matrices for (a) fish and (b) mammals. Read numbers after bio-informatic filtering are provided.

File Name: Supplementary Data 5

Description: (a) Fish and (b) mammal site subsampling analyses. Site combinations were designed using increasing minimum distances between sites (8 minimal distances ranging from 2 to 50 km). For each minimum distance, sites that were more distant than the considered minimum distance were randomly selected from the global set of sites to build a subset of site combinations. This step was repeated 50 times, giving rise to 50 subsets of sites for each minimum distance (except for minimal distances of 2 and 5 km where the number of possible combinations was lower, see Table S9). The species and functional richness of each site from each subset were then used in 14 linear mixed models (corresponding to the 14 spatial extents at which deforestation was considered). R^2 and slopes from each model were averaged for the 50 subsets. The site subsampling

was conducted separately for each taxon (fish and mammals) and each measure of biodiversity (taxonomic and functional richness). “Spatial extent” refers to the spatial extent to which deforestation percentages were considered, “Site number” refers to the number of sites randomly selected from the global set of sites, “Bootstrap number” refers to the number of sets of site combinations generated, “Slope” and “R^2” values are averaged values across the “n” subsets generated. Bold values of spatial extent reflect the spatial extent for which models provided the best R^2 (highest R^2 value, or stabilization of R^2 values reaching a plateau with less than 5% change in R^2 value between successive spatial extents).

File Name: Supplementary software 1

Description: R code for the main analyses of the study.
