# Supplementary material for: Low level of anthropization linked to harsh vertebrate biodiversity declines in Amazonia
Source: Nat Commun. 2022 Jun 7;13:3290. doi: 10.1038/s41467-022-30842-2 (PMC9174194; doi:10.1038/s41467-022-30842-2)
Supplement: Supplementary file 10 — Reporting Summary [file 41467_2022_30842_MOESM10_ESM.pdf]

# nature portfolio

Corresponding author(s): Opale Coutant

Last updated by author(s): Apr 18, 2022

# Reporting Summary

Nature Portfolio wishes to improve the reproducibility of the work that we publish. This form provides structure for consistency and transparency in reporting. For further information on Nature Portfolio policies, see our Editorial Policies and the Editorial Policy Checklist.

## Statistics

For all statistical analyses, confirm that the following items are present in the figure legend, table legend, main text, or Methods section.

| n/a | Confirmed | |
|---|---|---|
| ☐ | ☒ | The exact sample size ($n$) for each experimental group/condition, given as a discrete number and unit of measurement |
| ☐ | ☒ | A statement on whether measurements were taken from distinct samples or whether the same sample was measured repeatedly |
| ☐ | ☒ | The statistical test(s) used AND whether they are one- or two-sided<br>*Only common tests should be described solely by name; describe more complex techniques in the Methods section.* |
| ☐ | ☒ | A description of all covariates tested |
| ☒ | ☐ | A description of any assumptions or corrections, such as tests of normality and adjustment for multiple comparisons |
| ☐ | ☒ | A full description of the statistical parameters including central tendency (e.g. means) or other basic estimates (e.g. regression coefficient) AND variation (e.g. standard deviation) or associated estimates of uncertainty (e.g. confidence intervals) |
| ☐ | ☒ | For null hypothesis testing, the test statistic (e.g. $F$, $t$, $r$) with confidence intervals, effect sizes, degrees of freedom and $P$ value noted<br>*Give P values as exact values whenever suitable.* |
| ☒ | ☐ | For Bayesian analysis, information on the choice of priors and Markov chain Monte Carlo settings |
| ☒ | ☐ | For hierarchical and complex designs, identification of the appropriate level for tests and full reporting of outcomes |
| ☒ | ☐ | Estimates of effect sizes (e.g. Cohen's $d$, Pearson's $r$), indicating how they were calculated |

*Our web collection on statistics for biologists contains articles on many of the points above.*

## Software and code

Policy information about availability of computer code

| | |
|---|---|
| Data collection | No software was used for data collection. |
| Data analysis | -Codes for reproducing the results in this study are available in the supplementary and also from corresponding authors on reasonable request.<br><br>-All the necessary files to reproduce the analyses are available in the supplementary.<br><br>-Sequence reads were analysed using the ecoPCR software Version 0.2 and the programmes of the OBITools package version 1.2.11 following the protocol described in Valentini et al. 2016 [doi: 10.1111/mec.13428].<br><br>-Spatial analyses were performed using the GIS software ArcGIS 10.8. Sub-basins around sampling sites were delineated by applying a Flow Accumulation algorithm to the SRTM Global 30 m Model Elevation. Deforestation intensity in each spatial extent was quantified by summing deforested surfaces from three datasets obtained from Landsat satellite images and dividing the sum by the radius size of each spatial extent.<br><br>-Statistical and functional analyses were performed using R version 4.1.0 (2021-05-18):<br>-Generealized linear mixed models were built using the R package lme4 and the function lmer.<br>- $R^2$ were obtained using the r.squaredGLMM function from the MuMIn R package.<br>- The envifit function from the vegan R package was used to fit the variables (traits) onto the PCoA ordination and identify any correlations between the traits and the ordination axes. |

For manuscripts utilizing custom algorithms or software that are central to the research but not yet described in published literature, software must be made available to editors and reviewers. We strongly encourage code deposition in a community repository (e.g. GitHub). See the Nature Portfolio guidelines for submitting code & software for further information.

## Data

Policy information about availability of data

All manuscripts must include a data availability statement. This statement should provide the following information, where applicable:

- Accession codes, unique identifiers, or web links for publicly available datasets
- A description of any restrictions on data availability
- For clinical datasets or third party data, please ensure that the statement adheres to our policy

-All Illumina raw sequence data are available on https://doi.org/10.5061/dryad.pvmcvdnmr for fish samples
-All Illumina raw sequence data are available on https://doi.org/10.6084/m9.figshare.13739086.v6 for mammal samples.

- The reference database used for fish was an updated version of Cilleros et al. (2019) ( https://doi.org/10.1111/1755-0998.12900).
-The reference database used for mammals comprised the local database of French Guianese mammals ( https://doi.org/10.1111/2041-210X.12729), as well as all available vertebrate species in EMBL (https://www.embl.org).

- Fish maximum body length of each species was extracted from FishBase (www.fishbase.org)
- Mammal functional traits were obtained from three databases: Phylacine 1.2 ( https://doi.org/10.1002/ecy.2443), Amniote ( https://doi.org/10.1890/15-0846R.1) and PanTHERIA ( https://doi.org/10.1890/08-1494.1).

-Forest loss surfaces were obtained from the Global Forest Change dataset (DOI: 10.1126/science.1244693) .
-Surfaces deforested by gold mining activity in French Guiana, Suriname, and Northern Brazil were extracted from Rahm et al. (2014). Rahm, M. et al. Monitoring the impact of gold mining on the forest cover and freshwater in the Guiana Shield. Ref. 60, (2014).

# Field-specific reporting

Please select the one below that is the best fit for your research. If you are not sure, read the appropriate sections before making your selection.

☐ Life sciences ☐ Behavioural & social sciences ☒ Ecological, evolutionary & environmental sciences

For a reference copy of the document with all sections, see nature.com/documents/nr-reporting-summary-flat.pdf

# Ecological, evolutionary & environmental sciences study design

All studies must disclose on these points even when the disclosure is negative.

| | |
|---|---|
| Study description | This study proposes a framework to measure the strength and spatial extent of disturbance. We measured the relationships between local Amazonian fauna and the percentage of deforested surfaces from the immediate vicinity of biodiversity sampling sites to 90 km around. Taxonomic and Functional diversity were measured in 64 and 74 sites for freshwater fish and mammal communities, respectively, using eDNA data. Disturbance intensity was measured with Landsat satellite images at different spatial extents by widening the spatial extent in which disturbance intensity was assessed, from 0.5 km to a radius of 90 km from the biodiversity sampling sites. This provided measures of disturbance intensity (here the percentage of deforested surfaces) for each spatial extent. Mixed Models relating biodiversity (species and functional richness) and disturbance intensity were built for each spatial extent. River basin identity and site position in the upstream-downstream river network were included as random effects. The most relevant model indicates the representative spatial extent to measure disturbance and the slope of the relationship between disturbance and biodiversity in this model measures the disturbance strength. |
| Research sample | The dataset used for this study represents fish and mammal inventories using eDNA sampling on 64 and 74 sites, respectively, in two Amazonian rivers (The Maroni and Oyapock rivers). For both taxa, consequent molecular reference databases were available (see Cilleros et al. 2019 [doi: https://doi.org/10.1111/1755-0998.12900]; Cantera et al. 2019, Coutant et al. 2020 and Coutant et al. 2021). The Oyapock and Maroni rivers were chosen as a case study because even though they remain among the most pristine areas on earth, they are facing an unprecedented rise of human threats due to deforestation for agriculture, mining and urbanization. Additionally, these rivers are also recognised as hotspots of fish biodiversity in the Northern Amazonian region and benefit from extended knowledge on fish and mammal fauna. |

| | |
|---|---|
| Sampling strategy | Sample locations were selected to cover all accessible habitats in the Oyapock and Maroni rivers. Sampling was achieved while boating from downstream to upstream for each river, and the most upstream sites correspond to the limit of the navigable river course. Site locations were set to cover all the environmental and human contexts. To this aim, sites were located upstream and downstream from the major tributaries, the main natural barriers (rapids) and the main human settlements. We also sampled the downstream part of major tributaries, when access was possible by boat.<br>We filtered two replicates of 34 litres of water at each site. According to Cantera et al. (2019), this allows to inventory more than 75% of the expected fish fauna and provides a more comprehensive image of the fish species richness than capture methods. For mammals, Coutant et al (2021) showed that the same sampling protocol also provided an efficient inventory of both aquatic and terrestrial mammals. |
| Data collection | Data were collected by Sebastien Brosse, Jerome Murienne and Jean Baptiste Decotte. A peristaltic pump (Vampire sampler, Burlke, Germany) and a single-use tubing were used to pump the water into a single-use filtration capsule (VigiDNA 0.45 µm; SPYGEN, le Bourget-du-Lac, France). The input part of the tubing was placed few centimetres below the surface in zones with high water flow. Sampling was achieved in turbulent areas (rapid hydromorphologic unit) to ensure an optimal homogenization of the DNA throughout the water column. To avoid DNA contamination among sites, the operator always remained downstream from the filtration area and stayed on emerging rocks. At the end of the filtration, the filtration capsule was emptied of water, filled with 80 mL of CL1 conservation buffer (SPYGEN) and stored in the dark up to a month before the DNA extraction. |
| Timing and spatial scale | Sampling took place during the dry season (October- November) in 2017 and 2018 for the Maroni and Oyapock, respectively. In both rivers, sites were sequentially sampled from downstream to upstream, with 1 to 4 sites sampled per day, according to the travel time between sites. Sampling sites were located in two Amazonian rivers. The Maroni river measures 612 km from the source to the estuary and covers a surface of more than 68 000 km² in Suriname and French Guiana. The Oyapock River (404 km long and 26 800 km²) is located over Amapa (Brazil) and French Guiana. |
| Data exclusions | For freshwater fish analyses, 10 estuarine sites were discarded because our molecular reference database does not allow to detect marine and estuarine fish species. |
| Reproducibility | The results presented in this manuscript are obtained from the analysis of the outputs of mathematical models. They are thus fully reproducible (see R code and files in supplementary as well as all Illumina raw sequence data at https://doi.org/10.5061/dryad.pvmcvdnmr for fish samples and https://doi.org/10.6084/m9.figshare.13739086.v6 for mammal samples. The runs used for this study can be extracted using the sequencing information in Table S6. |
| Randomization | Three randomized analyses were conducted to demonstrate the robustness of our findings:<br><br>1) Effect of the sequencing platform on species richness (Supplementary Note 2 and Supplementary Figures 5, 6).<br>To distinguish the replicate effect (variability in the two replicates from the same site due to sampling and site effects) from the sequencing platform effect, we compared the difference in species number between replicates assigned to the same platform (accounting for the replicate effect only) to the difference in species number between the replicates assigned to different platforms (accounting for replicate and platform effects). Sites with replicates on the same platform were randomly selected to be compared to the same number of sites with replicates on different platforms. Then, for each site, we calculated the difference in species number between the two replicates and conducted a Mann-Whitney U test to compare the mean difference in species number between replicates assigned to the same platform to that of the replicates assigned to different platforms. We bootstrapped this procedure 50 times and displayed the distribution of p.values in Supplementary Figure 6. Analyses were conducted separately for the Maroni and Oyapock rivers and for the two taxa because different platforms were used.<br><br>2) Contribution of water-dependent mammals to the variance explained by the models (Supplementary Note 1 and Supplementary Figure 2).<br>We analysed the influence of water-dependent mammals on the explained variance retrieved from the mixed models to understand if water-dependent species drive the observed response to deforestation. We compared model results excluding the five water-dependent mammals (Giant otter (Pteronura brasiliensis), Neotropical otter (Lontra longicaudis), Water opossum (Chironectes minimus), Capybara (Hydrochoerus hydrochaeris) and Lowland tapir (Tapirus terrestris) to 999 sets of species combinations in which five non-aquatic mammals were randomly removed. Species and functional richness were calculated for each set and implemented in the 14 mixed models considering deforestation at different spatial extents. Then we calculated the Standardized Effect Size (SES) as $R^2$withtout water-dependent species – mean ($R^2$ random) /sd ($R^2$ random), (sd = standard deviation). SES < -2 indicates that water-dependent species contribute significantly more to the explained variance than nonaquatic species. SES > -2 indicates that the contribution of water-dependant species to the explained variance is not significantly different to that of nonaquatic species<br><br>3) Fish and mammal site subsampling analyses to test the effect of spatial autocorrelation on our results (Supplementary Data 5).<br>Site combinations were designed using increasing minimum distances between sites (8 minimal distances ranging from 2 to 50 km). For each minimum distance, sites that were more distant than the considered minimum distance were randomly selected from the global set of sites to build a subset of site combinations. This step was repeated 50 times, giving rise to 50 subsets of sites for each minimum distance (except for minimal distances of 2 and 5 km where the number of possible combinations was lower, see Supplementary Data 5). The species and functional richness of each site from each subset were then used in 14 linear mixed models (corresponding to the 14 spatial extents at which deforestation was considered). $R^2$ and slopes from each model were averaged for the 50 subsets. The site subsampling was conducted separately for each taxon (fish and mammals) and each measure of biodiversity (taxonomic and functional richness). |
| Blinding | Site location was replaced by a numerical code for molecular analyses. Therefore, the species inventories derived from environmental DNA data were obtained without prior knowledge of site location and human or environmental context. |

Did the study involve field work? [x] Yes [ ] No

## Field work, collection and transport

| | |
|---|---|
| Field conditions | eDNA sampling was achieved during the dry season (October- November) in 2017 and 2018 for the Maroni and Oyapock, respectively. During the days of sampling, temperatures ranged between 21 and 32 degree Celsius (°C) and no rain occurred. |
| Location | This study was conducted in two rivers located in the Northern East of the Amazonian region (sensu lato, including Guiana Shield and Amazon river drainage). The Maroni river measures 612 km from the source to the estuary and covers a surface of more than 68 000 km² in Suriname and French Guiana. The Oyapock river (404 km long and 26 800 km²) is located over Amapa (Brazil) and French Guiana. In all sites, the river was wider than 20 meters and deeper than one meter (Strahler orders 4 to 8).<br>Site location are presented on Fig. 2. Precise location (latitude, longitude, altitude) and environmental conditions are available in Table S5. |
| Access & import/export | No permits were required for the eDNA sampling and the access to all sites was legally permitted. The study complies with access and benefit permits ABSCH-IRCC-FR-246820-1 and ABSCH-IRCC-FR-245902-1, authorizing collection, transport and analysis of all environmental DNA samples used in this study. |
| Disturbance | No disturbance was caused by the field work. |

# Reporting for specific materials, systems and methods

We require information from authors about some types of materials, experimental systems and methods used in many studies. Here, indicate whether each material, system or method listed is relevant to your study. If you are not sure if a list item applies to your research, read the appropriate section before selecting a response.

### Materials & experimental systems

| n/a | Involved in the study |
|---|---|
| [x] | [ ] Antibodies |
| [x] | [ ] Eukaryotic cell lines |
| [x] | [ ] Palaeontology and archaeology |
| [x] | [ ] Animals and other organisms |
| [x] | [ ] Human research participants |
| [x] | [ ] Clinical data |
| [x] | [ ] Dual use research of concern |

### Methods

| n/a | Involved in the study |
|---|---|
| [x] | [ ] ChIP-seq |
| [x] | [ ] Flow cytometry |
| [x] | [ ] MRI-based neuroimaging |
