# Supplementary material for: Low level of anthropization linked to harsh vertebrate biodiversity declines in Amazonia
Source: Nat Commun. 2022 Jun 7;13:3290. doi: 10.1038/s41467-022-30842-2 (PMC9174194; doi:10.1038/s41467-022-30842-2)
Supplement: Supplementary file 2 — Peer Review File [file 41467_2022_30842_MOESM2_ESM.pdf]

nature portfolio

Peer Review File

Low level of anthropization linked to harsh vertebrate biodiversity declines in Amazonia

Open Access This file is licensed under a Creative Commons Attribution 4.0 International License, which permits use, sharing, adaptation, distribution and reproduction in any medium or format, as long as you give appropriate credit to the original author(s) and the source, provide a link to the Creative Commons license, and indicate if changes were made. In the cases where the authors are anonymous, such as is the case for the reports of anonymous peer reviewers, author attribution should be to 'Anonymous Referee' followed by a clear attribution to the source work. The images or other third party material in this file are included in the article's Creative Commons license, unless indicated otherwise in a credit line to the material. If material is not included in the article's Creative Commons license and your intended use is not permitted by statutory regulation or exceeds the permitted use, you will need to obtain permission directly from the copyright holder. To view a copy of this license, visit http://creativecommons.org/licenses/by/4.0/.

Reviewers' Comments:

Reviewer #1:
Remarks to the Author:
Major comments

I have reviewed ms NCOMMS-21-20430 entitled "Low level of deforestation causes harsh biodiversity decline in Amazonia". This is a very interesting piece of research whereby eDNA for fish and mammal species is analyzed following a spatial gradient of deforestation in two rivers of Amazonia. The results show that, as human impacts accumulate downstream, species occurrence decreases. I am unaware of similar results in the literature and these are of special importance given the disproportionate amount of research done on terrestrial aspects of tropical river-forest systems and the generalized lack of recognition in the "ecological impacts" literature of hydrologically-mediated effects such as the ones reported herein.

The data used in this ms is incredible, including 74 sampling sites along a rather spatially large area. The results make sense, and data analyses appear to be sound. The main aspects lacking in this ms are: consideration of the literature (lots of old, vaguely relevant papers cited, with more recent highly-relevant papers missing), identification of knowledge gaps the ms seeks to fill, identification of specific research questions, and description of rationale behind research objectives (see below). Because several of these issues were not done in the same degree of sophistication as the more technical sample and data analyses, the value of the research to the broad audience on tropical forest ecology and conservation is being missed. This is clearly observable in the discussion, which although essentially robust, fails to advance the field in the same degree the results reported would allow.

In particular, I feel there is a strong need to develop a general and conceptual framework of analysis. All the current text says is that impacts vary by area. Yet, the mechanics how those impacts occur are not described — are they hydrologically mediated? If so, then impacts are not a function of area per se, but rather downstream distance. How exactly, before knowing the results, can such impacts be expected to affect mammal species such as panthers, which have large home ranges and are "very" terrestrial organisms? There is a rationale but it is not being presented. All in all, what is the ecology behind the statistical results reported? There are elements in the literature to easily substantiate a rationale that would strengthen the discussion and the value of the research to others. I'd encourage the authors to check out Leal at al 2020. "Integrated terrestrial-freshwater planning doubles conservation of tropical aquatic species." Science. This is a highly relevant paper that was not considered, not just in terms of citation but mostly in terms of the general idea behind it. The general literature on hydrological connectivity also seems pertinent; the work of Pringle 2001 ("what is hydrological connectivity?" Ecological Applications) and many others is represented in the formulation of this research — yet it should underpin it in my view.

Considering this assessment, I feel this ms needs a major revision in order to attain its full impact. If that is done, I would expect it should rise to the level of this journal.

Details comments:

Abstract needs improvements on English

Introduction

Line 41: It states: "However, local biodiversity can also suffer from distant impacts through a cumulative effect of multiple distant disturbances." This should be re-written biodiversity suffers impacts from drivers located at distant locations.

The rationale of the introduction appears to be sound. It argues local impacts depend not only on local threats but also on impacts at distant locations that may propagate through the system. The article focuses on river systems and it is implied such impacts are propagated via downstream transport of sediment, nutrients, chemicals (which ones?), and energy. Yet, this is the only mechanistic discussion of how distant impacts may "travel" through ecosystems to have far-reaching effects on biodiversity. A number of examples where distant drivers of degradation are mentioned, but only superficially. The introduction then goes on to argue it is important to consider local and distant impacts, and the research is described without identification of a knowledge gap. Without establishing if the research done herein fills an existing lacunae in knowledge, the justification of this research is in the air.

This paper proposes on a novel approach to understand biodiversity impacts. The general approach described is standard. To me, the data used to employ it consisting of eDNA for fish and mammal species in 74 sites seems to be unique but not attempt is done to identify a lacunae on this.

As a scientist, and based on the information herein provided, I have lots of questions before I read the results: how much deforestation was available in the study sites? Depending on its amount, it could not be expected to have an impact. Are there other studies assessing deforestation effects? What exactly do they show? Has absolutely no prior study assess distant effects of deforestation on biodiversity? (This would seem hard to believe). Are the impacts assessed here expected to be due to habitat removal, decreases in water quality, or changes in hydrology? Without knowing these pieces of information, I am about to read results from an analysis that was vaguely defined. Precious space in the introductions spent explaining how buffer areas were delimited, and how relevant models were chosen, among other less crucial pieces of information.

Methods

Line 303: how can samples be retrieved? And how can a fish community be discarded? Clarify

Line 306: DNA samples cannot be 'achieved'; obtained, collected perhaps.

Line 208: after the confusing explanations above, here sampling is explained.

The text does not mention, but the spatial distribution of samples is impressive, as shown in Fig 2. I am still unclear as to what exactly constituted a sampling site and how fish and mammal species were sampled representatively in each site. No details are provided. eDNA of fish is collected in the water, but of mammals, how was it collected? It appears that it was also collected in the water, but there is no discussion of what portion of the mammal community this approach can be expected to reliably sample (all of them, or only aquatic mammals? I don't know…). The methods provide little information on the "ecology" of the work performed and are dominated by explanation of laboratory analyses, making it unclear how the data can be interpreted.

After four pages of mostly eDNA lab methods, the GIS analyses are explained, and again only the lab part of the work is described with no information describing the system of study, such as vegetation types, extent of deforestation, other drivers, spatial configuration of samples in relation to deforestation, etc.

Results

I see details of landscape measures and species identified by eDNA are given here, but considering these are not the aims of the ms, I suggest these data should be reported in methods, after all they do not answer the research questions.

Lines 89-103: interesting results reported here. My only concern is the language used; it focuses on the stats and not on answering the research questions. Referring to R2 as representative of model quality is new to me and an inadequate way of interpreting model performance.

Lines 104-110 report similar results for functional diversity. Ok. But it is unclear to me why use a taxonomic and a functional approach in the same analysis. Obviously the results were going to be semi-similar. The value of all results so far is being "washed down" by an excessive focus on statistics and not on the research questions. The audience drawn to this ms will seek information on deforestation effects on biodiversity, so focus on describing results should be as such.

Lines 111-121 report results that I cannot distinguish from prior results. Again, I am reading lost of statistical details but do not see which research question is being addressed here. As far as I can tell, this paragraph is repeating prior information.

And again focus on stats is hiding relevance of results reported in lines 122-142. What is the point of this section?

Lines 144-178 report on interesting patterns on functional diversity, but then again focus on stats is hiding the ecological relevance of the research.

Most of the above results are based on the underlying assumption that the observed deforestation is indeed the driving factor behind the fish and mammal patterns observed. Whether that is true has to be put to rest. It could be that fish and mammal species naturally decrease in occurrence downstream, or that other processes or drivers (that are not deforestation) have induced the observed patterns. There may be data that can be used to test if this is true or false, and if the results are indeed due to upstream deforestation.

Discussion

The discussion is ok, but I feel it lacks in providing novel insights on the topic largely because of the lack of specificity in framing the research questions and answering them. Given the research topic and lacunae were loosely defined, the discussion is somewhat unfocused. A resharpening of the research framing could substantially improve the contribution of this research.

Reviewer #2:
Remarks to the Author:
Review of “Low level of deforestation causes harsh biodiversity decline in Amazonia”
by Isabel Cantera et al. for Nature Communications

This manuscript addresses the spatial scale at which human disturbance measured from remotely sensed deforestation relates to fish and mammal species richness and functional diversity surveyed with eDNA. The manuscript will make an important contribution to the scientific literature because the large spatial extent of the eDNA sampling along two rivers in the Amazon is impressive and the results demonstrate the importance of distant disturbance on local fauna. My main concerns are that 1) ecological factors besides disturbance were neither measured nor considered as potential influences on local diversity 2) the introduction as currently written does not sufficiently motivate the study and 3) causality is at times attributed to deforestation despite acknowledgement of additional human disturbances. Careful attention needs to be paid to the use of the terms

“deforestation” and “disturbance” which seem to be used interchangeably throughout the manuscript, but which are indeed different (as the manuscript even acknowledges near the end in Lines 421-423: “deforestation intensity around each eDNA sampling site is here considered as a in integrative measure of environmental disturbances including gold-mining, logging, agriculture and human settlements”). A single term that represents what is measured and how it is interpreted should be defined upon first use and then used throughout the manuscript. Specific comments are below.

Lines 42-48: These examples are too specific for an opening paragraph that should convey the overarching themes of the paper and knowledge gap that the manuscript addresses

Line 54-55: This statement is tautological

Line 49: A focus on riverine ecosystems here does not set the reader up for a manuscript that quantifies mammal species richness and functional diversity. Furthermore, the final sentence of the paragraph and the final sentences of the final introductory paragraph seem to indicate that the manuscript is a methods paper, which is not its main contribution. I suggest moving the final sentence of the introduction to the discussion section and expanding upon the potential applications and extensions of this framework there.

Lines 99-103: Additional factors besides deforestation/disturbance might explain these results. For example, if the spatial extent under consideration is larger than the geographic range of some species, then that could influence these results. To what extent might that be a factor in this study? Are there environmental gradients that might influence these results via environmental filtering and species sorting? What about dispersal limitation?

Lines 111-121: It’s entirely unclear why upstream rather than downstream would matter for most mammals. Later on in the manuscript there is some mention of water dependent mammals, but nevertheless most mammals would likely be unaffected. The influence of water dependence could be testing by removing the water dependent species and examining the results in their absence. If removing them lowers the explanatory power of the model, then that would bolster the arugment.

Lines 133-143: Why turn a continuous variable into a binary category here? Furthermore, since deforestation is considered a composite variable from other types of disturbance too, this seems like an arbitrary cutoff (nor are references provided for the background rate of tree falls). “Pristine” is a misleading term and should be avoided.

Paragraphs starting with Line 144 and Line 160: The final sentences of each of these paragraphs is the key result that should be highlighted in the main text. The other descriptions can be moved to figure legends or supplemental information.

For the eDNA results, how far can eDNA travel? How certain are the “absences”? Is there any reason to think that certain taxa or functional groups would be more affected by

sampling biases?

Lines 188 & 190: Provide the median alongside the range values.

Lines 195-199: These sentences should be used in the introduction as part of the justification for a composite variable

Lines 208-210: This is an overstatement and should be removed or toned down

Lines 268-273: Do references #43 and #37 provide any insight into additional ecological mechanisms or environmental variables that may influence the results reported in this study? How/in what direction would these limitations influence the results?

Line 408-416: What was the correlation between the GFC and the gold-mining data? What is the justification for incorporating a separate deforestation layer for 2000?

Lines 494-497: Provide distributions in supplementary material. References for 0.4 as background rate?

Reviewer #3:

Remarks to the Author:

Within the last decade, the use of environmental DNA has become a key approach to study the composition and diversity of aquatic ecosystems. More recently, scientists also started to use eDNA to assess how environmental factors, such as contemporary or past land-use, are leaving imprints in ecological communities detectable by environmental DNA. For example, studies have found that certain agricultural practices (assessed by remote sensing) affect the structure and composition of microbial communities assessed through eDNA samples. Similarly, past land-use practices could be reconstructed from eDNA sampled from soil (e.g., Foucher et al. 2020 Scientific Reports). Thereby, it has not only been postulated that eDNA may transform how animal and plant communities are surveyed, but that it also can be used to study large-scale, and possibly cross-ecosystem dynamics.

In the current manuscript, eDNA samples taken along two rivers in South America are used to assess the possible impact of deforestation on fish and mammal communities. Fish and mammal communities reconstructed by eDNA were found to show a signal of changed (decreased) diversity when there were even relatively low levels of deforestation upstream.

This is a neat study, with potentially very interesting results, and the manuscript is generally well-written. I especially liked the use of eDNA to address a question of high relevance in conservation biology, namely how much land-use change (or how little) it takes to leave an imprint on organismal communities in whole catchments. Overall, I was satisfied reading the study. However, I also have a series of major points I consider important to be addressed, specifically referring to a) giving more data/results on the sequencing (to clarify if or not

some of the patterns observed could be driven by different sequencing platforms used), b) better integration and discussion of the spatial patterns (due to eDNA transport, but also due to null-expectations on distribution of biodiversity in dendritic networks), and c) better integration in the literature on biodiversity patterns expected in such dendritic riverine systems, also differentiating aquatic and terrestrial biodiversity (fish vs. mammals).

I have relatively extensive comments below, and realise that this will require some work/additional analyses/more data presented in the manuscript. If all my points can be resolved, I think this may become an important study (thus, my extensive comments are truly meant in a supportive manner! I would not comments so extensively if I wouldn't find the manuscript interesting).

Major comments:

1) The study looked at disturbance intensity (% deforestation) in spatial windows of increasing radius (from 0.5 to 90 km) away from the sampling sites (lines 58-62). Thus, if I understand it correctly, the % of deforested area is calculated for differently sized sections, meaning that the same percent of deforestation can represent very different areas (percent is not based on the same denominator). Comparing percentages with different denominators is generally problematic, but in this case it assumes a relative (and linear) effect of % area deforested of total area on local communities, and not an absolute area effect. I think this would be something that needs to be shown: is percentage or total area deforested a better predictor, and at what distance? This may be especially relevant for mammals, as the terrestrial area used is their habitat (and thus changes in land cover reduce their habitat, while for fish it is mostly linked to different run-offs and possible changes in the water chemistry).
Currently, it is only the relative effect of deforestation (lines 88-91), showing that % deforestation predicts fish species richness. While I think this is good, I think it should also be done with absolute values of deforestation.

2) Spatial autocorrelation of upstream catchment land-use. To my understanding (based on reading the manuscript) it seems that all sites were sampled along the mainstem of two major rivers. This means that sites (or their upstream land-use/upstream catchment) were hierarchically nested. While this is inherent to such a sampling design, it also means that upstream land-use change may affect multiple sites, similarly to a Russian doll. This results in two possible caveats:
Firstly, the same land-use (e.g. patches of deforestation) will be used as a co-variate for multiple sites, which would violate statistical independence (Land-use changes were assessed up to 90 km radius upstream of a sites; based on the information given it seems like sampling sites are often closer to each other than 90 km apart, which would mean that the same land-use change patch would affect multiple downstream sites).
Integrating this hierarchical sampling in an adequate manner is non-trivial (and it also depends on the distances between sites: if land-use effects were only studied as spatial scales much closer to a site than the distance between adjacent sites, this may be

neglectable), but should be addressed.
Secondly, transportation of eDNA along the stream means that the same upstream land-use change may affect multiple downstream patches (with decreasing relevance for further downstream sites, see e.g., Carraro et al. 2020 Nature Communications). This is something that should be addressed in the statistical model and also more extensively discussed. I would also strongly recommend to give a map in the supplement with all sites highlighted and numbered (to link to necessary supplementary data to be added on sequencing and biodiversity results at the site level, see below).

3) Information on the sites sampled is unclear and insufficient. While it says that in total 74 sites were sampled (lines 295–298), I was not able to find a map in which site id and position could be seen. Maybe I didn't do it correctly, but the CEBA geoportal site given showed me a map with many more sites, and they were also distributed outside these two rivers mentioned.
I would expect a map in the supplement (or even main manuscript) in which all sites are shown (and numbered, to compare to the sequencing data, see comment below), and to give more information on the sites' position, distance and hydrologic variables (mean, sd, min and max distance between adjacent sites, upstream catchment area of each site, etc). From the map I saw it seems that sites were not evenly spaced along the river but distances between adjacent sites varied by at least a factor of 10. Because eDNA is transported (e.g., Pont et al. 2018 Scientific Reports), this may be problematic, and should be discussed.

4) I was impressed by the amount of samples taken and the sequencing conducted. This is indeed a massive study. However, I was also very surprised to see that the sequencing was done on very different platforms. Libraries were sequenced on Illumina HiSeq2500, MiSeq, and NextSeq. I realise that such an extensive sampling involves sequencing multiple libraries. However, different sequencing platforms introduce non-random variation and potentially bias the results, and think here it is very critical to give extensive further information to show if and how data are comparable. Firstly, I would expect a table showing which samples were sequenced on which platforms (it seems that there was no randomization of samples across platforms, which is regrettable; for example, it would be problematic if more downstream sites were sequenced with one platform, and more upstream sites with another platform). Secondly, it would be necessary to show that the sequencing output (number of reads per sample is comparable across the different platforms (i.e., that there is no effect of sequencing platform on the sequencing output). Thirdly, it would be ideal to have at least some samples spread across platforms, to show that there were no biases in the sequencing (also because different reagent kits were used to do library preparation and the sequencing). The high heterogeneity in sequencing platforms used is a major concern to me, as it may create artifacts in the patterns. For example, if the sequencing of different samples was not randomized but follows the longitudinal sampling along the streams (i.e., downstream samples sampled on one platform, upstream samples on another), effects of position (and thus upstream land-use) may be confounded with the sequencing platform. I realize that it will not be possible to redo the sequencing (and I am for sure not asking for it, because it is a very impressive amount of sequencing done!), but I would expect a thorough analysis

showing that there is no artifact patterns created due sequencing platforms.
For example, an analysis/figures for the supplement showing the sequencing depth (read numbers), taxa assigned etc. for samples run on the different platforms (can be done in a simple anova, where read numbers, taxa richness etc. is compared against sequencing platform type, or even better, in a lme, where the run identity is even added as further possible confounding factor). This is needed to show that the patterns are not artifacts/driven by the platform type.

5) Do I read it correctly that in total fourteen negative controls and four negative PCR controls were used only? Given that there were in total 18 libraries sequenced (10 fish, 8 mammals), this mean that there was only one or two negative control in each of these libraries? While I personally do not think that negative controls can tell us everything about the quality and feasibility of a sequencing run, I still think this is at the very low (too low?) end. I realise that it will not be possible to change. However, I would firstly expect that the results of negative controls (number of reads, possible contaminations etc) are shown in more detail (table), and secondly that this low number of controls is discussed. It is a bit regrettable that in such a massive and really impressive effort of sequencing there was only little attention given to the negative controls.

6) I was missing all relevant and necessary information on the sequencing results. Specifically, I was missing tables (in the supplement) giving raw read numbers, read numbers after the most important bioinformatic steps, species richness numbers etc. per site (for example, in results lines 77-80, I would expect a table in the supplement that gives for each site at least the number of fish, or even better, the identity of fish. A simple site by species occupancy matrix for the supplement would do this job, and should be the standard for such studies). Now, this information is only given in a very concerted manner (mean across all sites). However, understanding the plausibility of such datasets crucially depends on a balanced sequencing depth, and a good understanding of the variation in sequencing output across different sites. To me, such tables synthesizing the raw data at the per site-level are essential.

7) The study compares position/extent of upstream land-use change to changes in communities assessed per site downstream. There is two possible explanations on how such a pattern emerges: Firstly, all the upstream changes together shape the downstream community assessed. Secondly, communities in the river are shaped along the stream based on adjacent land-use, and this signal is then (through transport of eDNA) integrated over space and collected in one sample. Based on knowledge of eDNA transport in streams, the second seems much more plausible to me (and I also think that this is how the authors see it). Importantly, however, such a spatial integration also directly implies that distance and position of all upstream sources (of eDNA) will together shape the locally assessed signal. Recent work (e.g. Carraro et al. 2020 Nature Communications) showed how hydrologic models can be used to adequately reconstruct eDNA input and transportation, and thus to better link the eDNA signal to upstream land-use type (or land-use change). While it may go beyond the scope of this manuscript to integrate such a modelling approach, I think the

hierarchic structure of their sampling design would at least require a discussion on hydrologic transport, and possible effects on the eDNA signal.

8) The finding that fish community richness was affected by deforestation is very interesting, and the explanation on a decrease due to loss of species (and not turn-over; lines 151-157) is plausible.
However, richness of fish (and other organisms) in dendritic networks is not random. Generally, richness is highest at downstream sites, both due to the habitat-size effect of large rivers, as well as a better connectivity. A decrease of richness with upstream positions is rather universal in fish. Importantly, anthropogenic effects in/along riverine networks are also not random, and often stronger at more downstream/lowland positions. Disentangling this would be interesting, and I see two opportunities with this dataset: firstly, it would be interesting to analyse richness with position in the riverine network (there is extensive reviews and opinion papers describing expectations on biodiversity patterns in riverine networks). Secondly, such an analysis could also include null models: if richness is (was) higher in pristine downstream sites, and if these are more affected by human activities, then the effect of deforestation could even be stronger (as they may spatially coincide). Discussing possible null-model expectations or, even better but maybe a bit too much for the manuscript, running a neutral meta-community model to create null-model expectations on richness across the network and then map observed diversity and changes thereof would add a neat angle to the manuscript, such that it would be well-embedded in the literature on biodiversity patterns in dendritic metacommunities.

Minor comments:
9) I recommend to give not only the names of primer used, but to actually give the primer sequence (this can also be done in the supplement). There is many, even closely similar, primers used to detect fish, and giving the nucleotide base sequence would be helpful and is a good practice.

10) Lines 113-114: What is the effect of disturbance (deforestation) on local diversity vs. a change due to transportation of eDNA?

11) Deforestation and other land-use changes are often associated with increasing run-offs of humic acids into the rivers. These humic acids are very well known inhibitors in PCR reactions (Uchii et al. 2019 Environmental DNA) and thus may directly affect sequencing output. Are there any data available on water quality/water chemistry, teasing apart effects of land-use change on biodiversity vs. simply detecting less biodiversity due to effects of land-use change on water chemistry interfering with PCR?

Literature mentioned:

Carraro et al. 2020. Environmental DNA allows upscaling spatial patterns of biodiversity in freshwater ecosystems. Nature Communications 11: 3585.

Foucher et al. 2020. Persistence of environmental DNA in cultivated soils: implication of this memory effect for reconstructing the dynamics of land use and cover changes. Scientific Reports 10: 10502.

Pont et al. 2018. Environmental DNA reveals quantitative patterns of fish biodiversity in large rivers despite its downstream transportation. Scientific Reports 8: 10361.

Uchii et al. 2019. Comparison of inhibition resistance among PCR reagents for detection and quantification of environmental DNA. Environmental DNA 1:359-367.

Point-by-point response to reviewer comments

Reviewer #1 (Remarks to the Author):
Major comments

I have reviewed ms NCOMMS-21-20430 entitled "Low level of deforestation causes harsh biodiversity decline in Amazonia". This is a very interesting piece of research whereby eDNA for fish and mammal species is analyzed following a spatial gradient of deforestation in two rivers of Amazonia. The results show that, as human impacts accumulate downstream, species occurrence decreases. I am unaware of similar results in the literature and these are of special importance given the disproportionate amount of research done on terrestrial aspects of tropical river-forest systems and the generalized lack of recognition in the "ecological impacts" literature of hydrologically-mediated effects such as the ones reported herein.

The data used in this ms is incredible, including 74 sampling sites along a rather spatially large area. The results make sense, and data analyses appear to be sound. The main aspects lacking in this ms are: consideration of the literature (lots of old, vaguely relevant papers cited, with more recent highly-relevant papers missing), identification of knowledge gaps the ms seeks to fill, identification of specific research questions, and description of rationale behind research objectives (see below). Because several of these issues were not done in the same degree of sophistication as the more technical sample and data analyses, the value of the research to the broad audience on tropical forest ecology and conservation is being missed. This is clearly observable in the discussion, which although essentially robust, fails to advance the field in the same degree the results reported would allow.

In particular, I feel there is a strong need to develop a general and conceptual framework of analysis. All the current text says is that impacts vary by area. Yet, the mechanics how those impacts occur are not described — are they hydrologically-mediated? If so, then impacts are not a function of area per se, but rather downstream distance. How exactly, before knowing the results, can such impacts be expected to affect mammal species such as panthers, which have large home ranges and are "very" terrestrial organisms? There is a rationale but it is not being presented. All in all, what is the ecology behind the statistical results reported? There are elements in the literature to easily substantiate a rationale that would strengthen the discussion and the value of the research to others. I'd encourage the authors to check out Leal at al 2020. "Integrated terrestrial-freshwater planning doubles conservation of tropical aquatic species." Science. This is a highly
relevant paper that was not considered, not just in terms of citation but mostly in terms of the general idea behind it. The general literature on hydrological connectivity also seems pertinent; the work of Pringle 2001 ("what is hydrological connectivity?" Ecological Applications) and many others is represented in the formulation of this research — yet it should underpin it in my view.

Considering this assessment, I feel this ms needs a major revision in order to attain its full impact. If that is done, I would expect it should rise to the level of this journal.

Thank you for your encouraging comments. We took a particular attention to better introduce our research questions and the knowledge gaps filled by our paper. The literature you suggested was particularly relevant and helped us to reorganize the manuscript. In particular, Leal et al.[1] was of great help to clarify our rationale and highlight our main findings. We also updated the references cited and now refer to the most recent and relevant papers. We rewrote and reorganised large parts of the introduction and of the discussion to clarify our research questions and strengthen the discussion. We hope you will find those changes adequate.

Please find below our point by point responses to your comments.

Details comments:

Abstract needs improvements on English

The abstract was modified, and English language was checked here and throughout the manuscript.

Introduction

Line 41: It states: “However, local biodiversity can also suffer from distant impacts through a cumulative effect of multiple distant disturbances.” This should be re-written biodiversity suffers impacts from drivers located at distant locations.

As the introduction changed substantially, this sentence was removed.

The rationale of the introduction appears to be sound. It argues local impacts depend not only on local threats but also on impacts at distant locations that may propagate through the system. The article focuses on river systems and it is implied such impacts are propagated via downstream transport of sediment, nutrients, chemicals (which ones?), and energy. Yet, this is the only mechanistic discussion of how distant impacts may “travel” through ecosystems to have far-reaching effects on biodiversity. A number of examples where distant drivers of degradation are mentioned, but only superficially. The introduction then goes on to argue it is important to consider local and distant impacts, and the research is described without identification of a knowledge gap. Without establishing if the research done herein fills an existing lacunae in knowledge, the justification of this research is in the air.

We deeply modified the introduction to provide a theoretical framework on the hydrologically-mediated impacts (lines 44-46) before reviewing the current knowledge on these impacts (lines 47-51) and then identify the knowledge gap we fill in this paper (lines 51-54).

This paper proposes on a novel approach to understand biodiversity impacts. The general approach described is standard. To me, the data used to employ it consisting of eDNA for fish and mammal species in 74 sites seems to be unique but not attempt is done to identify a lacunae on this.

We now underlined in the introduction that eDNA provides a unique opportunity to assess aquatic and terrestrial organisms in the same samples and thus to achieve cross ecosystem studies (lines 85-87).

As a scientist, and based on the information herein provided, I have lots of questions before I read the results: how much deforestation was available in the study sites? Depending on its amount, it could not be expected to have an impact. Are there other studies assessing deforestation effects? What exactly do they show? Has absolutely no prior study assess distant effects of deforestation on biodiversity? (This would seem hard to believe). Are the impacts assessed here expected to be due to habitat removal, decreases in water quality, or changes in hydrology? Without knowing these pieces of information, I am about to read results from an analysis that was vaguely defined. Precious space in the introductions spent explaining how buffer areas were delimited, and how relevant models were chosen, among other less crucial pieces of information.

Despite a detailed theoretical knowledge of the distant impacts of hydrologically-mediated disturbances, we were unable to find prior studies measuring the distant effects of deforestation on biodiversity. We agree it is hard to believe, but despite a detailed literature research, we did not find references on this point.
Our study gains a high interest for the last wilderness areas of the globe that are facing rises in local deforestation. This deforestation threatens local river habitats and the biodiversity they host, but also, as demonstrated in our paper, threatens both aquatic and terrestrial nearby faunas through distant downstream impacts. Such hydrologically-mediated impacts are multiple, including hydrological and chemical changes affecting the downstream aquatic fauna and reducing the corridor role of rivers for the terrestrial fauna. Those impacts are nevertheless difficult to disentangle as there is no data available to quantify the relative role of each driver. Here we thus considered deforestation as an integrative measure of disturbance, but we also indicated that gold mining represents the main cause of deforestation in the area, and that it generates high pollutant and sediment loads in the aquatic ecosystems. This might explain why slight deforestation (0.67% over the studied area, this is now indicated in the

introduction, line 81) has a so strong effect on the faunas. This is now indicated in the introduction and more clearly discussed in the discussion (lines 158-160 and 166-182). As suggested, we also simplified buffer delineation protocols and no longer detailed model selection protocols in the introduction. Detailed information on these points is in the Methods.

Methods

Line 303: how can samples be retrieved? And how can a fish community be discarded? Clarify

We changed this sentence as: "From the collected eDNA, we amplified DNA from mammals and fish to build species inventories (see Table S6 for details on the number of reads and the number of detected species per sample). For freshwater fish communities, we considered the 64 strictly freshwater sites (Fig. 2). Estuarine areas were not considered for fish because our molecular reference database does not allow the detection of marine and estuarine fish species (lines 444-446).

Moreover, site metadata, including the geographical coordinates of the sites, has been added to the supplementary material (Table S5).

Line 306: DNA samples cannot be 'achieved'; obtained, collected perhaps.

We replaced "eDNA sampling was achieved during the dry season" by "eDNA samples were collected during the dry season" (lines 294-295).

Line 208: after the confusing explanations above, here sampling is explained.

The text does not mention, but the spatial distribution of samples is impressive, as shown in Fig 2. I am still unclear as to what exactly constituted a sampling site and how fish and mammal species were sampled representatively in each site. No details are provided. eDNA of fish is collected in the water, but of mammals, how was it collected? It appears that it was also collected in the water, but there is no discussion of what portion of the mammal community this approach can be expected to reliably sample (all of them, or only aquatic mammals? I don't know…). The methods provide little information on the "ecology" of the work performed and are dominated by explanation of laboratory analyses, making it unclear how the data can be interpreted.

We clarified what a sampling site is and how eDNA was collected on those sites in the "Sampling" section of the methods. As indicated in the methods, a site refers to the locality where eDNA was collected from two replicates of 34 liters of water. In each site, both fish and mammal species were sampled from the water and fish and mammal inventories were obtained from the same water filtration samples. As explained now in the "Biodiversity measures" section of the methods, this sampling protocol provides relevant species inventories for both fish and mammals, and the results obtained from eDNA are comparable to those derived from traditional capture and observation methods such as gillnets for fish[2,3] or observational line transects and camera traps for mammals[4,5].

After four pages of mostly eDNA lab methods, the GIS analyses are explained, and again only the lab part of the work is described with no information describing the system of study, such as vegetation type extent of deforestation, other drivers, spatial configuration of samples in relation to deforestation, etc.

We added a method section titled "Study area" to describe the study system, including vegetation type, information of human occupation and activities, as well as topographical, hydrological and physicochemical variables. All the environmental information is now available in the supplementary material (see Table S5), and the spatial configuration of the sites in relation to deforestation is synthesized in the Figs. 1 and 2. We also extended the "Sampling" section to detail the spatial configuration of the sites in each river (lines 283-288) and the ranges of deforestation among sites are now detailed in the section "Measuring disturbance intensity using GIS data" (lines 432-442).

Results

I see details of landscape measures and species identified by eDNA are given here, but considering

these are not the aims of the ms, I suggest these data should be reported in methods, after all they do not answer the research questions.

Details of detected species with eDNA were moved to the "Biodiversity measures" section of the methods and details on deforestation percentages measured within upstream and upstream-downstream buffers were moved to the "Measuring disturbance intensity using GIS data" section of the methods.

Lines 89-103: interesting results reported here. My only concern is the language used; it focuses on the stats and not on answering the research questions. Referring to R2 as representative of model quality is new to me and an inadequate way of interpreting model performance.

We modified the results to better focus on how statistics answer our research questions. We agree R^2 does not represent model quality but indicates the amount of the response variable variation that is explained by a model. This is now explained in the methods (lines 520-522) and we now refer to the amount of explained variance in the text, on the Fig. 3 and on the supplementary materials.

Lines 104-110 report similar results for functional diversity. Ok. But it is unclear to me why use a taxonomic and a functional approach in the same analysis. Obviously the results were going to be semi-similar. The value of all results so far is being "washed down" by an excessive focus on statistics and not on the research questions. The audience drawn to this ms will seek information on deforestation effects on biodiversity, so focus on describing results should be as such.

Taxonomic and functional diversities would provide the same information if all the species play the same role in ecosystems, which is not true. Recent papers revealed the disproportionate role of some species in ecosystem function (e.g. see Carmona et al.[6]), as well as the differences between functional and taxonomic diversities[7,8].Therefore, taxonomic richness and functional richness represent two key facets of biodiversity and we believe it is important to maintain those two approaches in our paper. We nevertheless agree our results were too technical and we now better focused our results on how the taxonomic and functional results help assessing the deforestation effects on biodiversity.

Lines 111-121 report results that I cannot distinguish from prior results. Again, I am reading lost of statistical details but do not see which research question is being addressed here. As far as I can tell, this paragraph is repeating prior information.

Here we reported biodiversity changes when measuring disturbance intensity independently from the upstream hydrological network (upstream and downstream buffer) and compared these results to those obtained considering deforestation occurring only upstream from the biodiversity sampling sites. It confirmed that biodiversity declines due to deforestation were mediated by hydrology. We now clarified and synthesized this in the results (lines 103-111), and moved all the methodological aspects to the methods.

And again focus on stats is hiding relevance of results reported in lines 122-142. What is the point of this section?

The point of this section (now in lines 112-126) is first to define the optimal spatial extent to predict taxonomic and functional changes due to upstream deforestation, and then to quantify biodiversity changes along deforestation gradients. Particularly, this part allows us to illustrate that slight deforestation (ranging from 0 to 10.6%) resulted in marked decreases for both fish and mammal communities. We here report an average loss of 34% of species richness (26% and 41% for fish and mammals, respectively) and 28% of functional richness (22 and 33% fish and mammals respectively) in deforested areas.
Moran's *I* test was used to test for spatial autocorrelation of our diversity variables to check if there were unforeseen associations between nearby sites. We moved this part to the section

“Species and Functional richness models” of the methods, and better focus on the ecological meaning of our results.

Lines 144-178 report on interesting patterns on functional diversity, but then again focus on stats is hiding the ecological relevance of the research.

Results in this paragraph (now lines 127-146) were modified to highlight the ecological relevance of these results.

Most of the above results are based on the underlying assumption that the observed deforestation is indeed the driving factor behind the fish and mammal patterns observed. Whether that is true has to be put to rest. It could be that fish and mammal species naturally decrease in occurrence downstream, or that other processes or drivers (that are not deforestation) have induced the observed patterns. There may be data that can be used to test if this is true or false, and if the results are indeed due to upstream deforestation.

Yes, you are right. Nevertheless, site position in the upstream-downstream gradient (Strahler order, see Fig. S4), as well as river basin identity were used as integrative descriptors of ecological factors, and those factors were considered as random effects in the models, to properly quantify the influence of human disturbances. Site position in the river gradient is commonly considered as a descriptor of the environmental changes through the upstream-downstream continuum, given that it determines river size and thus the hosting capacity of aquatic species. Basin identity controls for differences in fish compositions and biogeographical processes between river basins. For mammals, we did not control for terrestrial environmental conditions because without human-mediated deforestation, the entire sampled area should be covered by a homogeneous evergreen rainforest. As a consequence, the mammal species distribution range covers the entire study area for all the considered species[9,10]. Moreover, the natural biodiversity pattern in rivers is an increase of fish species diversity along the upstream-downstream course of the river. Such a biodiversity pattern is well known and explained by an increasing availability of habitats and resources from upstream to the downstream parts of rivers[11]. Here we show an opposite pattern - a decline of biodiversity along the upstream-downstream gradient - which is significantly correlated to the deforestation intensity (Fig. S7). Finally, we found a similar correlation between deforestation and mammal biodiversity in spite of the expected homogeneous spatial distribution of mammal biodiversity across the study area.

We also agree that deforestation *per se* is only one disturbance driver among others, but deforestation also represents an integrative measure of human activities, as underlined by Curtis et al.[12]. Here we used deforestation as a proxy of human disturbances including agriculture, mining and urbanization (we clarified this in the introduction, lines 77-78). It would for sure be useful to disentangle the role of the different human drivers, but data on the others drivers are not available. We nevertheless show that gold mining represents the main driver of deforestation and that it is correlated to the other deforestation drivers (Table S4). We agree it was important to specify this, because mining activities generate known disturbances to the aquatic environment, such as water pollution and increases in sediment load, which spread downstream[13,14]. This is now discussed in lines 166-182.

Discussion

The discussion is ok, but I feel it lacks in providing novel insights on the topic largely because of the lack of specificity in framing the research questions and answering them. Given the research topic and lacunae were loosely defined, the discussion is somewhat unfocused. A resharpening of the research framing could substantially improve the contribution of this research.

Large parts of the discussion have been rewritten and reorganised to better focus on the research questions and on the significance of our main findings.

Reviewer #2 (Remarks to the Author)

This manuscript addresses the spatial scale at which human disturbance measured from remotely sensed deforestation relates to fish and mammal species richness and functional diversity surveyed with eDNA. The manuscript will make an important contribution to the scientific literature because the large spatial extent of the eDNA sampling along two rivers in the Amazon is impressive and the results demonstrate the importance of distant disturbance on local fauna. My main concerns are that 1) ecological factors besides disturbance were neither measured nor considered as potential influences on local diversity 2) the introduction as currently written does not sufficiently motivate the study and 3) causality is at times attributed to deforestation despite acknowledgement of additional human disturbances. Careful attention needs to be paid to the use of the terms "deforestation" and "disturbance" which seem to be used interchangeably throughout the manuscript, but which are indeed different (as the manuscript even acknowledges near the end in Lines 421-423: "deforestation intensity around each eDNA sampling site is here considered as a in integrative measure of environmental disturbances including gold-mining, logging, agriculture and human settlements"). A single term that represents what is measured and how it is interpreted should be defined upon first use and then used throughout the manuscript. Specific comments are below.

Thanks for your constructive comments. As detailed below, we took a particular attention to your three main concerns.

1- Ecological factors besides disturbance can indeed affect the distribution of the fauna. The site position in the upstream-downstream gradient (Strahler order), as well as river basin identity were used as integrative descriptors of ecological factors, and those factors were considered as random effects in the models, to properly quantify the influence of human disturbances. Site position in the river gradient is commonly considered as a descriptor of the environmental changes through the upstream-downstream continuum, and basin identity controls for differences in fish compositions and biogeographical processes between river basins. For mammals, we did not control for terrestrial environmental conditions because under no human-mediated deforestation, the entire sampled area should be covered by an homogeneous evergreen rainforest. As a consequence, the mammal species distribution range covers the entire area considered in our study for all the considered species[9,10]. Moreover, the natural biodiversity pattern in rivers is an increase of species diversity along the upstream-downstream course of the river. Such a biodiversity pattern is well known and explained by an increasing availability of habitats and resources from upstream to the downstream parts of rivers[11]. Here we show an opposite pattern - a decline of biodiversity across the upstream-downstream gradient - which is significantly correlated to the deforestation intensity (see Fig. S7). Finally, we found a similar correlation between deforestation and mammal biodiversity in spite of the expected homogeneous spatial distribution of mammals across the study area.

2- The introduction was entirely rewritten according to your suggestions and the ones of Reviewer #1.

3-We now indicate since the introduction that deforestation is considered as an integrative measure of environmental disturbances including gold mining, logging, agriculture and human settlements. Throughout the manuscript we used both "deforestation" and "disturbance" terms. "Disturbance" refers to all human impacts in general (including deforestation) and "deforestation" refers to the integrative variable used for our analyses (the percentage of deforested surfaces). Therefore, when we referred to our results or findings (mostly in results and discussion sections), we used the term deforestation and when we referred to the general implications of human impacts (deforestation as well as other disturbances), we used the word disturbances (mostly in the introduction and sometimes in the discussion for the large implications of our results). We also indicated that deforested surfaces are significantly linked to gold mining activity, which accounts for more than 40% of the deforested surfaces (Table

S4). We agree it was important to specify this, because mining activities generate known disturbances to the aquatic environment such as water pollution and increase in sediment load, which spread downstream[13,14]. This is now discussed.

Introduction

Lines 42-48: These examples are too specific for an opening paragraph that should convey the overarching themes of the paper and knowledge gap that the manuscript addresses

Done. Introduction was rewritten following your comments. We now begin it by the overarching themes (lines 37-49) and then clearly present the knowledge gaps addressed in our paper (lines 51-54, 59-61).

Line 54-55: This statement is tautological

As the introduction changed substantially, the sentence was removed.

Line 49: A focus on riverine ecosystems here does not set the reader up for a manuscript that quantifies mammal species richness and functional diversity. Furthermore, the final sentence of the paragraph and the final sentences of the final introductory paragraph seem to indicate that the manuscript is a methods paper, which is not its main contribution. I suggest moving the final sentence of the introduction to the discussion section and expanding upon the potential applications and extensions of this framework there.

Thank you for these suggestions that improved the introduction considerably. Following your suggestions and the ones of Reviewer #1, the introduction changed substantially to (i) include relevant and pertinent literature, (ii) better convey and identify the knowledge gap of the study and (iii) better explain why human impacts on rivers can affect the surrounding terrestrial diversity. As a consequence, we reduced the methodological part of the introduction to a single sentence, and now focus on the ecological and conservation implications of our work.

Results

Lines 99-103: Additional factors besides deforestation/disturbance might explain these results. For example, if the spatial extent under consideration is larger than the geographic range of some species, then that could influence these results. To what extent might that be a factor in this study? Are there environmental gradients that might influence these results via environmental filtering and species sorting? What about dispersal limitation?

Our entire study area is in a homogeneous climatic and forest context, with the distribution ranges of all the considered mammals spanning over the entire studied area. For fishes, despite slight climate and altitudinal ranges (altitudinal range: 0-157m; temperature range: 25.2-33.4°C; see Table S5 for details), river size increase along the upstream-downstream gradient can lead to changes in species richness and compositions. Similarly, fish are limited in their dispersal between river basins, leading to differences in species composition between the Oyapock and Maroni rivers. These potential environmental effects were considered in the models by including upstream-downstream effect (Strahler order) and river basin identity as random factors. We agree that an in-depth analysis of species assembly rules including species sorting, dispersal limitation and environmental filtering would indeed be of interest, but it exceeds the scope of the present manuscript.

Lines 111-121: It's entirely unclear why upstream rather than downstream would matter for most mammals. Later on in the manuscript there is some mention of water dependent mammals, but nevertheless most mammals would likely be unaffected. The influence of water dependence could be testing by removing the water dependent species and examining the results in their absence. If removing them lowers the explanatory power of the model, then that would bolster the arugment.

The alteration of aquatic ecosystems has been reported to modify the corridor effect of rivers for terrestrial organisms by reducing the movement of terrestrial species, seed dispersal and

pollination services[1,15]. Thus, hydrologically-mediated disturbances are expected to affect terrestrial ecosystems downstream, but not upstream. This is now indicated in the introduction (lines 56-59). We also tested the effect of deforestation independently from hydrological effects by considering deforestation upstream and downstream the biodiversity sampling sites, and the quality of the models drastically decreased (see Fig. S1). We thus concluded that hydrologically-mediated disturbances affect both fish and mammals.
Moreover, we also analyzed the influence of water-dependent species (Giant otter (*Pteronura brasiliensis*), Neotropical otter (*Lontra longicaudis*), Water opossum (*Chironectes minimus*), Capybara (*Hydrochoerus hydrochaeris*) and Lowland tapir (*Tapirus terrestris*)) on the variance explained by the mixed models to understand if water-dependent species drive the observed response to distant upstream deforestation. Comparing a model with all the species to a model excluding water-dependent species is irrelevant since explained variance is dependent on the number of species considered in the models.
We therefore ran a simulation analysis to test the influence of water-dependent mammals on the explained variance retrieved from the mixed models. We compared species assemblages excluding the five water-dependent mammals to 999 sets of species combinations in which five non-aquatic mammals were randomly removed from the sets. Species and functional richness were calculated for each set and implemented in the 14 mixed models considering deforestation at different extents. Then, we calculated the Standardized Effect Size (SES) as $R^2_{withtout\ water\text{-}dependent\ species}$ – mean ($R^2_{random)}$ /sd (R^2_{random}), (sd = standard deviation). SES above or below the [2, -2] interval indicated that water-dependent species contribute significantly more (SES < -2) or not more (SES > 2) to the explained variance of the models than the other species.

For the species richness, as shown on Fig. S3A and C, models remained significant despite a slight decrease of R^2 (0.33 to 0.23 at 30km) when water-dependent species were removed. Fig. S3E indicates that for most extents (except 0.5 to 5km), water-dependent species contributed more to the explained variance of the models than the other species. Fig. S3G demonstrates that regardless of the species removed, models in which upstream deforestation was measured above 5km upstream from the biodiversity sampling sites always remained significant. It thus indicates that, even if the five water-dependent species are more affected by upstream deforestation than the other species, the whole mammal community remains affected by upstream deforestation.

For functional richness, the explained variance drastically decreased when excluding water-dependent mammals and most of the models were not significant (Fig. S3 B, D). However, Fig. S3F demonstrates that for most extents (except 10 to 20km), water-dependent species did not have a higher contribution to the explained variance than the other species. This suggests that the decrease of explained variance is rather due to the removal of five species than the removal of the five water-dependent species. This is also supported by Fig. S3H showing that a substantial part of the p.values are above the significance threshold. Altogether, these panels indicate that there is a set of species with extreme functional strategies (and not restricted to water-dependent species) driving the community response to upstream deforestation.

In conclusion, both water-dependent and terrestrial mammals significantly contribute to the models linking taxonomic and functional biodiversity to upstream deforestation. These additional results are provided in the supplementary Fig. S3 and on the Supplementary note 1.

Lines 133-143: Why turn a continuous variable into a binary category here? Furthermore, since deforestation is considered a composite variable from other types of disturbance too, this seems like an arbitrary cutoff (nor are references provided for the background rate of tree falls). “Pristine” is a misleading term and should be avoided.

We turn our deforestation variable into two categories to be able to functionally characterize the species that are present only in the sites not affected by human-mediated deforestation. We here sought to identify which species - and which corresponding functions - are lost under

human disturbance. We found this binary categorization complementary to the continuous measure given in Fig. 4, as it allows to represent the functional changes between undisturbed and deforested sites, whatever the deforestation level (see Fig. 5).
The background rate of tree fall was measured by randomly selecting 100 places in undisturbed areas (i.e. Core area of the Guianese National Park with no human settlements or activities). Half-circles of 30 km were then generated for each place and the percentage of non-forested surfaces in the GIS layers were calculated. The highest deforestation percentage was then considered as the threshold between deforested and non-deforested sites. This procedure is now detailed in the methods lines 541-549.
We agree that the term "pristine" can be misleading and it was changed to "non-deforested" throughout the manuscript.

Paragraphs starting with Line 144 and Line 160: The final sentences of each of these paragraphs is the key result that should be highlighted in the main text. The other descriptions can be moved to figure legends or supplemental information.

Done. We now focus on the main results and synthesized this information.

For the eDNA results, how far can eDNA travel? How certain are the "absences"? Is there any reason to think that certain taxa or functional groups would be more affected by sampling biases?

A growing body of evidence confirms that eDNA downstream transport is dependent on water flow, and particularly on water velocity. Previous studies demonstrate that water conveys DNA information over short distances in slow flowing rivers (varying from 0.5 to 3 km, e.g. Jane et al.[16]; Civade et al.[17]; Cantera et al.[2]), but probably longer for fast flowing streams (e.g. Deiner et al.[18], Carraro et al.[19]). Given the low slopes of the two studied rivers (0.04 and 0.05% for Oyapock and Maroni, respectively) that correspond to low water velocities, eDNA should not be transported over more than a few hundred meters. This has recently been confirmed for both mammals and fish in the Maroni and Oyapock drainages[3,4]. For those two taxa, eDNA samples allowed to recover the local fauna detected using traditional capture and observational methods, that consist in sampling fishes using gill nets along a 300 to 500m river reach or to observe mammals along a 3 km forest transect. Nevertheless, we cannot exclude that the eDNA of some species, probably the most abundant ones, can be transported over long distances. In the case that this occurs, it would certainly be limited to a few species in our samples as the result of our subsampling analysis considering only distant sites remains consistent with the results of the analysis considering all the sites (see Table S9). This is now detailed in the manuscript.

Absences can never be certain when studying mobile animals in the wild, and our inventories, although not exhaustive, provide a rate of false absences comparable (and even lower) to the standard inventory techniques for mammals and fishes. For instance, for fish, our eDNA protocol provides species inventories equivalent, and even more complete, than the standard sampling protocol used to investigate Neotropical rivers consisting in using 10 nets of 30 metres long on each site[2,3]. Additionally, for fish, we did not identify any difference in detection probability between taxa and functional groups[2,20]. For mammals, comparing line transect observations (successive observations of the fauna along a 3 km long linear track up to 100 km observation per site) to eDNA results shows that eDNA performs better than visual line transects for aquatic and nocturnal mammals, whereas terrestrial mammals were better detected using visual observations. Nevertheless, eDNA and line transect results remained significantly correlated for terrestrial mammals[4].

This is now explained in the "Biodiversity measures" section of the methods and in the last paragraph of the discussion.

Lines 188 & 190: Provide the median alongside the range values.
Done.

Discussion
Lines 195-199: These sentences should be used in the introduction as part of the justification for a composite variable

The introduction and discussion changed substantially. We now explained since the introduction that the percentage of deforested surfaces was used as an integrative measure of human impacts including logging, mining, urban settlements and land conversion to agriculture (lines 76-78), as it has been done in the literature (lines 49-51).

Lines 208-210: This is an overstatement and should be removed or toned down

Done.

Lines 268-273: Do references #43 and #37 provide any insight into additional ecological mechanisms or environmental variables that may influence the results reported in this study? How/in what direction would these limitations influence the results?

Our entire study area is in a homogeneous climatic and forest context, with the distribution range of all the considered mammals spanning over the entire study area. We thus do not expect that additional environmental effects can impact the results. Nevertheless, a distance decay of similarity between faunas can still occur, but this was controlled by including the basin identity and upstream-downstream distance between sites as random variables in the models.
For fishes, despite slight climate and altitudinal ranges (altitudinal range: 0-157m; temperature range: 25.2-33.4°C; see Table S5 for details), environmental conditions change along the upstream-downstream river continuum and can thus lead to changes in species richness and compositions. Moreover, fish are limited in their dispersal between isolated river basins, leading to differences in species composition between river basins. These potential environmental effects have been considered in the models by considering upstream-downstream effect (Strahler order, see Fig. S4) and river basin identity as random factors.

Line 408-416: What was the correlation between the GFC and the gold-mining data?

Correlation coefficients and the p. values between the GFC and the gold mining data are detailed in Table S4 for each spatial extent.

What is the justification for incorporating a separate deforestation layer for 2000?

The Global forest change database provides the deforestation that occurred between 2000 and 2019. Thus, it does not account for the surfaces deforested before 2000. The motivation to incorporate deforestation before 2000 was to get a realistic image of currently deforested areas, including recent and more ancient deforestation.

Lines 494-497: Provide distributions in supplementary material. References for 0.4 as background rate?

The coordinates of each species on the functional space are available for fish and mammal species (Table S3). Moreover, the contribution of each continuous trait to the axes of the functional spaces, the determination coefficient (R^2) of the correlation between each trait and the ordination and the mean position of categorical traits on the two first axes of the PCoA are shown in Table S2. Finally, a species occupancy matrix with read numbers after bioinformatic filtering is available in Table S7 (A) and S7 (B), for fish and mammals, respectively.

To select the appropriate threshold between background rate and human mediated deforestation, we randomly selected 100 random places in non-impacted areas. A half-circle

spatial extent of 30km was then generated for each point (to represent a similar surface to that of the spatial extents delimited for the sampling sites) and the percentages of non-forest surfaces were calculated. The highest deforestation percentage was then considered as the threshold between deforested and non-deforested sites. This is now explained in the methods (lines 541-549). In the initial submission, this threshold was rounded to 0.4%, but now we use the exact value of the threshold (0.33%). Nonetheless, this change did not modify our results.

Reviewer #3 (Remarks to the Author):

Within the last decade, the use of environmental DNA has become a key approach to study the composition and diversity of aquatic ecosystems. More recently, scientists also started to use eDNA to assess how environmental factors, such as contemporary or past land-use, are leaving imprints in ecological communities detectable by environmental DNA. For example, studies have found that certain agricultural practices (assessed by remote sensing) affect the structure and composition of microbial communities assessed through eDNA samples. Similarly, past land-use practices could be reconstructed from eDNA sampled from soil (e.g., Foucher et al. 2020 Scientific Reports). Thereby, it has not only been postulated that eDNA may transform how animal and plant communities are surveyed, but that it also can be used to study large-scale, and possibly cross-ecosystem dynamics. In the current manuscript, eDNA samples taken along two rivers in South America are used to assess the possible impact of deforestation on fish and mammal communities. Fish and mammal communities reconstructed by eDNA were found to show a signal of changed (decreased) diversity when there were even relatively low levels of deforestation upstream.

This is a neat study, with potentially very interesting results, and the manuscript is generally well-written. I especially liked the use of eDNA to address a question of high relevance in conservation biology, namely how much land-use change (or how little) it takes to leave an imprint on organismal communities in whole catchments. Overall, I was satisfied reading the study. However, I also have a series of major points I consider important to be addressed, specifically referring to a) giving more data/results on the sequencing (to clarify if or not some of the patterns observed could be driven by different sequencing platforms used), b) better integration and discussion of the spatial patterns (due to eDNA transport, but also due to null-expectations on distribution of biodiversity in dendritic networks), and c) better integration in the literature on biodiversity patterns expected in such dendritic riverine systems, also differentiating aquatic and terrestrial biodiversity
(fish vs. mammals).

I have relatively extensive comments below, and realise that this will require some work/additional analyses/more data presented in the manuscript. If all my points can be resolved, I think this may become an important study (thus, my extensive comments are truly meant in a supportive manner! I would not comments so extensively if I wouldn't find the manuscript interesting).

Thank you for your encouraging comments and suggestions. We carefully considered all your comments and conducted further analyses. We believe that those complementary results reinforced our conclusions and demonstrated the robustness of our findings.

We added sequencing information, as well as tested the potential effect of sequencing platforms, and no effect was detected. Those results are now added to the supplementary material (Figs. S5 and S6, Supplementary note 2).

Moreover, we considered spatial patterns and conducted analyses to test for spatial bias in our data due to the constrained dendritic nature of rivers. We conducted resampling analyses to select independent (not nested) sites and ran our models on partial data subsets. The results from the partial models, although built using fewer and not nested sites, are consistent with those of the initial models with all the sites, thus demonstrating the robustness of our results.

The DNA transport along rivers was also discussed in more detail using the most recently published papers on eDNA transport in dendritic networks.

Please find below our detailed point by point response

Major comments:

1) The study looked at disturbance intensity (% deforestation) in spatial windows of increasing radius (from 0.5 to 90 km) away from the sampling sites (lines 58-62). Thus, if I understand it correctly, the % of deforested area is calculated for differently sized sections, meaning that the same percent of deforestation can represent very different areas (percent is not based on the same denominator). Comparing percentages with different denominators is generally problematic, but in this case it

assumes a relative (and linear) effect of % area deforested of total area on local communities, and not an absolute area effect. I think this would be something that needs to be shown: is percentage or total area deforested a better predictor, and at what distance? This may be especially relevant for mammals, as the terrestrial area used is their habitat (and thus changes in land cover reduce their habitat, while for fish it is mostly linked to different run-offs and possible changes in
the water chemistry).
Currently, it is only the relative effect of deforestation (lines 88-91), showing that % deforestation predicts fish species richness. While I think this is good, I think it should also be done with absolute values of deforestation.

As we consider distinct extents, the total area deforested is constrained by the extent: in the smaller extents the total surface considered does not exceed a few square kilometers, whereas in the higher extents, the considered surface can reach more than a hundred square kilometers. Moreover, within a given spatial extent the area of the considered upstream river basin can change a lot according to the shape of the river basin. Therefore, the same absolute value of deforested area can represent a marked deforestation for some sites, but a low deforestation for others (in terms of percentage of area deforested). We thus believe it is more relevant to consider the percentage of deforestation than absolute values of deforested surfaces. We nevertheless ran the models using absolute values instead of percentages (results are shown on the figure below). We obtained lower explained variances (R^2) when using absolute values of deforestation compared to the models with percentages of deforested areas (Fig. 3, Table S1). For fish, R^2 values stabilized above 5km with the highest R^2 at 30km but regression slopes remained low, indicating a limited effect of this deforestation measure on biodiversity. For mammals, R^2 values did not stabilize. We therefore preferred to consider percentages of deforested areas instead of the absolute values of deforested areas.

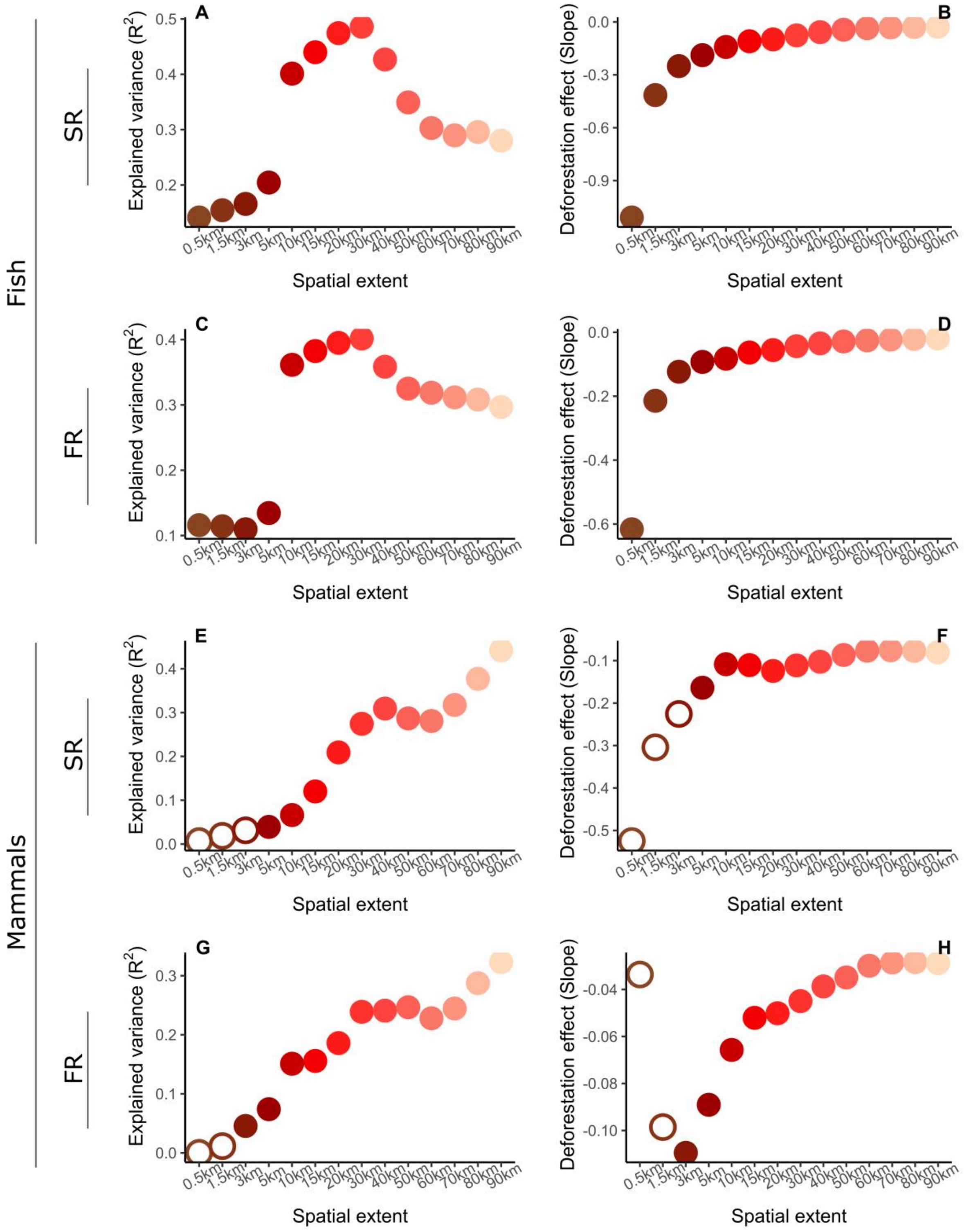

Results of the mixed models relating diversity measures and absolutes values of deforestation upstream from the biodiversity sampling sites for each spatial extent. Left panels represent the explained variance by the mixed models (R^2 value), for each spatial extent. Right panels indicate the strength of the effect of deforestation on biodiversity (slope values of models) for each spatial extent. (**A, B)** Fish species richness (SR) models; (**C, D)** fish functional richness (FR) models; (**E, F)** mammal species richness models; (**G, H)** mammal functional richness models. For each spatial extent, a specific model accounting for site network position and basin identity as random effects was built. Significant models ($p < 0.05$) are indicated by filled circles. Non-significant models ($p > 0.05$) are indicated by open circles. Color shades are consistent with the spatial extent.

2) Spatial autocorrelation of upstream catchment land-use. To my understanding (based on reading the manuscript) it seems that all sites were sampled along the mainstem of two major rivers. This means that sites (or their upstream land-use/upstream catchment) were hierarchically nested. While this is inherent to such a sampling design, it also means that upstream land-use change may affect multiple sites, similarly to a Russian doll. This results in two possible caveats:
Firstly, the same land-use (e.g. patches of deforestation) will be used as a co-variate for multiple sites, which would violate statistical independence (Land-use changes were assessed up to 90 km radius upstream of a sites; based on the information given it seems like sampling sites are often closer to each other than 90 km apart, which would mean that the same land-use change patch would affect multiple downstream sites).
Integrating this hierarchical sampling in an adequate manner is non-trivial (and it also depends on the distances between sites: if land-use effects were only studied as spatial scales much closer to a site than the distance between adjacent sites, this may be neglectable), but should be addressed.

Thank you, this comment is highly relevant. Indeed, all sites were sampled along the main stem of two rivers and our upstream catchments are hierarchically nested. Nevertheless, according to Moran's *I* test our diversity variables were not spatially auto-correlated (lines 513-519).

We also considered directly the potential effects of hierarchical nesting on our results. We conducted additional analyses with site subsamplings to only consider independent sites in our models. Site combinations were created using increasing minimum distances between sites (from 2 to 50km). For each minimum distance, sites that were more distant than the considered minimum distance were randomly drawn from the global set of sites to build a subset of site combinations. This step was repeated 50 times, giving rise to 50 subsets of sites for each minimum distance. The species and functional richness of each site in each subset were then implemented in the 14 mixed models (corresponding to the 14 spatial extents at which deforestation was considered in the study). R^2 and slopes from each model were averaged across the 50 subsets. The site subsampling was conducted separately for each taxon (fish and mammals) and each measure of biodiversity (species richness and functional richness). The subsampling was not done above a minimum distance of 50km between sites because the number of sites separated by more than 50km was not sufficient to run the models. Regardless of the minimal distance between the considered sites, the results remained similar to those obtained with all the sites. Most of the models resulting from subsampling still indicated that the most significant spatial extent to consider human impacts in our study remains between 30 and 40 km from the biodiversity sampling site. Those additional results were added to the supplementary materials (Table S9) and detailed in the methods in lines 529-536.

Secondly, transportation of eDNA along the stream means that the same upstream land-use change may affect multiple downstream patches (with decreasing relevance for further downstream sites, see e.g., Carraro et al. 2020 Nature Communications). This is something that should be addressed in the statistical model and also more extensively discussed.

A growing body of evidence confirms that eDNA downstream transport is dependent on water flow, and particularly on water velocity (and thus the slope of the river). Previous studies demonstrated that water conveys information only over short distances in slow flowing rivers (varying from 0.5 to 3 km, e.g. Jane et al.[16]; Civade et al.[17]; Cantera et al[2].), but probably longer for fast flowing streams (e.g. Deiner et al.[18], Carraro et al.[19]). For instance, Carraro et al.[19] still detected the eDNA signal of species located 30 km upstream from the sampling sites, but along a river with a steep slope (altitude ranges of c.a. 2000 metres over the 30 km river course, average slope 6%). The situation is markedly different for the Guianese rivers, were the altitude range is c.a. 300 metres over the 400 km river courses (average slopes of 0.04 and 0.05% for Oyapock and Maroni, respectively). It is thus difficult to compare Carraro et al.[19] results to ours. Moreover, given the low slope of the two studied rivers, associated with the high water temperature and strong light intensity, eDNA should not be transported over more than a few hundred meters. This has recently been confirmed for both mammals and fish in the Maroni and Oyapock drainages[2–4]. For those two taxa, eDNA samples allowed to recover the local

fauna detected using traditional capture and observational methods, which consist in collecting fishes using gill nets along a river stretch of 300 to 500 m, and observing mammals along a 3 km forest transect.

This is now detailed in the "Biodiversity measures" section of the methods and in the last paragraph of the discussion.

I would also strongly recommend to give a map in the supplement with all sites highlighted and numbered (to link to necessary supplementary data to be added on sequencing and biodiversity results at the site level, see below).

Done, the sites are now numbered in Fig. 2 and can be linked to sequencing data (Table S6), as well as to site characteristics, biodiversity results and upstream deforestation percentages (Table S5). Moreover, the number of reads for each detected species per site is available in Tables S7(A) and S7(B), for fish and mammals, respectively. Fig. 7 displays fish and mammal species and functional richness along the course of the two study rivers.

3) Information on the sites sampled is unclear and insufficient. While it says that in total 74 sites were sampled (lines 295–298), I was not able to find a map in which site id and position could be seen. Maybe I didn't do it correctly, but the CEBA geoportal site given showed me a map with many more sites, and they were also distributed outside these two rivers mentioned.
I would expect a map in the supplement (or even main manuscript) in which all sites are shown (and numbered, to compare to the sequencing data, see comment below), and to give more information on the sites' position, distance and hydrologic variables (mean, sd, min and max distance between adjacent sites, upstream catchment area of each site, etc).

Yes, you are right, the CEBA geoportal shows more sites because in this study we only consider the samples taken in large rivers with a consistent sampling methodology (2 filtrations of 34L of water per site). Stream sites were not considered because it was only possible to consider upstream extents over a few kilometres. To clarify site information, we numbered the sites as you advised (Fig. 2) and removed the access to the geoportal. We now provide all the required information in supplementary materials. Additionally, site information can now be found in Table S5, including upstream deforestation percentages, hydrological and physicochemical variables, as well as distances to the sea and upstream catchment area of each site. Mean, sd, minimum and maximum distances between adjacent sites are detailed in the text (lines 285-288).

From the map I saw it seems that sites were not evenly spaced along the river but distances between adjacent sites varied by at least a factor of 10. Because eDNA is transported (e.g., Pont et al. 2018 Scientific Reports), this may be problematic, and should be discussed.

Indeed, distances between adjacent sites varies from 1 km to 50 km (median= 9 km). This is now detailed in the methods (lines 285-288). As explained above, additional analyses were conducted using site subsampling to only consider independent sites and assess the effects related to the sample spatial distribution. These analyses showed that using only distant sites lead to similar results than those obtained with all sites, suggesting that results were robust and not influenced by the distance between adjacent sites (lines 529-536, Table S9). Moreover, as discussed above, the environmental context (very low topographic slope) do not favor the long transport of DNA, probably explaining the differences between our results and those of Pont et al.[21] for *Coregonus lavaretus*. Moreover, in Pont et al.[21] only *C. lavaretus*, the most abundant species, was detected over long distances and the overall fish assemblage detected using eDNA remained consistent with local fish inventories using capture methods. Thus, in the Maroni and Oyapock rivers, both complementary analyses and previous studies on the same sites support that the heterogeneous distance between our sampling sites may not bias our results.

4) I was impressed by the amount of samples taken and the sequencing conducted. This is indeed a massive study. However, I was also very surprised to see that the sequencing was done on very different platforms. Libraries were sequenced on Illumina HiSeq2500, MiSeq, and NextSeq. I realise that such an extensive sampling involves sequencing multiple libraries. However, different sequencing platforms introduce non-random variation and potentially bias the results, and think here it is very critical to give extensive further information to show if and how data are comparable. Firstly, I would expect a table showing which samples were sequenced on which platforms (it seems that there was no randomization of samples across platforms, which is regrettable; for example, it would be problematic if more downstream sites were sequenced with one platform, and more upstream sites with another platform).

Secondly, it would be necessary to show that the sequencing output (number of reads per sample is comparable across the different platforms (i.e., that there is no effect of sequencing platform on the sequencing output). Thirdly, it would be ideal to have at least some samples spread across platforms, to show that there were no biases in the sequencing (also because different reagent kits were used to do library preparation and the sequencing). The high heterogeneity in sequencing platforms used is a major concern to me, as it may create artifacts in the patterns. For example, if the sequencing of different samples was not randomized but follows the longitudinal sampling along the streams (i.e., downstream samples sampled on one platform, upstream samples on another), effects of position (and thus upstream land-use) may be confounded with the sequencing platform. I realize that it will not be possible to redo the sequencing (and I am for sure not asking for it, because it is a very impressive amount of sequencing done!), but I would expect a thorough analysis showing that there is no artifact patterns created due sequencing platforms. For example, an analysis/figures for the supplement showing the sequencing depth (read numbers), taxa assigned etc. for samples run on the different platforms (can be done in a simple anova, where read numbers, taxa richness etc. is compared against sequencing platform type, or even better, in a lme, where the run identity is even added as further possible confounding factor). This is needed to show that the patterns are not artifacts/driven by the platform type.

Table S6 provides information about the sequencing platforms per sample, the number of raw and assigned reads as well as the number of assigned species.

As the number of samples to analyse and the sequencing depth were important (146 samples and 500 000 reads per sample and per marker), the sequencing platform was opportunistically chosen to fit with SPYGEN production flow. However, to avoid a possible bias linked to the sequencing platform, the samples from the same river were randomly spread across platforms, MiSeq and NextSeq for Maroni river and MiSeq and Hiseq2500 for Oyapock river. Moreover, for some sites, one field replicate was sequenced with one platform and the other with another sequencing platform (Fig. S5). Fig. S5 shows that there is no longitudinal pattern of the sequencing platforms. In addition, samples were tritiated before library preparation to achieve a theoretical sequencing depth of 500 000 per sample and per marker, and the library preparation was the same for all the samples.

Given that samples were assigned to different sequencing platforms, we tested for a potential effect of the sequencing platform on the results. For each site, two filtrations were performed because Cantera et al.[2] showed that there is a variability in the number of detected species between two replicates of the same site sampled at the same moment (called hereafter replicate effect), and therefore that two samples per site are needed to get optimal species inventories. As some of the sites have their two replicates assigned to the same platform while other sites have their two replicates assigned to different platforms (Fig. S5), we distinguished the replicate effect from the platform effect by comparing the difference in species number between site replicates assigned to the same platform (accounting only for replicate effect) to that of the site replicates assigned to different platforms (accounting for replicate and platform effects). If the difference in species number between the site replicates assigned to different

platforms does not differ from that of the site replicates assigned to the same platforms, we can conclude that there is no platform effect.

Because the number of sites with replicates assigned to different platforms is markedly lower than sites with replicates on the same platforms, we randomly selected sites with replicates on the same platforms to reach the same number of site that the number of sites with replicates on different platforms. Then, for each site, we calculated the difference in species number between the two replicates and conducted a Mann-Whitney U test to compare the difference in species number between site replicates assigned to the same platforms to that of the site replicates assigned to different platforms. We bootstrapped the procedure 50 times (with different set of sites) and displayed the distribution of p.values (Fig. S6). Analyses were conducted separately for the Maroni and Oyapock and for both taxa because different platforms were used.

There was no significant difference between the number of species detected in sites with replicates assigned to a single platform and sites with replicates assigned to different platforms, we thus concluded there is no significant effect of the sequencing platform on the biodiversity measures. This is now detailed in the methods and in Fig. S6.

5) Do I read it correctly that in total fourteen negative controls and four negative PCR controls were used only? Given that there were in total 18 libraries sequenced (10 fish, 8 mammals), this mean that there was only one or two negative control in each of these libraries? While I personally do not think that negative controls can tell us everything about the quality and feasibility of a sequencing run, I still think this is at the very low (too low?) end. I realise that it will not be possible to change. However, I would firstly expect that the results of negative controls (number of reads, possible contaminations etc) are shown in more detail (table), and secondly that this low number of controls is discussed. It is a bit regrettable that in such a massive and really impressive effort of sequencing there was only little attention given to the negative controls.

This was a typo error and we corrected this mistake, there were fourteen negative PCR controls. However, once we counted again the number of negative controls, we found 13 negative extraction controls instead of 14, with one amplified twice for the teleo primer, for this reason we counted 14 the first time. We corrected this mistake. For the fourteen negative PCR controls that were amplified, eight were amplified with teleo primers and six for Vert01 primers. It should be also noticed that all the negative controls were amplified 12 times per marker, that means that for the negative extraction controls there were 168 amplifications with teleo primers (13 negative controls, with one amplified and sequenced twice) and 156 amplifications with Vert01 primers (13 negative controls). For the PCR negative controls, there were 96 amplifications with teleo primer and 72 for Vert01 primer. We now explain better this in lines 369-378. Sequencing information on controls can be found in Table S6C.

After the comparison with the reference DNA database for fish and vertebrates, all the negative controls were negatives. The negative controls were checked for other species detection and only human and domestic animals (chicken, turkey, pig, dog and cat) were detected.

6) I was missing all relevant and necessary information on the sequencing results. Specifically, I was missing tables (in the supplement) giving raw read numbers, read numbers after the most important bioinformatic steps, species richness numbers etc. per site (for example, in results lines 77-80, I would expect a table in the supplement that gives for each site at least the number of fish, or even better, the identity of fish. A simple site by species occupancy matrix for the supplement would do this job, and should be the standard for such studies). Now, this information is only given in a very concerted manner (mean across all sites). However, understanding the plausibility of such datasets crucially depends on a balanced sequencing depth, and a good understanding of the variation in sequencing

output across different sites. To me, such tables synthesizing the raw data at the per site-level are essential.

We added Table S6, which contains sequencing information for each sample (two samples were collected per site). This supplementary table also provides the number of detected species, the number of sequences before and after the bioinformatic analysis, as well as in which type of run the controls were sequenced. It is not possible to give the information about the number of sequences in the intermediary steps as the pipeline is concatenated with several pipes. Biodiversity measures (species and functional richness) per site are indicated in Table S5. Moreover, a species occupancy matrix with read numbers after bioinformatic filtering is available in Table S7 (A) and S7 (B), for fish and mammals, respectively.

Reviewer #1 suggested moving the results in Lines 77-80 to the materials and methods section, so we moved this part and refer to the new added data for more details (lines 454-461).

7) The study compares position/extent of upstream land-use change to changes in communities assessed per site downstream. There is two possible explanations on how such a pattern emerges: Firstly, all the upstream changes together shape the downstream community assessed. Secondly, communities in the river are shaped along the stream based on adjacent land-use, and this signal is then (through transport of eDNA) integrated over space and collected in one sample. Based on knowledge of eDNA transport in streams, the second seems much more plausible to me (and I also think that this is how the authors see it). Importantly, however, such a spatial integration also directly implies that distance and position of all upstream sources (of eDNA) will together shape the locally assessed signal. Recent work (e.g. Carraro et al. 2020 Nature Communications) showed how hydrologic models can be used to adequately reconstruct eDNA input and transportation, and thus to better link the eDNA signal to upstream land-use type (or land-use change). While it may go beyond the scope of this manuscript to integrate such a modelling approach, I think the hierarchic structure of their sampling design would at least require a discussion on hydrologic transport, and possible effects on the eDNA signal.

Given the eDNA transport in rivers, biodiversity responses of deforestation can indeed be spatially integrated, but as indicated in point 2, eDNA of the local faunal community should not be transported over more than a few hundred meters in our sampling sites, given the low slope of the rivers (0.04 and 0.05% for Oyapock and Maroni, respectively). This is supported by recent papers on the same rivers showing that both fish and mammals inventories using eDNA provide a local image of the fauna comparable to that obtained using capture and observational methods[3,4]. Nevertheless, we cannot exclude that the eDNA of some species, probably the most abundant ones, can be transported over long distances. In the case that this occurs, it would certainly be limited to a few species in our samples as the result of our subsampling analysis considering only distant sites remains consistent with the results of the analysis considering all the sites. Using hydrologic models to reconstruct eDNA input and transportation therefore goes beyond the scope of the manuscript, even though it represents an interesting way to reconstruct local faunas when eDNA is transported over long distances. We thus added a discussion on the potential downstream transport of eDNA and underlined the interest of hydrologic models in the last paragraph of the discussion.

8) The finding that fish community richness was affected by deforestation is very interesting, and the explanation on a decrease due to loss of species (and not turn-over; lines 151-157) is plausible. However, richness of fish (and other organisms) in dendritic networks is not random. Generally, richness is highest at downstream sites, both due to the habitat-size effect of large rivers, as well as a better connectivity. A decrease of richness with upstream positions is rather universal in fish. Importantly, anthropogenic effects in/along riverine networks are also not random, and often stronger at more downstream/lowland positions. Disentangling this would be interesting, and I see two opportunities with this dataset: firstly, it would be interesting to analyse richness with position in the riverine network (there is extensive reviews and opinion papers describing expectations on biodiversity patterns in riverine networks). Secondly, such an analysis could also include null models: if richness is (was) higher in pristine downstream sites, and if these are more affected by human activities, then the effect of deforestation could even be stronger (as they may spatially coincide). Discussing possible

null-model expectations or, even better but maybe a bit too much for the manuscript, running a neutral meta-community model to create null-model expectations on richness across the network and then map observed diversity and changes thereof would add a neat angle to the manuscript, such that it would be well-embedded in the literature on biodiversity patterns in dendritic metacommunities.

Indeed, fish diversity patterns are not random along dendritic networks (this is now detailed in 268-273). The natural biodiversity pattern in rivers is an increase of species diversity along the upstream-downstream course of the river. Such a biodiversity pattern is well known and explained by an increasing availability of habitats and resources from the upstream to the downstream parts of rivers[11]. Here we show an opposite pattern - a decline of biodiversity along the upstream-downstream gradient - which is significantly correlated to the deforestation intensity (see Fig. S7). Moreover, for fish, we observed distinct upstream-downstream species richness gradients between rivers. In the Maroni river, fish species richness decreased along the upstream downstream gradient, which correlates with the intensity of deforestation. In contrast, in the Oyapock river the species richness as well as the deforestation intensity were more homogeneously distributed along the upstream-downstream gradient. For mammals, we also found a correlation between deforestation and biodiversity in spite of the expected homogeneous spatial distribution of mammal species across the study area. Those biodiversity patterns are now shown in Fig S7.

Furthermore, we controlled for potential environmental effects by considering the site position in the upstream-downstream gradient (Strahler order), and river basin identity as random effects in the models. Site position in the river gradient is commonly considered as a descriptor of the environmental changes through the upstream-downstream continuum, and basin identity controls for differences in species compositions and biogeographical processes between river basins that could affect both fish and mammals (although the species distribution range of mammals covers the entire area considered in our study)[9,10].

Minor comments:
9) I recommend to give not only the names of primer used, but to actually give the primer sequence (this can also be done in the supplement). There is many, even closely similar, primers used to detect fish, and giving the nucleotide base sequence would be helpful and is a good practice.

The primers sequences are now integrated in the main text (lines 325-335).

10) Lines 113-114: What is the effect of disturbance (deforestation) on local diversity vs. a change due to transportation of eDNA?

We are not sure to understand your question, but as explained above, our complementary analyses show that eDNA transportation did not (of hardly) affected our results.

11) Deforestation and other land-use changes are often associated with increasing run-offs of humic acids into the rivers. These humic acids are very well known inhibitors in PCR reactions (Uchii et al. 2019 Environmental DNA) and thus may directly affect sequencing output. Are there any data available on water quality/water chemistry, teasing apart effects of land-use change on biodiversity vs. simply detecting less biodiversity due to effects of land-use change on water chemistry interfering with PCR?

Although no data is available on humic acids, higher humic acids concentration in deforested areas would probably lower the pH of the water. We thus tested if pH differs between deforested and non-deforested sites, and found no significant difference (Kruskal-Wallis test, p-value = 0.921). We also tested water conductivity and turbidity, and again, no significant difference was found between deforested and non-deforested sites ((Kruskal-Wallis test, p-value = 0.382 and 0.925 for conductivity and turbidity, respectively). It should also be noted that after the DNA extraction, all the samples were tested for inhibition and treated accordingly (lines 320-324). Moreover, even if we cannot exclude an effect of water chemistry on species

detections, it remains quite unlikely given the slight variation of water chemistry along the two river courses (see Table S5).

References

1. Leal, C. G. *et al.* Integrated terrestrial-freshwater planning doubles conservation of tropical aquatic species. *Science (80-.).* **370**, (2020).
2. Cantera, I. *et al.* Optimizing environmental DNA sampling effort for fish inventories in tropical streams and rivers. *Sci. Rep.* **9**, 1–11 (2019).
3. Cantera, I. *et al.* Characterizing the spatial signal of environmental DNA in river systems using a community ecology approach. *Mol. Ecol. Resour.* (2021). doi:10.1111/1755-0998.13544
4. Coutant, O. *et al.* Amazonian mammal monitoring using aquatic environmental DNA. *Mol. Ecol. Resour.* 1–14 (2021). doi:10.1111/1755-0998.13393
5. Mena, J. L. *et al.* Environmental DNA metabarcoding as a useful tool for evaluating terrestrial mammal diversity in tropical forests. *Ecol. Appl.* **31**, e02335 (2021).
6. Carmona, C. P. *et al.* Erosion of global functional diversity across the tree of life. *Sci. Adv.* **7**, 1–13 (2021).
7. Su, G. *et al.* Human impacts on global freshwater fish biodiversity. *Science (80-.).* **371**, 835–838 (2021).
8. Toussaint, A. *et al.* Non-native species led to marked shifts in functional diversity of the world freshwater fish faunas. *Ecol. Lett.* **21**, 1649–1659 (2018).
9. Emmons, L. & Feer, F. *Neotropical rainforest mammals: a field guide*. (Chicago: University of Chicago Press., 1997).
10. Hansen, E., Richard-Hansen, C., Dewynter, M., Pourcher, C. & Soissons-Tairraz, C. *Faune de Guyane : Guide des principales espèces soumises à réglementation*. (2000).
11. Allan, J. D. & Castillo, M. M. *Stream Ecology*. *Stream Ecology* (Springer Netherlands, 2007). doi:10.1007/978-1-4020-5583-6
12. Curtis, P. G., Slay, C. M., Harris, N. L., Tyukavina, A. & Hansen, M. C. Classifying drivers of global forest loss. *Science (80-.).* **361**, 1108–1111 (2018).
13. Hammond, D. S., Gond, V., De Thoisy, B., Forget, P. M. & DeDijn, B. P. E. Causes and consequences of a tropical forest gold rush in the Guiana Shield, South America. *Ambio* **36**, 661–670 (2007).
14. Gallay, M. *et al.* Impact of land degradation from mining activities on the sediment fluxes in two large rivers of French Guiana. *L. Degrad. Dev.* **29**, 4323–4336 (2018).
15. Tewksbury, J. J. *et al.* Corridors affect plants, animals, and their interactions in fragmented landscapes. *Proc. Natl. Acad. Sci. U. S. A.* **99**, 12923–12926 (2002).
16. Jane, S. F. *et al.* Distance, flow and PCR inhibition: eDNA dynamics in two headwater streams. *Mol. Ecol. Resour.* **15**, 216–227 (2015).
17. Civade, R. *et al.* Spatial Representativeness of Environmental DNA Metabarcoding Signal for Fish Biodiversity Assessment in a Natural Freshwater System. *PLoS One* **11**, 1–19 (2016).
18. Deiner, K., Fronhofer, E. A., Mächler, E., Walser, J. C. & Altermatt, F. Environmental DNA reveals that rivers are conveyer belts of biodiversity information. *Nat. Commun.* **7**, (2016).

19. Carraro, L., Mächler, E., Wüthrich, R. & Altermatt, F. Environmental DNA allows upscaling spatial patterns of biodiversity in freshwater ecosystems. *Nat. Commun.* **11**, 1–12 (2020).

20. Cilleros, K. *et al.* Unlocking biodiversity and conservation studies in high-diversity environments using environmental DNA (eDNA): A test with Guianese freshwater fishes. *Mol. Ecol. Resour.* **19**, 27–46 (2019).

21. Pont, D. *et al.* Environmental DNA reveals quantitative patterns of fish biodiversity in large rivers despite its downstream transportation. *Sci. Rep.* **8**, (2018).

Reviewers' Comments:

Reviewer #1:
Remarks to the Author:
I have reviewed ms NCOMMS-21-20430A, which uses eDNA to assess the spatial extent of deforestation effects on fish and mammal diversity in two watersheds of Amazonia. I am a fish and fisheries ecologist so I am unable to assess the soundness of the genetic analyses; I trust other reviewers are evaluating that critical aspect of the study. As outlined in my first review of this ms, I think it is great study with potentially transformative implications. The authors show that local-level deforestation affects fish and mammal diversity at (much) larger spatial scales and that these effects of biodiversity are mediated by river hydrology. I have no problems with the analyses and results presented. However, while I see this version of the ms as having improved over the first, I feel it is not ready for acceptance yet due to substantial issues in the presentation of the research. Such issues of presentation are too many to allow me to provide here a detailed, point-by-point critique, which gives me no option other than describing the issues in general terms and hope they will make sense to the authors.

1. English — the writing could be substantially improved everywhere. There are minor issues with grammar and major issues with overall clarity; the writing could be more concise and direct. It feels like it rambles at times.

2. Clarity of main points — This problem stems in part from 1, but also from what I can only imagine is a general lack of clarity on the authors' minds as to what exactly are the conceptual contributions of this study. Take the introduction as an example. It is mainly composed of a single paragraph that is 1.5 pages long (huge!). This paragraph starts off establishing that biodiversity is on the decline and few protections exit for freshwater ecosystems, which are susceptible to hydrologically-mediated processes that can disperse and even amplify human impacts. This paragraph then continues on establishing that there is a need to develop frameworks to assess deforestation effects on rivers, particularly in tropical forests such as the Amazon. While all of this makes sense, I would like to encourage The authors to: (i) more clearly organize the ideas, and (ii) more clearly identify exactly what their study does that no study has yet done. While I think I understand the general framing of the study, it is not clear, to me at least.
This problem of lack of clarity affects the majority of the main text, including sections of methods, results and discussion. The paragraph in the results section, in addition to describing poorly what the technical findings are, it fails at linking the results to the larger research questions addressed by the study. The text in results often feels as it is rather disconnected from the mains story the authors want to tell. I know this journal has methods at the end, but results refer to "models" that have not yet been (briefly) explained. Description of results of Fig 5 was completely un-understandable for me, and most other results were fuzzy for me.
Similar to my critique of the problem of presentation of ideas in the introduction, I feel the

discussion needs major revisions to again make clear what is being said and what the real contribution of this study is. All content there makes sense to me, but it is not clear. In doing this, I also think it is very important that the conceptual contribution of the study to conservation be much more clearly identified. Currently, the text states the need for more integrative and comprehensive conservation and highlights the importance of hydrological-connectivity in dispersing human impacts. Ok. But could specific leading conservation programs be improved based on results of this study; please identify which ones and how. What conservation paradigm could be improved by these results?
I know I said earlier the authors could emphasize the usefulness of eDNA, and they right-fully did it, but I feel that section in discussion is too long. In line with my comments on clarity, that section could be much more concise and straighter to the point, but currently it is over one full page long and in the same "closing" paragraph that is 2.5 pages long, which is more than huge.
The abstract also needs work.

Reviewer #2:
Remarks to the Author:
The authors have done a nice job incorporating comments and have significantly improved the manuscript. I have only a few points that need further clarification or modification.

1) The title directly attributes causality to deforestation despite the fact that the manuscript acknowledges multiple mechanisms by which species richness and taxonomic diversity may be negatively impacted. I therefore recommend revision of the title accordingly.

2) The manuscript repeatedly uses the term "hydrologically-mediated effects" but this term is not currently defined. Please define clearly at first use.

3) In line 110 should R2 be p?

4) In line 182 the term "corridor effect" is used but is not defined. Please clarify. As a tropical terrestrial mammal ecologist, it is unclear to me what precisely a "hydrologically-mediated effect of deforestation on terrestrial mammal diversity". My best guess in this case is that manuscript is suggesting that upstream deforestation may cause terrestrial mammals to reduce their use of the river as a corridor for movement, but with the exception of few mammals that may do this (e.g., the jaguar), I am unconvinced by this as a plausible mechanism. Given what we know about threats to tropical mammals from hunting (e.g. see work by Benita-Lopez), increased poaching seems likely to have a bigger impact

5) Lines 139-144 See also recent paper by Gorczynski et al (2021 Proc B) documenting declines in tropical mammal functional diversity due to anthropogenic disturbance

6) Line 217 – the claim that the responses by fish and mammals are due to the "same impact"

is problematic because the mechanisms for the diversity declines are 1) unmeasured and 2) likely different as described in the manuscript

Reviewer #3:
Remarks to the Author:
I would like to thank the authors for their revision, and I am generally satisfied and my most important comments could be clarified. This is an interesting manuscript, and I think will be of importance to the field. However, while I am satisfied with the answers in the response letter, I have three remaining comments that I would like to see resolved before the manuscript can be recommended for acceptance from my side. I think all of them are doable, and they mostly refer to an integration of what has been answered in the response letter to be actually complemented with corresponding changes in the manuscript.

Specifically, the integration of discussions on dendritic networks, and also the use of not only the proportion but also total area of land-use change affecting the findings is not yet integrated sufficiently in the manuscript. I am fine with the arguments provided in the response letter, but would like to see this also being change and integrated in the manuscript. For the former, the discussion of recent literature on effects of dendritic networks structure, and particularly disturbances therein, on diversity is well studied in the scientific literature (in work between 2010–2020, yet the manuscript still mostly refers to literature before 2010). For the latter, I would expect the alternative analysis being given at least in the supplement, and in the main part being specifically mentioned that effects sizes and R2 are much smaller when actual disturbed area, and not only % disturbed area, are used.

Thus, I suggest to implement the following two changes in the ms:

1) The discussion on diversity patterns in dendritic networks is still rather sparse, and not very contemporary, most of this research has been post-2010, yet for example, the manuscript mostly refers to a text book by Allan (2007), which is a more generic reference on riverine systems, yet most theoretical work on dendritic networks and biodiversity patterns therein is from thereafter (e.g., Muneepeerakul et al. 2008 Nature, or Altermatt 2013 for a review).
Importantly, there are even specific models that predict that the expected diversity patterns are inverse when there is disturbances (see Harvey et al. 2018 for models and predictions under undisturbed and disturbed scenario), which would be very much in line with what the authors found here. I would strongly recommend to consider this aspect, as it will allow a much better embedding of the interesting findings in the context of the extensive work on dendritic networks (Lines 268–273 and elsewhere). Specifically, the inverse gradient of richness with respect to network position (Fig. S7) is predicted by Harvey et al. 2018 under disturbances in a theoretical model, thus would actually very much support the empirical findings presented here (this may even be among the first empirical demonstrations thereof, a nice point to add).

Muneepeerakul et al. (2008) Neutral metacommunity models predict fish diversity patterns in Mississippi-Missouri basin. Nature, 453(7192): 220-222.

Altermatt F (2013) Diversity in riverine metacommunities: a network perspective Aquatic Ecology, 47: 365-377.

Harvey et al. (2018) Disturbance reverses classic biodiversity predictions in river-like landscapes. Proceeding of the Royal Society B-Biological Sciences, 285: 20182441.

2) Further, I found that the answer to my previous comments 1 (I was reviewer #3 in the previous round) was good in the response letter, however, I would expect that this additional analysis and information is also given in the manuscript, which was not the case. I had asked what the effects are when there is not % but absolute area deforested looked at. Indeed, there is a significant difference in the findings with the absolute values having much lower R2, and not stabilizing than when using % of land-change (detailed by the authors in the response letter). I think this is highly relevant, yet not clearly stated in the manuscript. I respect the authors decision to stick with the percentage as their main finding, but would expect that in the main manuscript this additional analysis (with absolute area, instead of % used) is mentioned, saying that it gives a different result (these results and figures, as they are now given in the response letter, should then be added to the supplement. I was a bit surprised to see these additional analyses and their discussion in the response letter, yet not being mentioned in the main manuscript and/or supplement. Thus, I would expect that next to Figure 3 given in the main ms, the paralleling figure given as a response to my comment 1 in the response letter is also added to the manuscript (supplement), and referred to in the main text.

3) as a minor comment: the three sequencing platforms used are not "randomly distributed along the river courses" (line 372, see also Fig. S5), there was two platforms used for one stream (MiSeq and NextSeq), on two platforms used for another stream (Hiseq_2500 and Miseq). Thus, there was a very strong blocking (i.e., non-independence) in platforms used between the two streams. This is ok, but should be mentioned specifically. Saying "randomly" would be inadequate.

Again, I would like to congratulate the authors for their interesting manuscript, and I am looking forward to see the remaining comments being resolved.

REVIEWER COMMENTS

Reviewer #1 (Remarks to the Author):

I have reviewed ms NCOMMS-21-20430A, which uses eDNA to assess the spatial extent of deforestation effects on fish and mammal diversity in two watersheds of Amazonia. I am a fish and fisheries ecologist so I am unable to assess the soundness of the genetic analyses; I trust other reviewers are evaluating that critical aspect of the study. As outlined in my first review of this ms, I think it is great study with potentially transformative implications. The authors show that local-level deforestation affects fish and mammal diversity at (much) larger spatial scales and that these effects of biodiversity are mediated by river hydrology. I have no problems with the analyses and results presented. However, while I see this version of the ms as having improved over the first, I feel it is not ready for acceptance yet due to substantial issues in the presentation of the research. Such issues of presentation are too many to allow me to provide here a detailed, point-by-point critique, which gives me no option other than describing the issues in general terms and hope they will make sense to the authors.

Thank you for your detailed comments that were all carefully considered. We believe that the clarity of our manuscript greatly benefitted from the changes we made following your comments. Please see below our detailed point-by-point response.

1. English — the writing could be substantially improved everywhere. There are minor issues with grammar and major issues with overall clarity; the writing could be more concise and direct. It feels like it rambles at times.

We took a particular attention to manuscript clarity and thus reorganized large parts of the introduction, results and discussion. We substantially modified these parts to make them more concise and direct.

English has now been checked by Wiley professional editing services to avoid spelling and grammatical errors. We used the advanced editing option proposed by Wiley that checks for manuscript clarity, which further enhanced the clarity of the main text.

Finally, we removed all the redundancies. All of the changes permitted to reduce the length of the manuscript by 210 words.

2. Clarity of main points — This problem stems in part from 1, but also from what I can only imagine is a general lack of clarity on the authors' minds as to what exactly are the conceptual contributions of this study. Take the introduction as an example. It is mainly composed of a single paragraph that is 1.5 pages long (huge!). This paragraph starts off establishing that biodiversity is on the decline and few protections exit for freshwater ecosystems, which are susceptible to hydrologically-mediated processes that can disperse and even amplify human impacts. This paragraph then continues on establishing that there is a need to develop frameworks to assess deforestation effects on rivers, particularly in tropical forests such as the Amazon. While all of this makes sense, I would like to encourage The authors to: (i) more clearly organize the ideas, and (ii) more clearly identify exactly what their study does that no study has yet done. While I think I understand the general framing of the study, it is not clear, to me at least.

We have modified the introduction in order organize the ideas to more clearly and to define the conceptual contributions of the study, which are i) to measure how vertebrate biodiversity, surveyed by environmental DNA collected in two Amazonian rivers, is affected by disturbances located from the immediate vicinity of the biodiversity sampling sites to up to 90 km upstream; and ii) to test if fish and mammals respond in the same way to these remote disturbances. This is now clarified in the introduction and the knowledge gaps filled by our study are now clearly identified (lines 51-53 and 60-63). We also reorganized the introduction in distinct.

This problem of lack of clarity affects the majority of the main text, including sections of methods, results and discussion. The paragraph in the results section, in addition to describing poorly what the technical findings are, it fails at linking the results to the larger research questions addressed by the study. The text in results often feels as it is rather disconnected from the mains story the authors want to tell. I know this journal has methods at the end, but results refer to "models" that have not yet been

(briefly) explained. Description of results of Fig 5 was completely un-understandable for me, and most other results were fuzzy for me.

We rewrite parts of the results section to better link the results to the research questions addressed in the study and took a particular attention to make the results understandable without reading the methods. We for instance no longer refer to “the models”. Instead, we briefly explained we used linear mixed models to measure the relationship between biodiversity and deforestation (lines 95-101). We did the same for the analysis of functional structure, which is now more clearly introduced by explaining that the life history and morphological traits of the species were resumed using a principal coordinates analysis (lines 122-142). We also detailed and simplified the legend of Figure 5 to make it clearer.

Similar to my critique of the problem of presentation of ideas in the introduction, I feel the discussion needs major revisions to again make clear what is being said and what the real contribution of this study is. All content there makes sense to me, but it is not clear. In doing this, I also think it is very important that the conceptual contribution of the study to conservation be much more clearly identified. Currently, the text states the need for more integrative and comprehensive conservation and. Ok. But could specific leading conservation programs be improved based on results of this study; please identify which ones and how. What conservation paradigm could be improved by these results?

The discussion was revised to clarify the contribution of the study in the field of ecology but also for conservation applications. We deeply reorganized the discussion to avoid redundancies and make it more focused on the conceptual contributions of our study. We also detailed the practical implications of our study for conservation and illustrated it by discussing how our framework could be used to better define IUCN protected areas, and thus more efficiently evaluate the wilderness areas of the globe. Notably, we highlighted the importance of considering hydrologic connectivity into the evaluation of the wilderness of protected areas to ensure their sustainability (Lines 209-215).

I know I said earlier the authors could emphasize the usefulness of eDNA, and they right-fully did it, but I feel that section in discussion is too long. In line with my comments on clarity, that section could be much more concise and straighter to the point, but currently it is over one full page long and in the same "closing” paragraph that is 2.5 pages long, which is more than huge.

This 2.5 pages long paragraph was indeed discussing conservation implications and methodological issues. We split it in two distinct paragraphs, one related to the conservation implications of considering hydrologic connectivity, and the other discussing the usefulness of eDNA to inventory both aquatic and terrestrial fauna. This last part has been clarified, but it is still one page long because on the first revision round, Reviewer 3 asked for a detailed discussion on methodological issues concerning DNA transport in rivers, and about the consideration of the dendritic structure of rivers. We therefore maintained a quite extended discussion on these points.

The abstract also needs work.
The abstract has been revised to make it clearer, while not exceeding the maximal word count requested by the journal (150 words). It also benefitted from Wiley English language editing services.

Reviewer #2 (Remarks to the Author):

The authors have done a nice job incorporating comments and have significantly improved the manuscript. I have only a few points that need further clarification or modification.

Thanks for these encouraging comments.

1) The title directly attributes causality to deforestation despite the fact that the manuscript acknowledges multiple mechanisms by which species richness and taxonomic diversity may be negatively impacted. I therefore recommend revision of the title accordingly.

You are right, Deforestation is here used as a proxy of anthropization, we therefore changed the title as "Low level of anthropization causes harsh biodiversity decline in Amazonia."

2) The manuscript repeatedly uses the term "hydrologically-mediated effects" but this term is not currently defined. Please define clearly at first use.

We now refer to the term "hydrologic connectivity", which is more commonly used in the literature. We defined this term as the "the water-mediated transfer of matter, energy, and/or organisms within or between elements of the hydrologic cycle" the first time we used it in the Introduction (lines 44-46), and in the Methods (lines 390-391). This definition was initially proposed by Pringle 2001 (Ref. 9) and we therefore refer to this paper.

3) In line 110 should R2 be p?

Yes, you are right. This error was corrected.

4) In line 182 the term "corridor effect" is used but is not defined. Please clarify. As a tropical terrestrial mammal ecologist, it is unclear to me what precisely a "hydrologically-mediated effect of deforestation on terrestrial mammal diversity". My best guess in this case is that manuscript is suggesting that upstream deforestation may cause terrestrial mammals to reduce their use of the river as a corridor for movement, but with the exception of few mammals that may do this (e.g., the jaguar), I am unconvinced by this as a plausible mechanism. Given what we know about threats to tropical mammals from hunting (e.g. see work by Benita-Lopez), increased poaching seems likely to have a bigger impact

Our sentence was indeed unclear. We ought to explain that riparian vegetation can be altered by upstream disturbances mediated by water because of the tight codependency of riparian vegetation and freshwater ecosystems. These alterations may in turn affect terrestrial biodiversity. Moreover, we added in the explanation the potential effects of hunting. We reformulated this as follows "Those results also hold when considering only terrestrial mammals (Fig. S2, Note S1). The spatially extended effects of anthropisation on terrestrial mammal diversity may be explained by the upstream degradation of riparian vegetation. Through hydrologic connectivity, anthropisation affects the structural dynamics of water flow and physico-chemistry as well as aquatic habitats at the catchment scale[30]. Alterations in freshwater ecosystems may, in turn, affect the downstream structure and composition of the riparian vegetation and the terrestrial biodiversity it supports[30]. Moreover, human settlements established in remote areas in response to the development of small-scale gold mining lead to subsistence hunting along the rivers. This practice also constitutes a non-negligible disturbance to terrestrial fauna[31]". (Lines 166-75).

5) Lines 139-144 See also recent paper by Gorczynski et al (2021 Proc B) documenting declines in tropical mammal functional diversity due to anthropogenic disturbance

Thank you for this relevant reference. We added this reference to support our results, notably the loss of top predators in mammal communities subjected to disturbances (Lines 189-191).

6) Line 217 – the claim that the responses by fish and mammals are due to the "same impact" is problematic because the mechanisms for the diversity declines are 1) unmeasured and 2) likely different as described in the manuscript

Yes, you are right our sentence was misleading. We meant that both taxa show the strongest response to deforestation for the same spatial extent at which deforestation is considered. This indeed does not mean that the same mechanisms are responsible to the declines in fish and mammal diversity. We therefore modified this sentence as follows: "Here, both terrestrial and aquatic fauna responded similarly to deforestation over a spatial extent of 30 km upstream from the sampling sites. Thus, cross-ecosystem conservation designs should be beneficial to both terrestrial and aquatic fauna". (Lines 217-219).

Reviewer #3 (Remarks to the Author):

I would like to thank the authors for their revision, and I am generally satisfied and my most important comments could be clarified. This is an interesting manuscript, and I think will be of importance to the field. However, while I am satisfied with the answers in the response letter, I have three remaining comments that I would like to see resolved before the manuscript can be recommended for acceptance from my side. I think all of them are doable, and they mostly refer to an integration of what has been answered in the response letter to be actually complemented with corresponding changes in the manuscript.

Thank you for your encouraging comments

Specifically, the integration of discussions on dendritic networks, and also the use of not only the proportion but also total area of land-use change affecting the findings is not yet integrated sufficiently in the manuscript. I am fine with the arguments provided in the response letter, but would like to see this also being change and integrated in the manuscript. For the former, the discussion of recent literature on effects of dendritic networks structure, and particularly disturbances therein, on diversity is well studied in the scientific literature (in work between 2010–2020, yet the manuscript still mostly refers to literature before 2010). For the latter, I would expect the alternative analysis being given at least in the supplement, and in the main part being specifically mentioned that effects sizes and R2 are much smaller when actual disturbed area, and not only % disturbed area, are used.

Thus, I suggest to implement the following two changes in the ms:

1) The discussion on diversity patterns in dendritic networks is still rather sparse, and not very contemporary, most of this research has been post-2010, yet for example, the manuscript mostly refers to a text book by Allan (2007), which is a more generic reference on riverine systems, yet most theoretical work on dendritic networks and biodiversity patterns therein is from thereafter (e.g., Muneepeerakul et al. 2008 Nature, or Altermatt 2013 for a review).
Importantly, there are even specific models that predict that the expected diversity patterns are inverse when there is disturbances (see Harvey et al. 2018 for models and predictions under undisturbed and disturbed scenario), which would be very much in line with what the authors found here. I would strongly recommend to consider this aspect, as it will allow a much better embedding of the interesting findings in the context of the extensive work on dendritic networks (Lines 268–273 and elsewhere). Specifically, the inverse gradient of richness with respect to network position (Fig. S7) is predicted by Harvey et al. 2018 under disturbances in a theoretical model, thus would actually very much support the empirical findings presented here (this may even be among the first empirical demonstrations thereof, a nice point to add).

Muneepeerakul et al. (2008) Neutral metacommunity models predict fish diversity patterns in Mississippi-Missouri basin. Nature, 453(7192): 220-222.

Altermatt F (2013) Diversity in riverine metacommunities: a network perspective Aquatic Ecology, 47: 365-377.

Harvey et al. (2018) Disturbance reverses classic biodiversity predictions in river-like landscapes. Proceeding of the Royal Society B-Biological Sciences, 285: 20182441.

Thank you for those relevant references that were added in the manuscript (Lines 262-263 and 501-503) and especially in the paragraph discussing the patterns of diversity distribution obtained in our study (discussion, lines 176-186). The observed reverse pattern of fish diversity along the upstream-downstream gradient is indeed an important result as it indicates that anthropogenic disturbances can disrupt the classical expected pattern of biodiversity distribution in dendritic networks,
We believe these results are meaningful to highlight the particular response (and therefore sensitivity) of biodiversity inhabiting dendritic network ecosystems. The suggested literature was very helpful to support our findings by providing a mechanistic explanation to this observed reversed pattern of diversity distribution and better integrate our work to the extensive and recent work on dendritic networks

2) Further, I found that the answer to my previous comments 1 (I was reviewer #3 in the previous round) was good in the response letter, however, I would expect that this additional analysis and

information is also given in the manuscript, which was not the case. I had asked what the effects are when there is not % but absolute area deforested looked at. Indeed, there is a significant difference in the findings with the absolute values having much lower R2, and not stabilizing than when using % of land-change (detailed by the authors in the response letter). I think this is highly relevant, yet not clearly stated in the manuscript. I respect the authors decision to stick with the percentage as their main finding, but would expect that in the main manuscript this additional analysis (with absolute area, instead of % used) is mentioned, saying that it gives a different result (these results and figures, as they are now given in the response letter, should then be added to the supplement. I was a bit surprised to see these additional analyses and their discussion in the response letter, yet not being mentioned in the main manuscript and/or supplement. Thus, I would expect that next to Figure 3 given in the main ms, the paralleling figure given as a response to my comment 1 in the response letter is also added to the manuscript (supplement), and referred to in the main text.

We added the figure relating biodiversity and absolute values of deforested areas in the supplementary materials (Fig. S7). In the Materials and Methods section, we included the explanation provided in our previous response to your comment (Lines 416-425). We agree this comparison between absolute deforestation and percentage of deforestation improve the presentation of our approach as it helps justifying the choice of percentage rather than absolute values of deforested areas.

3) as a minor comment: the three sequencing platforms used are not "randomly distributed along the river courses" (line 372, see also Fig. S5), there was two platforms used for one stream (MiSeq and NextSeq), on two platforms used for another stream (Hiseq_2500 and Miseq). Thus, there was a very strong blocking (i.e., non-independence) in platforms used between the two streams. This is ok, but should be mentioned specifically. Saying "randomly" would be inadequate.

Yes, you are right. We replaced this sentence by "As different sequencing platforms were used (MiSeq and NextSeq for the Maroni and HiSeq 2500 and MiSeq for the Oyapock; see Fig. S6 and Table S6), the possible influences of the platforms on the sequencing results were verified".

Again, I would like to congratulate the authors for their interesting manuscript, and I am looking forward to see the remaining comments being resolved.

Thank you very much. Your constructive comments greatly contributed to the quality of our manuscript.

Reviewers' Comments:

Reviewer #1:
Remarks to the Author:
I have reviewed ms NCOMMS-21-20430B. I very much appreciate that the authors seem to have taken seriously my previous and many suggestions to better explain the research, its results, and perhaps more importantly frame the research and its results in a broad context of threats to and conservation of tropical ecosystems.

This ms now shows clearly that relatively small human disturbances in these ecosystems are propagated via hydrological connectivity and produce relatively large impacts on biodiversity measures. Obviously, what is large or small human disturbance or impact is all relative, but I feel that the overall message remains valid -- that, as the authors synthesize it, forest losses of <11% produce biodiversity losses of species richness of >22%. These results are framed in light of prevailing and up to date thinking of tropical conservation, and the discussion I found to be particularly well done in terms of considering all major points.

I would have only small suggestions in terms of the writing and presentation of ideas, but those I recognize are a matter of personal styles, so that I am here going to recommend acceptance as is.

Reviewer #4:
Remarks to the Author:
26-27: Please be more explicit. What influence did hydrologic connectivity have on biodiversity?

27-29: As these are correlations, please refrain from stating causations. This is one interpretation of the data and would require further testing to verify.

49-50: Theoretical work regarding network connectivity generally predates ecological assessments, which until recently have largely been focused on mainstem dynamics.

Fagan, W. F. (2002). Connectivity, fragmentation, and extinction risk in dendritic metapopulations. Ecology, 83(12), 3243–3249. doi:doi:10.1890/0012-9658(2002)083[3243:CFAERI]2.0.CO;2

286-290: I find it a bit hard to believe that 34 L was able to pass through a 0.45um filter without clogging the filter.

293-295: Please indicate if this is a fixative or lysis buffer. The term ‘conservation buffer’ is not informative or a common term.

349-352: Why species numbers and not OTUs/ASVs? The issue would be in the sequence variants that were generated not the final species counts after cleanup.

Bootstrapping of what? This is a very vague statement. I realize you have a supplement, but this gives little indication of what was done that could otherwise be reworded into a concise informative sentence or two.

384-386: I would suggest including a denoising stage in the bioinformatics to generate ASVs, especially with the use of multiple runs to generate the amplicons. This also gives the data some sense of tractability that is lacking with the OTU approach.

416-420: The issue with this is restricting the data to proportional data when used in your subsequent models. It is understandable why the data were transformed as such, but it may be less realistic compared to scaling the absolute deforested area as an alternative.

429-430: It is not clear what the model structure or parameters are for these outputs.

460-461: The functional diversity needs to be explicitly explained. There are many different definitions for this and this could be something as simple as simply body size for example or complex feeding groups. What is presented below is not functionality, but rather phenotypic variation. Many morphological characteristics are not indicative of functional differences and several will co-vary, which can cause type-1 errors in models.

498-500: Is there a reason for the two different models, the richness and functional response variables are both count data, correct? One would assume a Poisson distribution would be suited for both.

Results – refrain from interoperative statements in the results section, which should be incorporated into the discussion. There needs to be full set of statistics results presented in the results, which are oddly lacking.

119-121: Species richness consisted of count data, so why are proportional data being used to report the results here?

122-142: Provide the statistical results for the PCoA.

From the figures there seems to be a disproportionate number of sites with 0 deforestation. This causes an unbalanced sampling design which generally violates heterogeneity assumptions required for glm and lm models. Consider incorporating using some form of zero-inflated model correction or utilize models that can handle zero inflated data.

176-178. It would be more informative ecologically to use actual numbers of fish species versus percentages.

Most of the discussion is speculative and does not relate to the findings of the study.

208-211: I am generally confused as to how connectivity is related to this study. The majority of the sampling points occur on the main channel of the river and the key explanatory variable was deforestation measured from gis data. I apologize if I am missing something, but the way the term is presented seems like a stretch.

217-219: You also clearly show that the variation at 0 deforestation covers the full variation for all other deforestation levels.

Figure 5. Provide the proportion of explained variation for each of the axes

Dear Opale,

Your manuscript entitled "Low level of anthropisation causes harsh biodiversity decline in Amazonia" has now been seen again by our referees, whose comments appear below. Reviewer 3 was not available, therefore we recruited a new referee to replace them. In light of the advice received, I am delighted to say that we are happy, in principle, to publish a suitably revised version in Nature Communications under the open access CC BY license (Creative Commons Attribution 4.0 International License).

We therefore invite you to revise your paper one last time to address the remaining concerns of our reviewers and our editorial requests in the attached documents. As you will see, Reviewer 4 has a number of comments on the manuscript, including some questions on the methods, that must be addressed. At the same time we ask that you edit your manuscript to comply with our policies and formatting requirements and to maximise the accessibility and therefore the impact of your work.

Please see the reports below and the attached documents. Failure to comply with our editorial requests will cause delays in accepting your manuscript. Please also see the *Nature Communications* formatting instructions for further information.

Thank you very much for offering us the opportunity to publish our manuscript in Nature Communications. We addressed all the remaining concerns of Reviewer 4 and all the editorial requests. Moreover, we edit the manuscript to comply with Nature Communications policies and formatting requirements.

SUBMISSION INFORMATION

In order to accept your paper, we require the following:

- A revised author checklist describing your response to our editorial requests (attached).
- A separate point-by-point response to the reviewers' comments, reproduced verbatim.
- The final version of your manuscript as a Word or LaTeX file, with all changes highlighted in the text and any tables prepared using the table menu in Word or the table environment in LaTeX.
- If using LaTeX, please use numerical references only for citations, and include the references within the manuscript file itself. If you wish to use BibTeX, please copy the reference list from the .bbl file, paste it into the main manuscript .tex file, and delete the associated \bibliography and \bibliographystyle commands.

- The complete author list provided in the manuscript file, which must match that given on our manuscript tracking system. The author list in the main manuscript file will be used during typesetting of your article.

- Production-quality versions of each figure as a separate file containing all panels. To ensure the swift processing of your paper, please provide the highest quality versions of your images and when combining different figure parts into one file for layout, use a vector-based application such as Adobe Illustrator or Microsoft Powerpoint. We recommend .ai, .eps, .pdf, .ppt. Figures divided into panels should be labelled with a lower-case, boldface 'a', 'b', etc. in the top left-hand corner. If resolution is not of sufficient quality, production of your paper will be held whilst replacement files are obtained. For detailed guidance on figure preparation, see https://www.nature.com/documents/aj-artworkguidelines.pdf
- Please note that we do not modify the text in figures to conform to style during the production process. Please ensure that your figures are presented accurately and adhere to the guidance provided.

- Any updated checklists that verify compliance with our research ethics and data reporting standards in PDF format.
- The final version of the Supplementary Information in one PDF file.
- Any Supplementary Movie, Audio, Data and Software submitted as separate files. Supplementary Data and Source Data must be provided as .xls, .xlsx or .zip files, while Supplementary Software must be supplied as .zip files.

** Please note that we do not edit Supplementary Information files; they must be finalised prior to acceptance of the paper. **

- If you wish, an interesting image (but not an illustration or schematic) for consideration as a Featured Image on the Nature Communications homepage. The file should be 1200x675 pixels in RGB format and should be uploaded as a Related Manuscript File. In addition to our home page, we may also use this image (with credit) in other journal-specific promotional material.
- Completed and signed copies of our Multimedia License to Publish (LTP) for any Featured Image suggestions (please use one form for each image and give a scientific description of the image in the 'title' field; do not use "Featured Image" as a title): http://www.nature.com/documents/snl-multimedia-ltp.docx

OPEN ACCESS

Nature Communications is a fully open access journal. Articles are made freely accessible on publication under a CC BY license (Creative Commons Attribution 4.0 International License). This license allows maximum dissemination and re-use of open access materials and is preferred by many research funding bodies.

For further information about article processing charges, open access funding, and advice and support from Nature Portfolio, please visit http://www.nature.com/ncomms/about/open-access

At acceptance, you will be provided with instructions for completing this CC BY license on behalf of all authors. This grants us the necessary permissions to publish your paper.

ORCID

Nature Communications is committed to improving transparency in authorship. As part of our efforts in this direction, we are now requesting that all authors identified as 'corresponding author' create and link their Open Researcher and Contributor Identifier (ORCID) with their account on the Manuscript Tracking System (MTS) prior to acceptance. ORCID helps the scientific community achieve unambiguous attribution of all scholarly contributions. For more information please visit http://www.springernature.com/orcid
For all corresponding authors listed on the manuscript, please follow the instructions in the link below to link your ORCID to your account on our MTS before submitting the final version of the manuscript. If you do not yet have an ORCID you will be able to create one in minutes.
https://www.springernature.com/gp/researchers/orcid/orcid-for-nature-research

IMPORTANT: All authors identified as 'corresponding author' on the manuscript must follow these instructions. Non-corresponding authors do not have to link their ORCIDs but are encouraged to do so. Please note that it will not be possible to add/modify ORCIDs at proof. Thus, if they wish to have their ORCID added to the paper they must also follow the above procedure prior to acceptance.
To support ORCID's aims, we only allow a single ORCID identifier to be attached to one account. If you have any issues attaching an ORCID identifier to your MTS account, please contact the Platform Support Helpdesk.

POLICIES

In recognition of the time and expertise our reviewers provide to Nature Communications's editorial process, as of November 2018, we formally acknowledge their contribution to the external peer review of articles published in the journal. All peer-reviewed content will carry an anonymous statement of peer reviewer acknowledgement, and for those reviewers who give their consent, we will publish their names alongside the published article. For more information, please refer to our FAQ page at https://www.nature.com/documents/ncomms-reviewer-information.pdf

Nature Portfolio journals encourage authors to share their step-by-step experimental protocols on a protocol sharing platform of their choice. Where such protocols are available, please provide a DOI or other citation details in the paper. Nature Portfolio's *Protocol Exchange* is a free-to-use and open resource for protocols; protocols deposited in *Protocol Exchange* are citable and can be linked from the published article. More details can found at https://www.nature.com/protocolexchange/about

SUBMISSION
Please use the following link to submit the required items:
https://mts-ncomms.nature.com/cgi-bin/main.plex?el=A6S7CMBL5C1MaBP2I2A9ftdPNO4U35YOBt0YjMwpb1QZ
** If you wish to forward this email to your coauthors, please delete the link above **

We hope to hear from you within a few weeks and look forward to seeing your revised manuscript.
Best wishes,
Walter

Walter S. Andriuzzi, PhD
Senior Editor, Nature Communications
https://orcid.org/0000-0002-1271-265X
REVIEWERS' COMMENTS

Reviewer #1 (Remarks to the Author):

I have reviewed ms NCOMMS-21-20430B. I very much appreciate that the authors seem to have taken seriously my previous and many suggestions to better explain the research, its results, and perhaps more importantly frame the research and its results in a broad context of threats to and conservation of tropical ecosystems.

This ms now shows clearly that relatively small human disturbances in these ecosystems are propagated via hydrological connectivity and produce relatively large impacts on biodiversity measures. Obviously, what is large or small human disturbance or impact is all relative, but I feel that the overall message remains valid -- that, as the authors synthesize it, forest losses of <11% produce biodiversity losses of species richness of >22%. These results are framed in light of prevailing and up to date thinking of tropical conservation, and the discussion I found to be particularly well done in terms of considering all major points.

I would have only small suggestions in terms of the writing and presentation of ideas, but those I recognize are a matter of personal styles, so that I am here going to recommend acceptance as is.

Thank you very much.

Reviewer #4 (Remarks to the Author):

26-27: Please be more explicit. What influence did hydrologic connectivity have on biodiversity?
Hydrological connectivity is defined as the water-mediated downstream transfer of matter, energy, and/or organisms transport of sediments, nutrients, chemicals and energy (Pringle, 2001). According to this, through hydrologic connectivity, disturbances in one part of a river catchment can affect the structural dynamics of water flow and physico-chemistry, as well as aquatic habitats in distant downstream parts of that catchment. Alterations in freshwater ecosystems may, in turn, affect the downstream structure and composition of the riparian vegetation and the terrestrial biodiversity it supports.
This was explained in the introduction (Lines 48-52) and in the discussion (Lines 186-189), but not in the abstract as editorial policy requires to keep it short. To avoid misunderstanding, we no longer refer to hydrological connectivity in the abstract.

27-29: As these are correlations, please refrain from stating causations. This is one interpretation of the data and would require further testing to verify.
We modified this sentence as the Editor suggested:
“The findings suggest that anthropization had a spatially extended impact on biodiversity.” (Line 31).

49-50: Theoretical work regarding network connectivity generally predates ecological assessments, which until recently have largely been focused on mainstem dynamics.

Fagan, W. F. (2002). Connectivity, fragmentation, and extinction risk in dendritic metapopulations. Ecology, 83(12), 3243–3249. doi:doi:10.1890/0012-9658(2002)083[3243:CFAERI]2.0.CO;2
Thank you for this comment, we agree that theoretical works on network connectivity preceded the applications to field and applied ecology. We rephrased these lines to clarify this and added this reference to the main text (Lines 51-54).

286-290: I find it a bit hard to believe that 34 L was able to pass through a 0.45um filter without clogging the filter.
We used a filtration capsule designed to filter 30 to 60 litres of water. Its filtration membrane had a surface of 500 cm2, increasing therefore the filtration volume capacity and reducing clogging compared to disk membrane filters or Sterivex filters. These filtration capsules have been used in many rivers from different regions of the world, and filtered volumes were similar to the ones we obtained in our study e.g.(Lyet et al., 2021; Pont et al., 2018). The surface of the filtration membrane is now indicated in the Methods (Line 307).

293-295: Please indicate if this is a fixative or lysis buffer. The term ‘conservation buffer’ is not informative or a common term.

We changed “conservation buffer” to “preservation buffer” as used in Bruce et al. 2021 (Line 313)

349-352: Why species numbers and not OTUs/ASVs? The issue would be in the sequence variants that were generated not the final species counts after cleanup.
We assessed the influence of the platforms on species number because all our analyses refer to this variable. Moreover, as we are dealing with species presence/absence data, our results are not affected by the number of OTUs per species.

Bootstrapping of what? This is a very vague statement. I realize you have a supplement, but this gives little indication of what was done that could otherwise be reworded into a concise informative sentence or two.
Thank you for this comment. We detailed the procedure in the main text (Lines 372-378) and avoid the use of the word “Bootstrapping” which indeed can be confusing. We now say:
“To this end, we compared the differences in species numbers between the sample replicates assigned to the same platform (accounting for replicate effect only) against those of the sample replicates assigned to different platforms (accounting for replicate and platform effects). As there were more sites with their two replicates sequenced with the same platform than sites with their replicates sequenced with different platforms (see Supplementary Figure 6), sites with replicates on the same platform were randomly selected for the comparisons. We repeated this procedure 50 times.”.

384-386: I would suggest including a denoising stage in the bioinformatics to generate ASVs, especially with the use of multiple runs to generate the amplicons. This also gives the data some sense of tractability that is lacking with the OTU approach.
Yes, this is what we did. The denoising stage is performed during the obiclean step. We use the OBITools program obiclean that identify ASVs that have likely arisen due to PCR or sequencing errors. It uses the information of sequence counts and sequence similarities to classify whether a sequence is a variant (“internal”) of a more abundant (“head”) ASV (Boyer et al., 2016). After this step, we matched the ASV with the reference database to obtain the taxonomic assignation for each ASV. This is now explained lines 411-416

416-420: The issue with this is restricting the data to proportional data when used in your subsequent models. It is understandable why the data were transformed as such, but it may be less realistic compared to scaling the absolute deforested area as an alternative.

We followed your suggestion and ran the analyses using scaled absolute deforested areas (see figure below). Results remained the same as using proportional data (see Figure 3), although determination coefficients were lower for fish using scaled data. We thus preferred to maintain proportional data.

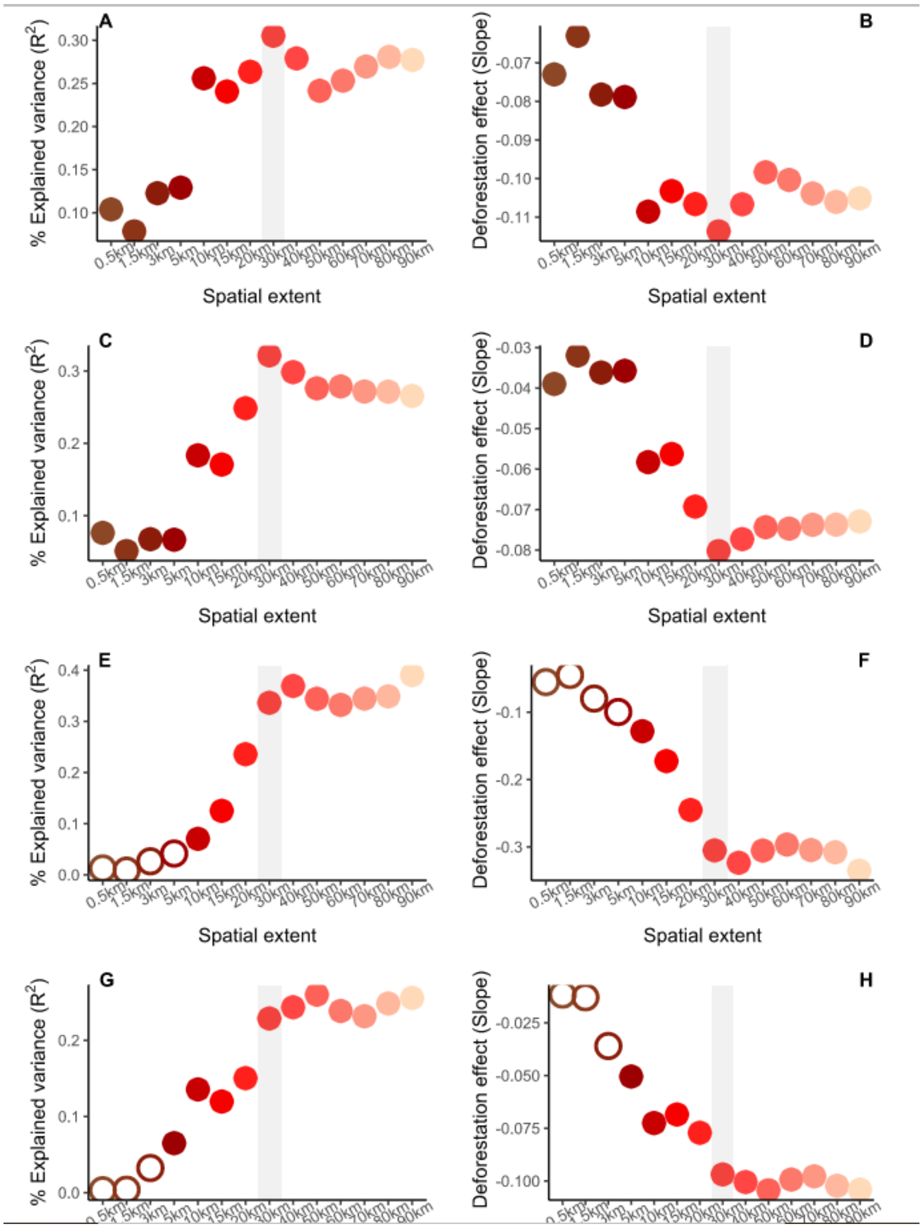

Results of the mixed models relating diversity measures and scaled absolute deforested areas upstream from the biodiversity sampling sites for each spatial extent. Left panels indicate variance explained by mixed models (R^2) for each spatial extent. Right panels indicate the strength of deforestation effect on biodiversity (slopes). Spatial extents account for deforested areas upstream from eDNA sampling sites. (a, b) Fish species richness (SR) models. (c, d) Fish functional richness (FR) models. (e, f) Mammal species richness models. (g, h) Mammal functional richness models. For each spatial extent, a specific generalized linear mixed model accounting for site network position and basin identity as random effects was built for each biodiversity facet. Significant ($p < 0.05$) and non-significant ($p > 0.05$) models assessed with Wald's tests are indicated by filled and open circles, respectively. Fish: n=64 sites and mammals: n=74. Colour shades are consistent with spatial extents. Grey vertical bars indicate models with highest R^2 or R^2 reaching a plateau, with less than 5% variation between successive spatial extents.

429-430: It is not clear what the model structure or parameters are for these outputs.
The models referred here have the same structure as the models used in the main text, but here the deforestation percentage was measured upstream and downstream the biodiversity study site (measured

only upstream for the models used in the main text). We modified these sentences to clarify the parameters used in these models:

"Similarly, the assessment of biodiversity responses to deforestation percentages measured upstream and downstream from the eDNA sampling disclosed only weak or non-significant relationships between deforestation and biodiversity (Supplementary Figure 9, see the Species and functional richness models section for details on model structure). For instance, the models that yielded significant ($p < 0.05$) results with upstream and downstream deforestation as explicative variable explained only <6% of the variance for both fish and mammals (Supplementary Figure 9)."(Lines 459-466).

460-461: The functional diversity needs to be explicitly explained. There are many different definitions for this and this could be something as simple as simply body size for example or complex feeding groups. What is presented below is not functionality, but rather phenotypic variation. Many morphological characteristics are not indicative of functional differences and several will co-vary, which can cause type-1 errors in models.

The measure of functional diversity is explained in the Methods (lines 476-521), and the full list of traits is available in the Supplementary Table 2. As explained in the "Biodiversity measures" section of the methods and in the Supplementary Table 2 we did not only considered morphological characteristics, but also ecological traits related to trophy, behavior, reproduction and habitat preference. In addition, the morphological traits that we used are associated to functions, notably locomotion and food acquisition strategies (see Villéger, Brosse, Mouchet, Mouillot, & Vanni, 2017 for a detailed description of the links between the morphological traits and their associated ecological functions).
The covariations should not be a problem here as we only selected traits with a correlation coefficient lower than 0.7 (see figure below that was added to the supplementary material as Supplementary Figure 10).

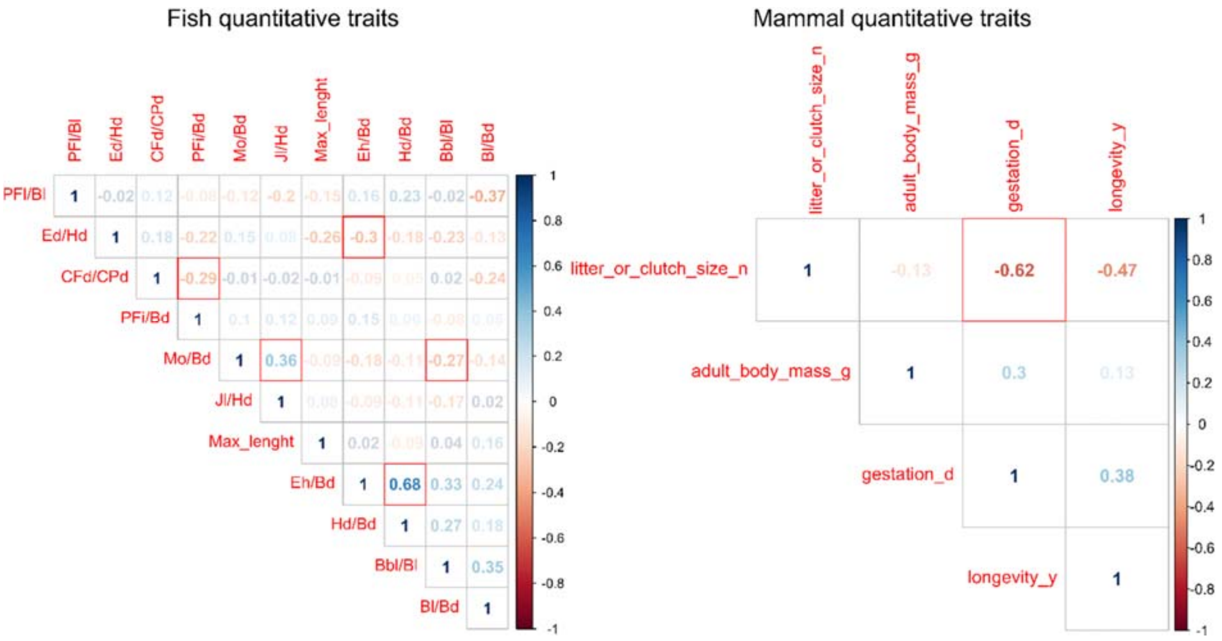

Supplementary Figure 10 Correlograms of the quantitative traits used to build the functional spaces and calculate functional richness for fish and mammals. Correlation coefficients (R) of pairwise combination of traits were obtained with Kendall non-parametric statistical tests. Only R values circled in red are statistically significant.

498-500: Is there a reason for the two different models, the richness and functional response variables are both count data, correct? One would assume a Poisson distribution would be suited for both.
We modelled the number of species with a Poisson distribution because it is count data.
The functional index corresponds to a continuous variable ranging between 0 and 1, and we therefore used Linear Mixed Models assuming a normal distribution for functional richness models. We now specified the nature of the two variables in the maint text: "Generalised linear mixed models (GLMM) with Poisson's distributions were used for species richness as species richness is a count variable. Linear mixed models (LMM) were used for functional richness as this variable is continuous and ranges between 0 and 1. As few

sites had high upstream deforestation values and several sites had deforestation values close to 0, upstream deforestation was square-root transformed to down-weight the few high deforestation values" (Lines 537-542).

Results – refrain from interoperative statements in the results section, which should be incorporated into the discussion. There needs to be full set of statistics results presented in the results, which are oddly lacking.
We have rewritten the results section according to the suggestions of the previous reviewers during the two previous rounds of revision. Specifically, Reviewer #1 was very concerned by "an excessive focus on statistics and not on the research questions" in the results. He was considering that the "focus on stats is hiding the ecological relevance of the research". We thus modified this section to highlight the ecological relevance of our results, and comply with this major concern of Reviewer #1. We nevertheless maintained the key statistics in the Results section and provided the full statistical results in the figures and in the supplementary materials.

For instance, the statistics of the models relating biodiversity to deforestation at each spatial extent are available in Figures 3 and 4, as well as in Supplementary Table 1, which provides all the R^2, p.values and slopes of the models. This is now clearly stated Lines 110-111.

The Kruskal-Wallis statistics of the comparisons of biodiversity between deforested and non-deforested sites are indicated in the results (Lines 132-134).

For the fitting of traits onto the Principal Components Analysis build using the functional traits, the statistics are indicated in the Supplementary Table 2. In a previous version of the manuscript, we listed the traits that significantly contributed to the ordination but reviewers asked us to simplify this section.

119-121: Species richness consisted of count data, so why are proportional data being used to report the results here?
We now report the results using both the number of species and proportional data. We agree that it is important to report the number of species not found in anthropized sites, but using percentages of species loss is also useful, as it allows comparing our results to those of other studies reporting changes in biodiversity in other localities or ecosystems (Lines 136-138 and 198-199).

122-142: Provide the statistical results for the PCoA.
Those results are provided in the supplementary Table 2, as indicated lines 142 and 154. We refrained from providing all the statistics in the Results section to comply with the major comments of Reviewers in a previous round of review. We nevertheless added the percentage of variance explained by each axis in the result section (Lines 141 and 152-153).

From the figures there seems to be a disproportionate number of sites with 0 deforestation. This causes an unbalanced sampling design which generally violates heterogeneity assumptions required for glm and lm models. Consider incorporating using some form of zero-inflated model correction or utilize models that can handle zero inflated data.

Zero inflated model correction is used to handle response variables, but to our knowledge, it is not used for explicative variables as there is no assumption on the distribution of the explicative variables in linear regressions. In our models the response variable is the biodiversity measure (species or functional richness), and deforestation is the explicative variable. Zero inflated model correction thus does not apply to our data. Moreover, only a few sites had deforestation values of 0. From buffers 3 to 90 km, only 5 sites had deforestation values of 0.
We nevertheless provide complementary analyses testing the assumptions of the linear regressions (homoscedasticity and normal distribution of residuals). These analyses constitute the validation process of the models presented in the main text (Fig. 4) and are now provided in the supplementary material. For all the models, the homoscedasticity and the normal distribution of the residuals was verified to the exception of the fish species richness model (panel a, b). The non-normal distribution of the residuals for this model was due to the presence of an outlier (standardized residuals = -5.43 related to the site M30). After removing this outlier, the distribution of the residuals for the fish species richness model becomes normal. These results can now be found in Supplementary Figure 1.

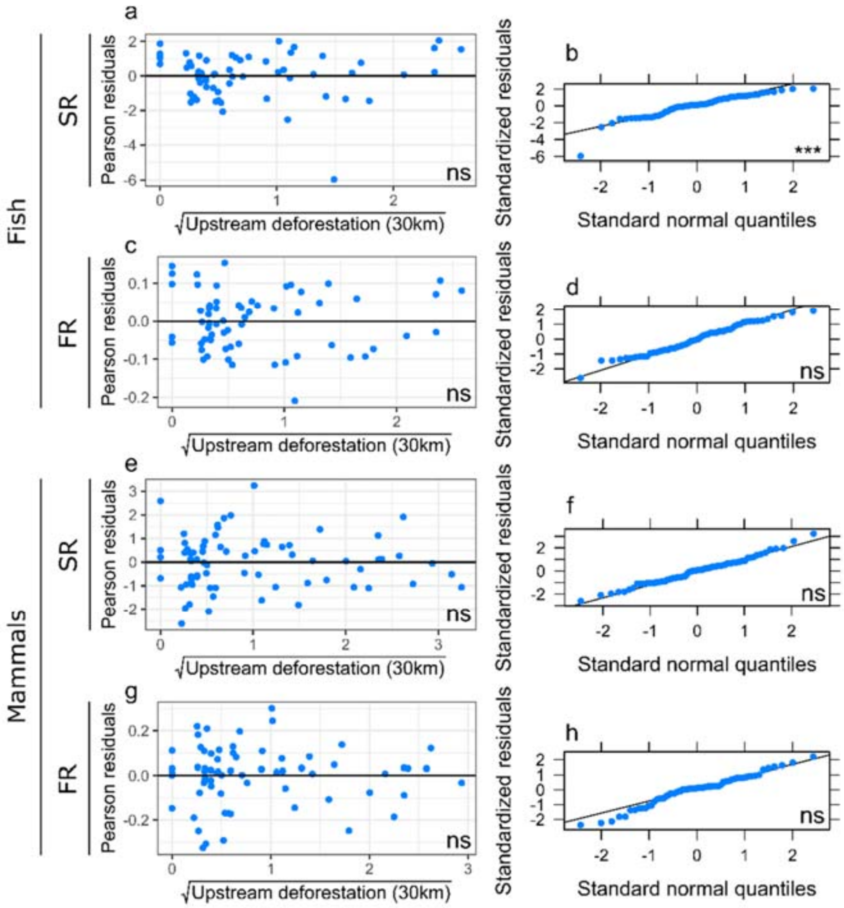

Supplementary Figure 1 Relationship between residuals of the generalized mixed models and the upstream deforestation measured up to 30km upstream from biodiversity sampling sites (see Fig. 4). Homoscedasticity, equal variance of the residuals along the regression line (a, c, e, g). A generalised additive model was fitted onto the residuals to test for significant residual patterns. Results that are not significant indicate the absence of residual pattern. ns = not significant. Quantile-quantile plot of the residuals (b, d, f, h). Normality of the residuals was assessed with Shapiro-Wilk tests. Results that are not significant indicate that residuals are normally distributed. ns = not significant, $p < 0.001$***. Fish: n=64 sites and mammals: n=74. The residuals of the models were normally distributed and showed no residual patterns to the exception of the fish species richness model (a, b). For this model, deviation from normality was due to a single outlier (corresponding to site M30, see Fig.1). Excluding this outlier from the analysis allows retrieving a normal distribution of the residuals.

176-178. It would be more informative ecologically to use actual numbers of fish species versus percentages.
Yes, we now refer to the actual number of species (line 198-199).

Most of the discussion is speculative and does not relate to the findings of the study.
Based on the author's guidance provided by the editor and your comments, we rephrased the statements making firm assertions of causality. We carefully checked the discussion and confirm it relates to the findings of the study that are discussed according to the literature. We nevertheless agree that the section of the discussion that reports the potential applications of our findings to biodiversity conservation remains speculative (lines 215-235). Reviewer 1 specifically requested this section in the previous rounds of review, and we took attention to developing this section without overstating our results. We do not provide unsupported affirmations but just explore potential implications of our results, as requested by Reviewer 1. We also included a discussion on environmental DNA metabarcoding to answer a demand of Reviewer 3. We believe this paragraph is relevant and legitimate as this method remains new and its validity needs to be argued to give credits to the ecological results obtained with this method.

208-211: I am generally confused as to how connectivity is related to this study. The majority of the sampling points occur on the main channel of the river and the key explanatory variable was deforestation measured from gis data. I apologize if I am missing something, but the way the term is presented seems like a stretch. We did not measured connectivity; connectivity is the interpretation explaining how remote deforestation can have far-reaching effects on the downstream biodiversity as it modifies water flow, sediment transport, water quality, and aquatic food webs. Here, because most of our sites are located on the main channels, we expect hydrologic connectivity along the upstream-downstream gradient to influence the observed distribution patterns of the mammal and fish faunas. This was assessed by considering deforestation affecting the upstream area from the biodiversity sampling sites or both upstream and downstream from the sampling sites, and only upstream deforestation was linked to biodiversity (Supplementary Figure 9).

217-219: You also clearly show that the variation at 0 deforestation covers the full variation for all other deforestation levels.

Yes it's true, and this is now noticed line 128-129. We do not expect biodiversity to be the same in all non-anthropized sites, because natural environmental conditions, such as the site position in the upstream-downstream gradient can greatly affect biodiversity. This is noticed lines 199-202 and 544-547. However, despite this natural spatial variability in biodiversity, we still detect a significant negative link between biodiversity and anthropization strength.

Figure 5. Provide the proportion of explained variation for each of the axes
The proportion of variance explained for each axis is provided in Supplementary Table S2a and was added in the results section (Line141 and 152-143).

References

Boyer, F., Mercier, C., Bonin, A., Le Bras, Y., Taberlet, P., & Coissac, E. (2016). obitools: A unix-inspired software package for DNA metabarcoding. *Molecular Ecology Resources*, *16*(1), 176–182.

Bruce, K. *et al. A practical guide to DNA-based methods for biodiversity assessment*. (2021). doi:10.3897/ab.e68634.

Lyet, A., Pellissier, L., Valentini, A., Dejean, T., Hehmeyer, A., & Naidoo, R. (2021). EDNA sampled from stream networks correlates with camera trap detection rates of terrestrial mammals. *Scientific Reports*, *11*(1), 11362. doi: 10.1038/s41598-021-90598-5

Pont, D., Rocle, M., Valentini, A., Civade, R., Jean, P., Maire, A., … Dejean, T. (2018). Environmental DNA reveals quantitative patterns of fish biodiversity in large rivers despite its downstream transportation. *Scientific Reports*, *8*(1). doi: 10.1038/s41598-018-28424-8

Villéger, S., Brosse, S., Mouchet, M., Mouillot, D., & Vanni, M. J. (2017). Functional ecology of fish: Current approaches and future challenges. *Aquatic Sciences*, *79*(4), 783–801. doi: 10.1007/s00027-017-0546-z
